# SalmoSim: the development of a three-compartment *in vitro* simulator of the Atlantic salmon GI tract and associated microbial communities

**DOI:** 10.1186/s40168-021-01134-6

**Published:** 2021-08-31

**Authors:** Raminta Kazlauskaite, Bachar Cheaib, Chloe Heys, Umer Zeeshan Ijaz, Stephanie Connelly, William Sloan, Julie Russel, Laura Rubio, John Sweetman, Alex Kitts, Philip McGinnity, Philip Lyons, Martin Llewellyn

**Affiliations:** 1 Institute of Biodiversity, Animal Health and Comparative Medicine, Graham Kerr Building, University of Glasgow, Glasgow, G12 8QQ Scotland; 2 School of Engineering, University of Glasgow, Glasgow, G12 8QQ Scotland; 3 Mowi ASA, Bergen, Norway; 4 Alltech Aqua, Eindhoven, Netherlands; 5 Alltech, Lexington, KY USA; 6 School of Biological, Earth and Environmental Sciences, University College Cork, T23 N73K Cork, Ireland; 7 Marine Institute, Foras na Mara, F28 PF65 Newport, Ireland

**Keywords:** SalmoSim, Gut microbiota, *In vitro* gut model system, Atlantic salmon, NGS

## Abstract

**Background:**

The aquaculture sector now accounts for almost 50% of all fish for human consumption and is anticipated to provide 62% by 2030. Innovative strategies are being sought to improve fish feeds and feed additives to enhance fish performance, welfare, and the environmental sustainability of the aquaculture industry. There is still a lack of knowledge surrounding the importance and functionality of the teleost gut microbiome in fish nutrition. *In vitro* gut model systems might prove a valuable tool to study the effect of feed, and additives, on the host’s microbial communities. Several *in vitro* gut models targeted at monogastric vertebrates are now in operation. Here, we report the development of an Atlantic salmon gut model, SalmoSim, to simulate three gut compartments (stomach, pyloric caecum, and midgut) and associated microbial communities.

**Results:**

The gut model was established in a series of linked bioreactors seeded with biological material derived from farmed adult marine-phase salmon. We first aimed to achieve a stable microbiome composition representative of founding microbial communities derived from Atlantic salmon. Then, in biological triplicate, the response of the *in vitro* system to two distinct dietary formulations (fishmeal and fishmeal free) was compared to a parallel *in vivo* trial over 40 days. Metabarcoding based on 16S rDNA sequencing qPCR, ammoniacal nitrogen, and volatile fatty acid measurements were undertaken to survey the microbial community dynamics and function. SalmoSim microbiomes were indistinguishable (*p* = 0.230) from their founding inocula at 20 days and the most abundant genera (e.g., *Psycrobacter, Staphylococcus, Pseudomonas*) proliferated within SalmoSim (OTUs accounting for 98% of all reads shared with founding communities). Real salmon and SalmoSim responded similarly to the introduction of novel feed, with majority of the taxa (96% Salmon, 97% SalmoSim) unaffected, while a subset of taxa (e.g., a small fraction of *Psychrobacter*) was differentially affected across both systems. Consistent with a low impact of the novel feed on microbial fermentative activity, volatile fatty acid profiles were not significantly different in SalmoSim pre- and post-feed switch.

**Conclusion:**

By establishing stable and representative salmon gut communities, this study represents an important step in the development of an *in vitro* gut system as a tool for the improvement of fish nutrition and welfare. The steps of the system development described in this paper can be used as guidelines to develop various other systems representing other fish species. These systems, including SalmoSim, aim to be utilised as a prescreening tool for new feed ingredients and additives, as well as being used to study antimicrobial resistance and transfer and fundamental ecological processes that underpin microbiome dynamics and assembly.

Video abstract

**Supplementary Information:**

The online version contains supplementary material available at 10.1186/s40168-021-01134-6.

## Background

In the last 50 years, per capita fish consumption has almost doubled from 10 kg in the 1960s to over 19 kg in 2012 [1]. This increase in the demand for fish protein has put wild fish stocks under pressure. The aquaculture sector now accounts for almost 50% of all fish for human consumption and is anticipated to provide 62% by 2030 [2]. Freshwater fish species currently dominate aquaculture fish production, such as freshwater carps and cyprinids, which constitute over 53% of total fish production by mass, with tilapia and other cichlids (11.0%) [3]. However, when reported by value, Atlantic salmon (*Salmo salar*) ranked highest [4]. While husbandry and disease control have advanced to improve fish health and welfare, improved feeds and their additives are the fundamental means of enhancing fish performance in aquaculture [5]. To improve aquaculture environmental and financial sustainability, the ratio of the marine origin components (Fishmeal (FM) and Fish Oil (FO)) within feeds has reduced considerably. For example, in Norway the ratio of the marine origin components within farmed salmon feed reduced from around 90% in 1990 to 30% in 2013 [6].

Recent studies suggest that teleost microbiome plays a vital role in fish’s health and performance [7, 8], and that alternative feeds, such as ones containing non-marine dietary ingredients, can result in poor fish growth, altered gut health alongside a modified fish gut microbial community composition and activity [9–11]. For instance, Atlantic salmon feed supplementation with dietary soybean protein concentrate can induce intestinal disorder [12]. Concomitant alterations in gut microbiota can result in the undesirable fermentation of various feed components [12, 13]. In view of all this, considerable interest lies around the development of novel ingredients and additives to enhance the performance of many species of farmed fish and their associated microbes.

To study the impact of novel feed ingredients on gut microbial communities (e.g., Gajardo et al., 2017), as well as the supplements (e.g., prebiotics, probiotics) tailored to modify microbial community diversity and function (e.g., Gupta et al. [14]), *in vivo* trials are widely deployed in aquaculture. Although physiologically relevant, *in vivo* trials have several scientific, ethical, and practical disadvantages. In salmonids, for example, gut sampling is terminal, preventing the generation of time series data from individual animals/microbial communities. Furthermore, microbial impacts on feed ingredients cannot be subtractively isolated from host enzymatic/cellular activity. From an ethical perspective, *in vitro* models offer the opportunity to reduce harm via the replacement of *in vivo* models [15]. Practically, *in vivo,* testing of novel feed ingredients is both time consuming and costly. A reliable *in vitro* model could offer advantages in this respect. To the best of our knowledge, there is only one other gut system in place simulating a generalised teleost gut, (‘fish-gut-on-chip’ [16]). The ‘fish-gut-on-chip’ system exploits microfluidic technology and is based on the reconstruction of the rainbow trout's intestinal barrier by culturing only intestinal cell lines in an artificial microenvironment and currently does not involve microbial communities isolated from the fish’s gut.

Prior to deploying an *in vitro* gut microbiome simulator to perform biological experiments, several criteria must be met. Firstly, steady-state microbial communities need to be established prior to the experimental procedure to ensure that results due to experimental treatments are not confounded with bacterial adaptation to the *in vitro* environment [17]. Secondly, physicochemical conditions within the artificial gut simulator and the gut of the target species should be similar. Thirdly, the bacterial communities need to be gut compartment–specific and representative of (if not identical to) the *in vivo* situation [18]. Finally, the *in vitro* gut simulator should be validated against a parallel *in vivo* experiment, to establish the degree to which the results from the experimental protocol within the artificial gut are generalisable to the *in vivo* situation [19]. Towards this end, several molecular techniques can then be deployed to analyse microbial populations within the gut. Multiplex quantitative PCR (qPCR) coupled with taxon-specific primers can rapidly detect and quantify the bacterial consortia within a large population [20]. Whilst shotgun metagenomics and amplicon sequencing approaches can provide a detailed taxonomic assessment of the microbial composition of the gut, they may be less useful for day-to-day monitoring of specific taxa [21].

In view of the above criteria, the aim of the current study is to develop a synthetic, continuous teleost gut microbial fermentation simulator. Teleosts are the largest and most diverse group of living vertebrates [22]. Due to the wide variety of habitats teleosts exploit and foods that they consume (ranging from bottom-living seaweeds and plankton organisms to actively swimming animals), fish have a wide array of gut morphologies [23]. Teleost stomachs can be classified into four general configurations: a straight stomach with enlarged lumen (e.g., northern pike (*Esox lucius*)), Y-shaped stomach (e.g., Nile tilapia (*Oreochromis niloticus*)), the absence of stomach (e.g., common carp (*Cyprinus carpio*)) and a U-shaped sack-like stomach with enlarged lumen, such as is found in Atlantic salmon, receiving food from the fish via the oesophagus [24, 25]. If an acidic stomach is present, it is responsible for initial unspecific digestion of incoming food by secretion of hydrochloric acid and endopeptidase pepsin from gastric mucosa glands in the stomach lining [26]. From the stomach, the food transport to the midgut is controlled by a muscular sphincter, the pylorus, which is developed to varying degrees in different species for unknown reasons, in some species even being absent (e.g., common carp in which the midgut attaches directly to the oesophagus) [27]. The pylorus has proven to be important in enzymatic breakdown of ingested macromolecules via the secretion of proteases (e.g., trypsinogen), glucosidases (e.g., α-amylase), and lipases (e.g., bile salt-dependent lipase)) [28] and to utilize the counter-current multiplication in generating osmoregulatory mechanisms for absorption of glucose, amino acids, dipeptides, and medium chain fatty acids [29–32]. The gastrointestinal tract ends with mid and distal intestines that further digest and absorb nutrients [26]. Thus, considering that Atlantic salmon has all three gut compartments, and is a leading species in worldwide aquaculture by value, it and its gut system, were chosen as a model for simulation *in vitro* in this study. Nonetheless, the principles and methodology described in this study could be applied to the development and construction of other systems representing different fish species as well.

Our experimental gut system simulates the stomach, the pyloric caeca, and the midgut regions of the gastrointestinal tract of the generalised marine lifecycle stage of the farmed Atlantic salmon. In this context, we first aimed to establish a stable gut community, representative of the salmon gut communities used to found it. Secondly, we validated the system as a potential means of testing the impact of feeds on salmon gut microbial communities by comparing the performance and response of the *in vitro* simulator during a feed trial with a parallel *in vivo* modulation of the gut microbial community in a cohort of marine-phase Atlantic salmon.

## Methods

### Experimental set-up and sample collection in an aquaculture setting

**Fig. 1:**
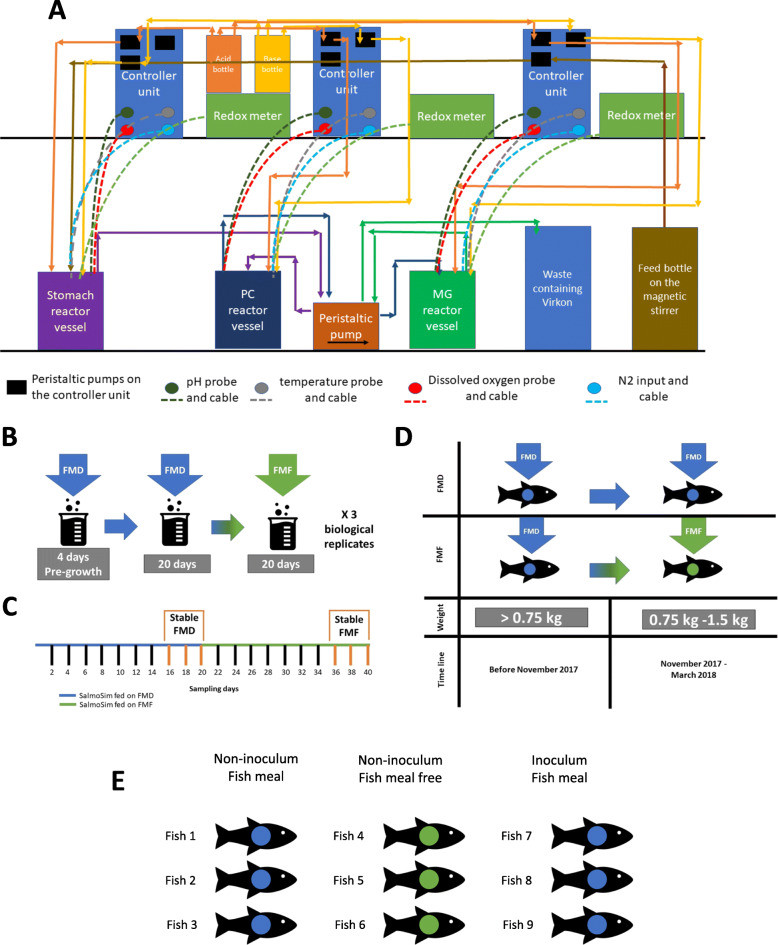
Salmon gut *in vitro* simulator. Schematic encompasses the artificial gut model system set-up, *in vivo* and *in vitro* feed trial set up. ***A*** is a schematic representation of SalmoSim system with transfer rate of 238 mL per day for each bioreactor. ***B*** SalmoSim feed trial design. ***C*** SalmoSim sampling time points, which include definition of stable time points (days 16, 18, and 20 for Fishmeal (once bacterial communities had time to adapt to SalmoSim system) and days 36, 38, and 40 for Fishmeal-free diet (once bacterial communities had time to adapt to change in feed)). ***D*** *in vivo* feed trial design. *FMD*, Fishmeal diet; *FMF,* Fishmeal-free diet. ***E*** Real salmon sacrificed for non-inoculum and inoculum samples (9 fish in total)

The Atlantic salmon (*Salmo salar*) *in vivo* feed trial was performed by MOWI ASA at their research site in Averøy, Norway. Prior to commencement of the feed trial, salmon were fed on a Fishmeal diet (FMD) until they reached *circa* 750 g in mass. Fish were separated into 5 × 5 m marine pens (150 randomly distributed fish per pen) in a 4 × 4 modular design. Four pens were randomly assigned to each of the trial diets. This study focused on eight pens housing fish fed on FMD and Fishmeal-free diet (FMF) (Table 1, Fig. 1D). The feed trial was conducted over 5 months (November 2017–March 2018). For *in vivo* samples recovered from actual salmon, three randomly selected fish were collected at the end of the feed trial for two different feeds (*N* = 3 fish/feed: Fish 1, 2, and 3 for FMD and Fish 4, 5, and 6 for FMF) and sacrificed by MOWI employees (Fig. 1E). After, 1 cm in length samples from three salmon gut compartments were collected (stomach (*N* = 3/feed) pyloric caeca (*N* = 3/feed) and midgut (*N* = 3/feed) (approximately 20 cm from the vent)), placed into 1.5-mL cryovials, and kept on ice before long-term storage in – 80 °C conditions. For *in vitro* initial inoculum samples (the founding community for SalmoSim runs), a further three fish fed on FMD were sacrificed (Fish 7, 8, and 9), and 5 cm in length samples from three distinct gut compartments were collected (stomach (*N* = 3), pyloric caecum (*N* = 3) and midgut (*N* = 3)), transferred to 15-mL Falcon tubes and kept on ice before long-term storage in – 80 °C conditions (Fig. 1E). Details of samples collected from farmed Atlantic salmon have been described previously [33].

**Table 1:** Fish meal and Fish meal free diets composition. Table summarises Fish meal and Fish meal free diets composition in percentage of the feed

	Fishmeal	Fishmeal-free
**Ingredient (% of the feed)**		
Fish meal	17.5	0
Soya protein concentrate	12	27.8
Corn gluten	7	7.35
Wheat gluten	10	14.34
Sunflower expeller	3.41	0
Wheat	4.81	11.22
Beans dehulled	10	0
Fish oil	15.68	16.99
Rapeseed oil	11.78	11.79
Linseed oil	3.05	3.2
Mannooligosaccharide	0.4	0.4
Astaxanthin	0.04	0.04
Crystalline amino acids	1.35	1.99
Mineral premixes	1.83	2.66
Vitamin premixes	0.6	0.73
**Macronutrients (% of the feed)**		
Moisture	5.9	6.13
Crude protein	39.1	40.1
Crude fat	33.4	33.3
Ash	5.47	4.2
Starch	9.4	11
Crude fibre	2	2.7

### Establishment of stable, representative gut communities in SalmoSim and subsequent feed trial

#### Physicochemical conditions within Atlantic salmon gastrointestinal tract and microbiome sampling

Physicochemical conditions (temperature, pH, dissolved oxygen) were directly measured in adult Atlantic salmon from MOWI salmon farm in Loch Linnhe, Scotland (Supplementary Figure 1A-C). Bacterial inoculums were prepared for the *in vitro* trial from the different gut compartments sampled from individual fish (three biological replicates, three gut compartments per fish—stomach, pyloric caecum, and midgut) collected at the start of the *in vivo* feed trial in Averøy, Norway. Prior to SalmoSim inoculation, inoculums that were stored in 15-mL Falcon tubes in − 80°C freezer were dissolved in 1 mL of autoclaved 35 g/L Instant Ocean® Sea Salt solution. Distinct individual fish collected in Averøy formed the founder community for each distinct replicate of the *in vitro* trial (i.e., a true biological replicate (Fig. 1E)).

#### *In vitro* system ‘feed’ preparation

*In vitro* system feed media was prepared by combining the following for a total of 2 L: 35 g/L of Instant Ocean® Sea Salt, 10 g/L of the FMD or FMF used in the MOWI feed trial (Table 1: the concentration of feed added was optimized to run through the system without clogging the narrow tubing), 1 g/L freeze-dried mucous collected from the pyloric caecum of Scottish marine-phase Atlantic salmon and 2 L of deionised water. This feed was then autoclave-sterilised, followed by sieving of the bulky flocculate, and finally subjected to the second round of autoclaving. This subjection of the feed to two rounds of autoclave should have degraded the vast majority of DNA, thus ensuring that feed microbiome will not influence the microbiome within the SalmoSim system.

#### *In vitro* system preparation

Three 500-mL Applikon Mini Bioreactors (one for each gut compartment: stomach, pyloric caecum, and midgut) were filled with four 1 cm^3^ cubes made from sterile aquarium sponge filters used as a surface for biofilm formation, assembled by attaching appropriate tubing and probes (redox, temperature, and dissolved oxygen; Fig. 1A), and autoclaved. Bioreactor preparation was followed by attachment of reactor vessels to the Applikon electronic control module, connection of feed and acid and base bottles (0.01 M hydrochloric acid and 0.01 M sodium hydroxide solutions filtered through a 0.22-μm polyethersulfone membrane filter unit (Millipore, USA)). Nitrogen gas was periodically bubbled through each vessel to maintain anaerobic conditions and dissolved oxygen continually monitored. The bioreactors were then allowed to fill with 400 mL of feed media. Once the system was set up, media transfer, gas flow, and acid/base addition occurred for 24 h in sterile conditions (without microorganisms present in the system) in order to stabilise the temperature, pH, and oxygen concentration with respect to levels measured from adult salmon.

#### Initial pregrowth period during *in vitro* trial

In order to allow bacterial communities to proliferate in the *in vitro* environment without washing through the system, the microbial populations within the inoculum from real salmon were pre-grown inside the SalmoSim system for 4 days. During this phase, the system was filled with FMD media preparation and inoculum, and no media transfer occurred.

#### Performing feed trial within SalmoSim system

After the initial pregrowth period, each validation experiment was run for 20 days while supplying the SalmoSim system with FMD. After 20 days, SalmoSim was run for 20 additional days while supplying FMF food. During the full 44-day experiment (4-day pregrowth period, 20-day system fed on FMD, and 20-day system fed on FMF) physiochemical conditions within three SalmoSim gut compartments were kept similar to the values measured in real salmon: temperature inside the reactor vessels were maintained at 12 °C, dissolved oxygen contents were kept at 0% by daily flushing with N_2_ gas for 20 min, and pH was kept stable in each bioreactor by the addition of 0.01 M NaOH and 0.01 M HCl (stomach pH 4.0, pyloric caecum pH 7.0, and mid intestine pH 7.6). During this experiment (apart from the pregrowth period), the transfer rate of slurry between reactor vessels was 238 mL per day as described *in vivo* [34, 35]. Finally, 1 mL of filtered salmon bile and 0.5 mL of sterile 5% mucous solution (both collected from Scottish marine-phase Atlantic salmon) were added daily to the reactor, simulating the pyloric caecum compartment. The schematic representation of the SalmoSim system is visualised in Fig. 1A, and full feed trial within SalmoSim is visually summarised in Fig. 1B and C.

Sampling was performed in several steps. First, samples from initial inoculums were collected for each gut compartment. Once SalmoSim main experiment started, the sampling from each bioreactor vessel was performed every second day throughout the 40-day run period (20 samplings in total). The SalmoSim samplings entailed collecting 30 mL of the bioreactor contents (10% of the total bioreactor volume—the maximum volume of sampling without disturbing microbial community structure) into 50 mL Falcon tubes, centrifuging them for 10 min at 5000 rpm speed, and freezing the pellets at – 20 °C for storage. The pellets were frozen to perform DNA extraction all together with the same kit (to prevent batch effect), and supernatant was used for VFA analysis.

### Nitrogen metabolism within the SalmoSim system

At each sampling point, the protein concentration in each chamber of the system was measured using Thermo Scientific™ Pierce™ BCA Protein Assay Kit (Thermo Fisher Scientific, USA) and the ammonia concentration using Sigma-Aldrich® Ammonia Assay Kit (Sigma-Aldrich, USA) to assay the bacterial community activity. Both methods were performed according to manufacturer’s protocol by using the Jenway 6305 UV/Visible Spectrophotometer (Jenway, USA). The same samples were used for both of these analyses immediately after sampling (no freezing or intermediate steps required).

### Volatile fatty acid (VFA) production in SalmoSim

The last two time points for each diet were selected from the SalmoSim system (for all three gut compartments) for VFA analysis—18 and 20 for FMD and time points 38 and 40 for FMF, respectively—to ensure that the bacterial communities had enough time to adapt to the SalmoSim system (for FMD time points) and the feed change (for FMF time points). During runs, 1 mL of supernatant from SalmoSim bioreactors was frozen in – 80 °C which was then used for VFA extraction. The protocol involved combining 1 mL of supernatant with 400 μL of sterile phosphate-buffered saline (PBS) solution (Sigma Aldrich, USA) and vortexing the mixture for 1 min. The sample was then centrifuged at 16,000 *g* for 30 min, followed by two rounds of supernatant removal, before centrifuging at 16,000 *g* for 30 min. Finally, the supernatant was then filtered through 0.2 μm Costar SpinX centrifuge tube filters (Corning, USA) at 15,000 *g* for 2 min until clear. The extracted VFAs were sent for gas chromatographic analysis at MS-Omics (Denmark). In order to determine if the VFA concentrations were statistically different between SalmoSim fed on FMD and FMF diets, measured VFA values dataset were subjected to statistical analysis using linear mixed effect models (See Supplementary methods 2). Results are shown in supplementary Figure 9.

### *In vivo* phenotypic fish performance fed on two different feeds

Phenotypic performance data (fish length, weight, gutted weight, carcass yield, gonad, and liver weights) was collected and provided at the end of the *in vivo* feed experiment by MOWI. The differences between each feed group (*n* = 32 fish per feed) for each phenotype were visualised, and statistical analysis was applied (independent two-sample *t* test) to identify statistically significant differences between the two feed groups.

### Measuring bacterial population dynamics in SalmoSim

#### Genomic DNA extraction

The DNA extraction protocol followed was previously described by [33]. In short, samples were subjected to a bead-beating step for 60 s by combining samples with MP Biomedicals™ 1/4" CERAMIC SPHERE (Thermo Fisher Scientific, USA) and Lysing Matrix A Bulk (MP Biomedicals, USA). Later, DNA was extracted by using the QIAamp® DNA Stool kit (Qiagen, Valencia, CA, USA) according to the manufacturer’s protocol [36].

#### NGS library preparation and sequencing

In the first instance, microbial population dynamics in SalmoSim were measured in near real-time using a set of qPCR primers including published and custom sequences to enable the stability of the system to be monitored (See supplementary Methods 1 and data Supplementary Figure 4). Subsequently, 16S rRNA sequencing was deployed to provide a fuller picture community dynamics. The commonly used 16S ribosomal hypervariable region 4 primers were shown to cross-amplify *Salmo salar* 12S ribosomal gene sequences [33, 37] and hence were not used in this study. Rather, amplification of the 16S V1 hypervariable region was adopted as an alternate approach [38]. Amplification of 16S V1 hypervariable region from diluted DNA samples was achieved using redundant tagged barcode 27F and 338R at final concentration of 1 pM of each primer. Primer sequences are summarised in Supplementary Table 2. First-round PCR was performed in triplicate on each sample, and reaction conditions were 95 °C for 10 min, followed by 25 cycles at 95 °C for 30 s, 55 °C for 30 s, and 72 °C for 30 s, followed by a final elongation step of 72 °C for 10 min. After the triplicate reactions were pulled together into one, their concentration was measured using a Qubit® fluorometer (Thermo Fisher Scientific, USA), and all of them were diluted to 5 ng/μL using Microbial DNA-Free Water (Qiagen, Valencia, CA, USA). The second-round PCR, which enabled the addition of the external multiplex identifiers (barcodes), involved only six cycles with otherwise identical reaction conditions to the first. The detailed composition of second-round PCR primers is summarised in Supplementary Table 3. This was followed by the DNA clean-up using Agencourt AMPure XP beads (Beckman Coulter, USA) according to the manufacturers' protocol. The cleaned DNA was then gel-purified by using the QIAquick Gel Extraction Kit (Qiagen, Valencia, CA, USA) and then quantified by using Qubit® (Thermo Fisher Scientific, USA). All the PCR products were pulled together at 10 nM concentration and sent for sequencing using HiSeq 2500.

### Bioinformatic analysis of 16S rRNA gene amplicon sequencing data

Sequence analysis was performed with our bioinformatic pipeline as described previously with slight modifications [33]. First, quality filtering and trimming (> Q30 quality score) was performed on all the reads of the 16S rRNA V1 hypervariable region using Sickle version V1.2 software [39]. Second, read error correction was performed using the BayesHammer module within SPAdes V2.5.0 software to obtain high-quality assemblies [40]. Third, paired-end reads were merged (overlap length 50 bp) using PANDAseq v2.11 software with a simple_Bayesian read merging algorithm [41, 42]. After overlapping paired-end reads, merged reads were dereplicated, sorted, and filtered chimaeras using the GOLD SILVA reference dataset [43], and singletons were removed by using the VSEARCH version 2.3.4 tool [44]. Merged paired-end filtered reads were clustered in operational taxonomic units (OTUs) using the VSEARCH software at 97% identity followed by a decontamination step by mapping OTUs against the host (*Salmo salar*) reference genome (available on NCBI) DNA using the BWA aligner implemented in the DeconSeq v0.4.3 tool [45]. Taxonomic assignment of OTUs was achieved using the Naïve Bayesian Classifier [46] implemented in the QIIME 2 platform using the SILVA 132 database [47, 48]. Phylogenetic trees of OTUs were generated using FastTree software after using MAFFT for multiple sequence alignment [49]. The resultant OTU table was converted to a biological observation matrix (BIOM) format for the post-OTU statistical analysis [50].

### Post-OTU statistical analysis: diversity metrics and community structure and composition analysis

All statistical analysis of the OTU table was performed by using R v 4.0.1 and RStudio v 1.3.959 [51]. Alpha diversity analysis was performed using Rhea pipeline [52], supplemented by microbiomeSeq [53], and PhyloSeq [54] for ANOVA and visualisation steps. Two alpha diversity metrics were calculated: microbial richness (estimated number of observed OTUs) and Shannon diversity (an estimate of balance of the community using the effective Shannon index [55, 56]. Before calculating effective microbial richness, proportional filtering was performed at a relative abundance of 0.25% in each community to minimise the inflation in microbial richness caused by the very low abundant OTUs. Afterwards, a one-way ANOVA of diversity between groups was calculated with the *p* value threshold for significance (*p* value < 0.05) represented using boxplots.

To investigate the effect of time on the bacterial community stability, beta diversity analysis was performed using different phylogenetical distance metrics to assay phylogenetic similarities between samples (weighted, generalised, and unweighted UniFrac). To compare communities isolated from various sources (SalmoSim, inoculum, and real salmon), only samples fed on FMD were included as initial inoculum were collected from fish fed on FMD alone. Furthermore, only SalmoSim samples from the last 3 time points fed on FDM were selected as they are considered stable time points (once bacterial communities had over 2 weeks to adapt and grow within the SalmoSim system). In short, the resulting dataset contained real salmon samples fed on FMD, all inoculum samples, and stable SalmoSim time points fed on FMD (days 16, 18, and 20). This dataset was then subdivided into several smaller datasets that included OTUs, shared by various percentages of samples (60%, 50%, 40%, and 30% of samples), with the aim of minimising the impact of rare OTUs (low prevalence) on comparisons and only focusing on prevalent OTUs between samples (see details in Supplementary Table 4).

To analyse the response of microbes to the diet change (see Table 1 for feed formulation) in real salmon and SalmoSim, in addition to the full dataset (*in vivo* and *in vitro* samples); three different full dataset subsets were used to perform beta diversity analysis: samples from *in vivo* study, all samples from SalmoSim (all data points), and samples only from SalmoSim once it had achieved stability (the last 3 time points fed on FMD (days: 16, 18, and 20) and FMFD (days 36, 38, and 40). These datasets were used to compute ecological (Bray–Curtis and Jaccard) and phylogenetic (unweighted, weighted, and generalised UniFrac) distances with vegdist function from the vegan v2.4-2 package and GUniFrac function (generalised UniFrac) from the Rhea package [52, 57]. Both ecological and phylogenetical distances were then visualised in two dimensions by Multi-Dimensional Scaling (MDS) and nonmetric MDS (NMDS) [58]. Finally, a permutational multivariate analysis of variance (PERMANOVA) by using both calculated distances was performed using adonis function to determine if the separation of selected groups was significant as a whole and in pairs [58]. The full beta diversity workflow is summarised in Supplementary Methods 3.

To provide an overall visualisation of microbial composition across all samples, a Principal Coordinates Analysis (PCoA) was performed using the microbiomeSeq [53] package based on the phyloseq package [59] with Bray–Curtis dissimilarity measures calculated and visualised for four different subset datasets: the full dataset (real salmon, inoculum, and all SalmoSim samples) and three different subsets each containing only one of the free biological replicate samples from SalmoSim (Fish 1, 2, or 3), along with all real salmon and inoculum samples.

Differential abundance was calculated by using microbiomeSeq based on the DESeq2 package [53, 59]. The BIOM-generated OTU table was used as an input to calculate differentially abundant OTUs between selected groups based on the Negative Binomial (Gamma-Poisson) distribution.

## Results

### Stabilisation of representative microbial communities within the SalmoSim system

**Fig. 2:**
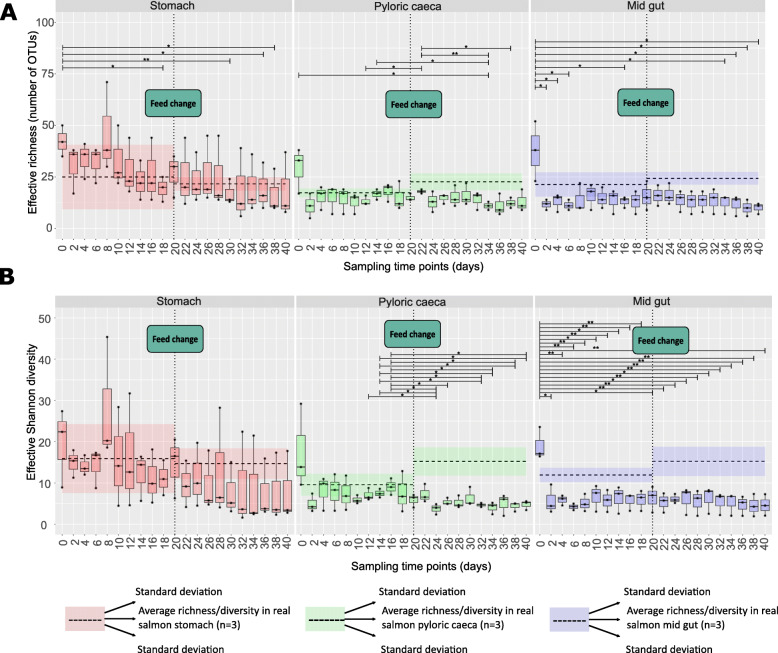
Calculated alpha-diversity metrics within SalmoSim system over time. The figure represents different alpha diversity outputs at different sampling time points (days) from SalmoSim system. Time point 0 represents microbial community composition within initial SalmoSim inoculum from the real salmon; time points 2–20 identify samples from SalmoSim system fed on Fishmeal diet, and time points 22–40 identify samples from SalmoSim system fed on Fishmeal-free diet. The dotted vertical line between days 0–20 represents average alpha diversity values measured in real salmon fed on Fishmeal diet, and dotted vertical line between days 22–40 represents average alpha diversity values measured in real salmon fed on Fishmeal-free diet. Finally, the horizontal dashed lined represents average effective richness (***A***) and effective Shannon diversity (***B***) in real salmon individual gut compartments fed on different diets (*n* = 3 fish/feed and gut compartment), and the shaded region around the horizontal dashed line represents the standard deviation of the values measured within real salmon samples fed on the different diets. **A** visually represents effective richness (number of OTUs), and **B** represents effective Shannon diversity. The lines above bar plots represent statistically significant differences between different time points. The asterisks flag the levels of significance: one asterisk (*) for *p* values between 0.05 and 0.01, two asterisks (**) for *p* values between 0.01 and 0.001, and three asterisks (***) for *p* values below 0.001

Effective richness (Fig. 2A) indicates that within the stomach and midgut compartments, the initial inoculum contained the highest number of OTUs compared with later sampling time points from the SalmoSim system: in the stomach compartment, effective richness was statistically different between time point 0 (initial inoculum derived from salmon guts) and time points 18, 30, 36, and 38, and within the midgut compartment, the number of OTUs within the initial inoculum (time point 0) was statistically different from time points 2, 4, 6, 16, 34, 36, 38, and 40. However, within the pyloric caeca compartment, only one time point (time point 34) had a significantly different number of OTUs from the initial inoculum (time point 0).

Our results reveal that within the stomach compartment over time (including initial inoculum), the effective Shannon diversity was stable with a downwards but nonsignificant trend over the course of the experiment (Fig. 2B). A similar downwards trend was observed in the pyloric caecum, with significant differences between later time points, but no significant differences were noticed between the inoculum and SalmoSim. Within the midgut compartment, Shannon diversity was statistically lower than the inoculum (time point 0) over most time points (sampling days 2–40).

Taken together, diversity and richness metrics suggest some loss of microbial taxa as a result of transfer of salmon gut communities into SalmoSim in the pyloric caecum and midgut, but not in the stomach. Subsequently, richness and evenness were then stable over the time course of the experiment in the stomach and midgut compartments (some instabilities seen only between the initial inoculum and later time points), whilst much more instability within the alpha diversity metrics were detected in the pyloric caecum compartment.

**Fig. 3:**
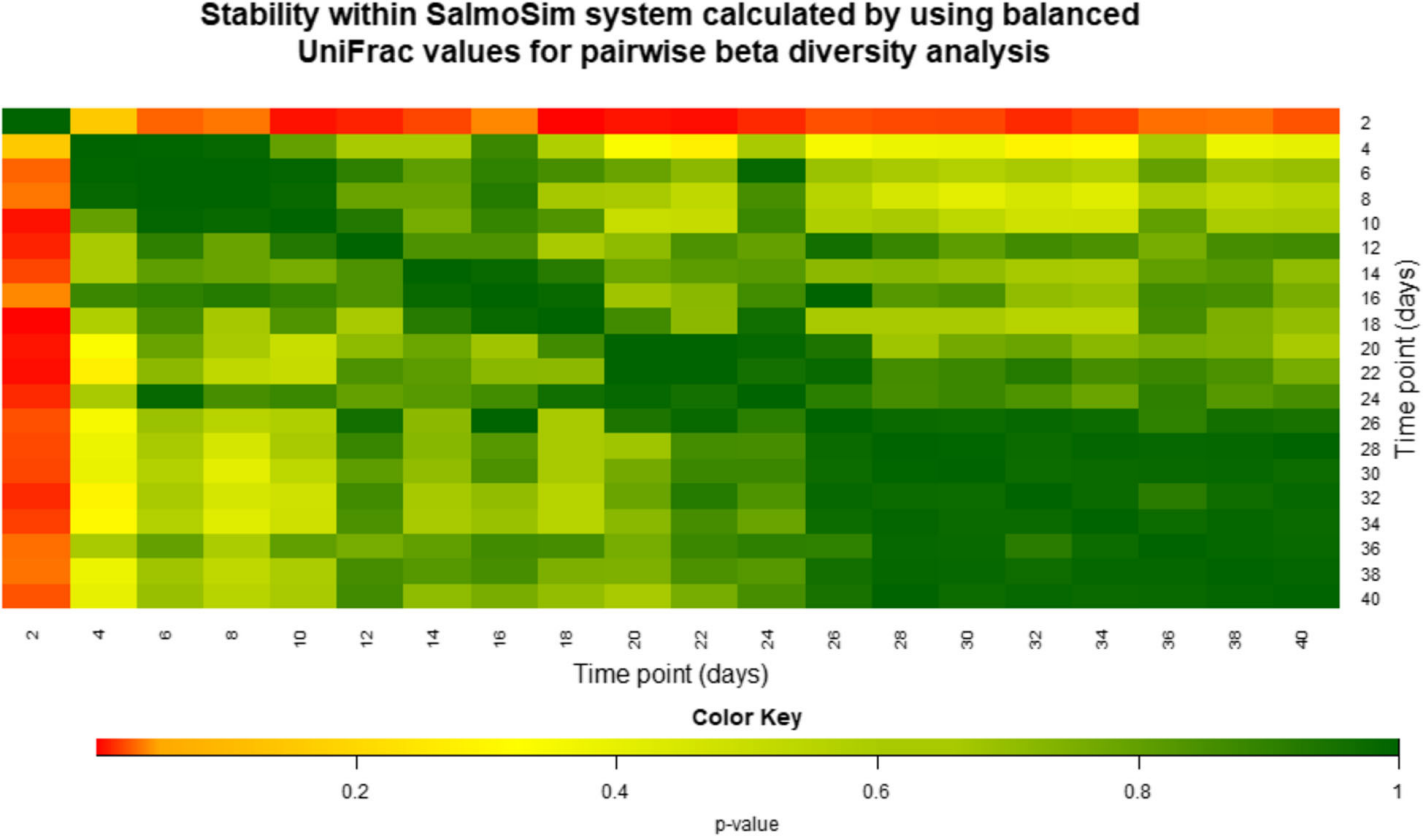
Stability within SalmoSim system calculated by using generalised UniFrac values for pairwise beta diversity analysis. The figure represents microbial stability within the SalmoSim system (data from all gut compartments combined) as the pairwise beta diversity comparison between different sampling time points (days), calculated by using generalised (50%) UniFrac as a distance measure. A small *p* value indicates that the two time points are statistically different, and *p* > 0.05 indicates that two time points are not statistically different. The colour key illustrates the *p* value: red end of spectrum denoting low *p* values (distinct compositions between time points) and dark green indicating high *p* values (similar compositions between timepoints)

To assess the compositional stability of the system, comparisons over time were undertaken with reference to pairwise beta diversity metrics. Significant differences in composition between time points represent instability in the system. Figure 3 visually summarises between–time point comparison of beta diversity metrics within the SalmoSim system across all replicates using generalised UniFrac (visual representations of the results using unweighted and weighted UniFrac are summarised in Supplementary Figure 3). Irrespective of the metric used, microbial community composition appeared to stabilise in all gut compartments by approximately 20 days, with little-observed impact of introducing the different feed at day 20. This trend was supported by our qPCR data, suggesting increasing stability over the course of the 40-day experiment (Supplementary Figure 4). Prevailing stability was also observed when each biological replicate’s individual gut compartment was examined separately (stomach, pyloric caecum, and midgut) (Supplementary Figure 5). Importantly, stabilisation over 20 days was a feature of two previous pilot runs of the system using unrelated marine salmon gut communities (Supplementary Figure 6).

### Microbial identity and diversity compared between SalmoSim and salmon

To compare microbial identity and microbiome composition between salmon and SalmoSim sample sizes were first balanced by examining a reduced dataset that contained real salmon samples (three fish individuals used to inoculate and three others, all fed FMD) and stable SalmoSim time points fed on FMD (days 16, 18, and 20). Alpha diversity comparisons between inoculum, real salmon, and SalmoSim are shown in Fig. 2.

Compositional comparisons between different sample types (inoculum salmon, three other individuals and SalmoSim) were made using several pairwise beta diversity metrics (phylogenetic (unweighted, generalised, and weighted UniFrac and ecological distances (Bray–Curtis and Jaccard)) (Table 2). Ecological metrics could not distinguish between SalmoSim (days 16, 18, and 20) from the individual fish used to inoculate the system, suggesting that microbiome composition was very similar between salmon and SalmoSim (Table 2). In contrast, metrics that incorporate phylogenetic differences between taxa (i.e., Unifrac) did identify significant differences, indicating that there is variability between the inoculum and SalmoSim, but the taxa involved are closely related. To explore the impact of rare OTUs when accounting for observed differences between sample types, the dataset was partitioned and analysed. Partitioned datasets indicated that progressive removal of rare OTUs increased the compositional similarity between SalmoSim and the fish gut communities used to inoculate the system (Table 2). Inoculum samples (18 samples) generated 388 OTUs, and SalmoSim stable samples (54 samples in total: SalmoSim time points fed on FMD (days 16, 18, and 20) and SalmoSim time points fed on FMF (days 36, 38, and 40)) generated 508 OTUs. A total of 291 OTUs were present in both sample types. Importantly, the shared 291 OTUs accounted for > 97% of the total reads in inoculum samples and > 98% in stable SalmoSim samples (Table 2), again suggesting that the majority of abundant microbes in real salmon were maintained in the SalmoSim system.

**Table 2:** Beta diversity comparisons of microbial composition between different samples (real salmon, inoculum, and SalmoSim)

Test		Data		Salmon vs SalmoSim		Salmon vs inoculum		SalmoSim vs inoculum	
**UniFrac**	**Unweighted (0%)**	**All**		0.001 ***		0.002 **		0.002 **	
		**Subset**		0.001 ***		0.002 **		0.001 ***	
		**Core OTUs**	**60%**	0.04 *		0.032 *		0.143	
			**50%**	0.001 ***		0.001 ***		0.033 *	
			**40%**	0.001 ***		0.003 **		0.244	
			**30%**	0.001 ***		0.001 ***		0.005 **	
	**Balanced (50%)**	**All**		0.001 ***		0.003 **		0.001 ***	
		**Subset**		0.001 ***		0.001 ***		0.003 **	
		**Core OTUs**	**60%**	0.138		0.059		0.12	
			**50%**	0.002 **		0.019 *		0.041 *	
			**40%**	0.002 **		0.062		0.132	
			**30%**	0.001 ***		0.005 **		0.008 **	
	**Weighted (100%)**	**All**		0.012 *		0.007 **		0.003 **	
		**Subset**		0.012 *		0.007 **		0.004 **	
		**Core OTUs**	**60%**	0.381		0.063		0.125	
			**50%**	0.008 **		0.217		0.078	
			**40%**	0.023 *		0.467		0.122	
			**30%**	0.021 *		0.014 *		0.06	
**Bray–Curtis**		**All**		0.001 ***		0.001 ***		0.23	
		**Subset**		0.001 ***		0.001 ***		0.273	
		**Core OTUs**	**60%**	0.009 **		0.004 **		0.079	
			**50%**	0.001 ***		0.008 **		0.394	
			**40%**	0.001 ***		0.002 **		0.327	
			**30%**	0.001 ***		0.001 ***		0.388	
**Jaccard**		**All**		0.001 ***		0.001 ***		0.147	
		**Subset**		0.001 ***		0.001 ***		0.161	
		**Core OTUs**	**60%**	0.002 **		0.003 **		0.073	
			**50%**	0.001 ***		0.002 **		0.386	
			**40%**	0.001 ***		0.002 **		0.22	
			**30%**	0.001 ***		0.001 ***		0.254	
				**Salmon**	**SalmoSim**	**Salmon**	**Inoculum**	**SalmoSim**	**Inoculum**
**Number of samples**				18	54	18	9	54	9
**Number of OTUs**				192	508	192	388	508	388
**Number of shared OTUs**				139	139	131	131	291	291
**Number of reads**				78,400	1,004,494	78,400	192,429	1,004,494	192,429
**Shared OTU reads**				77,123	707,199	76,963	134,984	989,884	187,569
**% shared OTU reads**				98.37%	70.40%	98.17%	70.15%	98.55%	97.47%

The table summarises different beta diversity analysis outputs calculated by using different distances: phylogenetic (unweighted, balanced, and weighted UniFrac) and ecological (Bray–Curtis and Jaccard), between different samples (data from all gut compartments combined): real salmon (*Salmon*), SalmoSim inoculum from the real salmon (*Inoculum*), and SalmoSim (only stable time points: 16, 18, and 20 fed on Fishmeal diet and 36, 38, and 40 fed on Fishmeal-free diet). A permutational multivariate analysis of variance (PERMANOVA) by using phylogenetic and ecological distances was performed to determine if the separation of selected groups is significant as a whole and in pairs. Numbers represent *p* values, with *p* values < 0.05 identifying statistically significant differences between compared groups. The comparisons are shown for 3 different datasets: all (completed data set containing all the OTUs sequenced: 978 OTUs in total), subset (containing OTUs that appear only in more than 3 samples and contribute to 99.9% of abundance within each sample: 374 OTUs in total), and core OTUs (containing OTUs that appear in 60% (6 OTUs in total), 50% (13 OTUs in total), 40% (34 OTUs in total) and 30% (65 OTUs in total) of the samples). The asterisks flag the levels of significance: one asterisk (*) for *p* values between 0.05 and 0.01, two asterisks (**) for *p* values between 0.01 and 0.001, and three asterisks (***) for *p* values below 0.001. Finally, the bottom of the table compares number of samples, OTUs, reads for each sample group, as well as number of shared OTUs and their reads within each sample within compared groups. It also summarises what percentage of a given group of samples’ total reads came from the shared OTUs. The SalmoSim samples used for this test consist of stable SalmoSim time points: days 16, 18, and 20 (Fishmeal diet—once bacterial communities adapted to the SalmoSim environment) and days 36, 38, and 40 (Fishmeal-free diet—once bacterial communities adapted to feed change). For non-inoculum real salmon, all samples were included (fed on both Fishmeal and Fishmeal-free diets), and for inoculum real salmon, all samples were included (fed on Fishmeal diet)

Between real salmon that were not the direct source of inocula and SalmoSim, and between salmon used as inocula and other individual salmon, however, statistically significant differences were found in using all metrics regardless of inclusion of rare OTUs. These observations are consistent with inter-individual variation—SalmoSim and inoculum samples originated from the same individuals, while other salmon samples were, by necessity, collected from different individuals during the *in vivo* trial. Furthermore, while the number of OTUs between salmon not used to inoculate (192 OTUs) and inoculum salmon samples (388 OTUs) are different (Table 2), these non-inoculum salmon share 131 OTUs out of 192 OTUs with inoculums, and these 131 OTUs account for around 98% of the total reads. Thus, extra OTUs found only in inoculum salmon and not in others are relatively rare in abundance terms. Differences in OTU numbers and composition are not unexpected as a slightly larger amount of inoculum sample was collected (5 cm of intestine length vs 1 cm for other salmon).

### Effect of changing diet on the microbiome of real salmon (*in vivo*) and SalmoSim (*in vitro*)

#### The impact of diet on the abundance of individual taxa

**Fig. 4:**
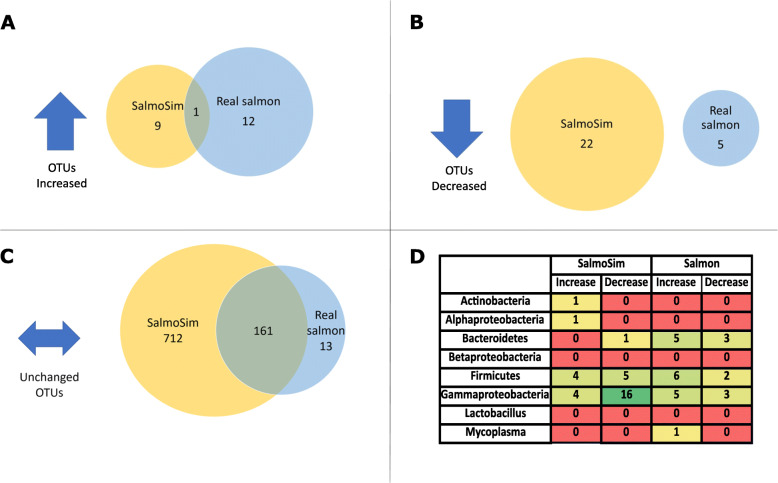
Differential abundance of OTUs within the real salmon and SalmoSim samples fed on Fishmeal and Fishmeal-free diets. ***A*** Venn diagram representing number of OTUs that were upregulated in both SalmoSim and real Salmon samples once the feed was switched. ***B*** Venn diagram representing number of OTUs that were downregulated in both sample after the feed change. ***C*** Venn diagram representing number of OTUs that did not change (relative abundance did not change) within SalmoSim and real salmon samples despite feed switch. ***D*** Table summarising number of OTUs that increased/decreased after feed change in real salmon and SalmoSim samples within different bacterial groups (that same that were analysed by using qPCR approach). Red colour indicates values that are 0, transitioning to greener colours representing higher values

In response to the change of diet, the relative abundances of individual taxa in salmon vs SalmoSim also revealed some differences, as well as multiple similarities in response of the two systems (Fig. 4). In this respect, the abundance of the vast majority of OTUs (SalmoSim: 97%; Salmon: 95%; Fig. 4C) were unaffected by the change in feed; these included 161 OTUs shared by SalmoSim and the real salmon assayed. For OTUs whose individual abundance was impacted by feed across the two systems, only a single common OTU changed in the same way in both Salmon and SalmoSim (Fig. 4A). qPCR-based estimates of taxon abundance variation in response to diet (Supplementary Table 5) and corresponding data for the same taxa from 16S OTU profiles (Fig. 4D) show several similarities and differences between SalmoSim and real salmon. Again, however, the overall pattern is that of limited change in both *in vivo* and *in vitro* systems in response to the change in diet. Invariance observed in the microbiome in response to feed was reflected in estimates of physical attributes of fish in response to the change in feed formulation. As such, no statistically significant differences in various phenotypic measurements (fish length, weight, gutted weight, carcass yield, gonad, and liver weights) were noted in salmon fed on the two different diets used in the experiment (see Supplementary Figure 8). VFA measurements were undertaken to assay any differences between the microbial fermentation profiles of SalmoSim microbes fed on the different diets. Consistent with a limited impact of the two different feeds observed in both *in vivo* and *in vitro* datasets, invariance was also observed in *in vitro* VFA production data, in which no significant differences were observed in SalmoSim between the FMD and FMF diets (see Supplementary Figure 9). Finally, while ammonia production levels were largely static throughout the experiment, protein levels in the system did fluctuate (Figure S5), however, not apparently in response to the change in feed.

#### Microbial composition in SalmoSim and real salmon fed different feeds

**Fig. 5:**
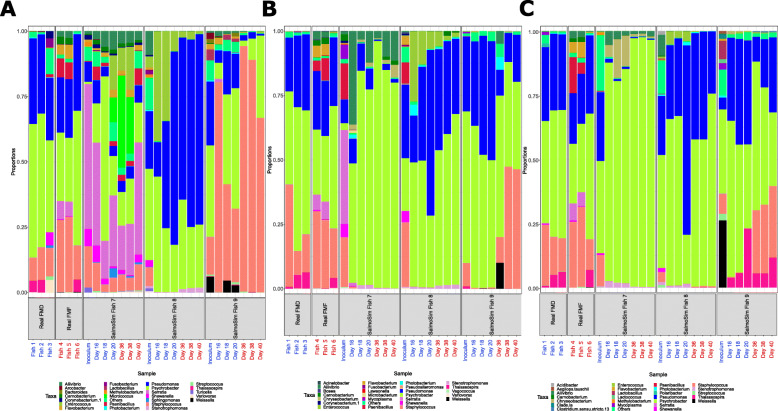
Microbial composition (25 most common genus + others) amongst sample types and feeds. **A** Microbial composition within stomach compartment, **B** Microbial composition within pyloric caeca compartment, and **C** microbial composition within midgut compartment. The different sample types are represented by the labels on the x-axis: Real FMD (real salmon fed on Fishmeal: Fish 1, 2, and 3), Real FMF (real salmon fed on Fishmeal-free diet: Fish 4, 5, and 6), SalmoSim Fish 7–9 (SalmoSim biological replicate runs 1–3). Labels in blue represent samples fed on Fishmeal diet and in red samples fed on Fishmeal-free diet. For SalmoSim, only stable time points for each feed were selected: time points 16–20 for Fishmeal diet, and time points 36–40 for Fishmeal-free diet

Most gut compartments for real salmon, SalmoSim, and the salmon used to inoculate SalmoSim were abundant in *Pseudomonas*, *Psychrobacter*, and *Staphylococcus* genera, suggesting that genera present in the marine phase-salmon are generally maintained in SalmoSim (Fig. 5), with these three genera accounting for 42%, 39%, and 34% of all OTUs in non-inocula salmon, inoculum salmon, and SalmoSim samples, respectively. In terms of change in alpha diversity, the only statistically significant difference in response to the switch in feed was observed in the pyloric caeca compartment within SalmoSim based on the Shannon diversity metric (Supplementary Figure 6), where a slight decrease alongside the FMF occurred. Otherwise, the change in feed formulation did not impact alpha diversity in any gut compartment, either in real salmon or in SalmoSim. Furthermore, no differences were indicated between real salmon and SalmoSim within each gut compartment (Supplementary Figure 7).

**Fig. 6:**
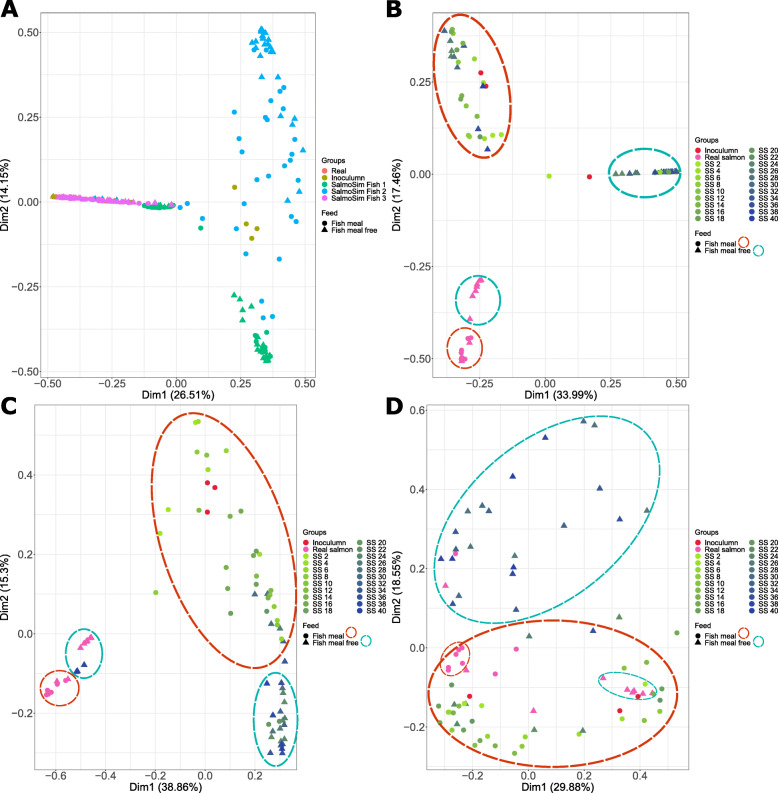
PCoA analysis for various samples fed on different feeds. Figure visualises four Principal Coordinates Analysis (PCoA) plots for Bray–Curtis dissimilarity measures for different samples (inoculum, real salmon, and SalmoSim), different sampling time points from SalmoSim system, different biological replicates, and different feeds. **A** represents all sequenced data together (all real salmon, inoculum, and all 3 biological SalmoSim runs) in which different colours represent different samples (real salmon, inoculum, and 3 different SalmoSim biological replicates (Fish 1, Fish 2, Fish 3)) and different shapes represent different feeds, while **B–D** (subsets of Fig. 6A) represent sequenced data together for real salmon, inoculum, and different biological replicates of SalmoSim (**B** Fish 7, **C** Fish 8, **D** Fish 9). In ***B***–***D***, different colours represent different samples (inoculum, real salmon, and different sampling points of SalmoSim), different shapes represent samples fed on two different feeds, and samples fed on same feeds were circled manually in dotted circles. Dim 1 is principal coordinate 1, and Dim 2 is principle coordinate 2

To provide an overview of microbial composition and variation in the experiment, a PCoA (Principal Coordinates Analysis) based on Bray–Curtis distance was performed and plotted (Fig. 6A–D). The biological replicate (the fish providing the founding inoculum of each SalmoSim run) appears to be a major driver of community composition in the experiment (Fig. 6A). Taxonomic composition represented in stacked bar plots in Fig. 5 also supports this observation. Once individual SalmoSim runs (biological replicates) are visualised separately, changes to microbial communities in response to the feed become apparent (Fig. 6B–D). Statistical comparisons based on PERMANOVA show there is an effect of feed on microbial composition in both salmon and SalmoSim (Table 3); however, based on OTU differential abundance data (above), the effect seems to be small (Fig. 4). Samples from the real salmon fed on the different diets also diverge from one and other (supported by Table 3, Fig. 5), however, not necessarily along the same axes as each SalmoSim replicate. This divergence is potentially indicative of an effect of the biological replicate (i.e., inter-individual variation). Consistent with Fig. 5, inoculum for the respective SalmoSim replicates cluster among SalmoSim samples for the FMD in each case.

**Table 3:** Beta diversity analysis for various samples fed on different feeds

		Fishmeal vs Fishmeal-free diets		
		Salmon	SalmoSim	Stable SalmoSim
**UniFrac**	**Unweighted (0%)**	0.001 ***	0.002 **	0.062
	**Generalised (50%)**	0.001 ***	0.001 ***	0.251
	**Weighted (100%)**	0.016 *	0.011 *	0.288
**Bray–Curtis**		0.008 **	0.001 ***	0.126
**Jaccard**		0.01 **	0.001 ***	0.053
**Number of differentially abundant OTUs**		18	32	28

Table summarises different beta diversity analysis outputs calculated by using different distances*—*phylogenetic (unweighted, balanced, and weighted UniFrac) and ecological (Bray*–*Curtis and Jaccard)*—*between samples fed on Fishmeal or Fishmeal*-*free diets. Numbers represent p values, with p values < 0.05 identifying statistically significant differences between compared groups. The comparisons are shown for three different subset datasets: Salmon (containing sequenced samples from real salmon), All SalmoSim (containing all samples from SalmoSim system excluding inoculum), and Stable SalmoSim (containing samples only from stable time points: 16, 18*,* and 20 fed on Fishmeal (once bacterial communities adapted to SalmoSim system), and 36, 38*,* and 40 fed on Fishmeal*-*free diet (once bacterial communities adapted to feed change). The *asterisk*s flag the levels of significance: one *asterisk* (*) for p values between 0.05 and 0.01, two *asterisk*s (**) for p values between 0.01 and 0.001, and three *asterisk*s (***) for p values below 0.001

## Discussion

Our findings suggest a loss of microbial taxa diversity and richness as a result of transferring initial inoculums from real salmon into the SalmoSim system in the pyloric caeca and midgut compartments. Several lines of evidence from our core OTU analysis suggest that low prevalence (rare) OTUs make up most of the taxa lost, and progressive removal of rare OTUs increased the compositional similarity between inoculum and SalmoSim samples using both phylogenetic and ecological distances. Furthermore, shared OTUs between inoculum salmon and SalmoSim samples accounted for around 97% and 98% of total reads respectively, and as such, the microbiota in the model are highly representative of those from the fish that founded them. A general trend was observed, in which all gut compartments became increasingly stable throughout the 40-day experiment, with little-observed impact of introducing the different feed at day 20. Comparison of non-inoculum salmon and SalmoSim samples at the microbial level showed significant differences using both ecological and phylogenetic metrics as well as a different number of OTUs (SalmoSim 508 OTUs and real salmon samples 192 OTUs). These differences may be explained by the fact that samples used for non-inoculum salmon and SalmoSim originated from different individuals, whereas initial inoculum and SalmoSim samples for a given run originated from the same fish. Furthermore, the non-inoculum salmon and inoculum samples were derived slightly differently—with a longer section of the gut sampled for the inoculum samples, which could explain the higher diversity of bacteria (number of OTUs) which in turn can affect beta diversity output. However, despite this, shared OTUs between non-inoculum salmon and inoculum samples accounted for around 98% of total reads generated in non-inoculum real salmon and around 70% within inoculum real salmon samples. Correspondingly, we observed that the biological replicate (the founding inoculum of each SalmoSim run that originated from different individuals) was the major driver of community composition in the experiment. Once the individual runs were separated, phylogenetic and ecological distances suggested that changing feed was a statistically significant driver of community composition in both real salmon and SalmoSim. However, the vast majority of OTUs remained unchanged by the switch in feed in both systems, and no changes were noticeable in the bacterial activity (VFA production) within the system after the introduction of the plant-based feed, nor in phenotypic performance of Atlantic salmon fed on two different feeds (fish length, weight, gutted weight, carcass yield, gonad, and liver weights) in *in vivo* trial.

Many of the microbes we detected and cultured from the salmon gut microbiome have been reported previously in this species. For example, gram-negative *Pseudomonas* and *Psychrobacter*, the most abundant genera we observed, are among the core bacterial taxa known to reside within the real salmon gut [38, 60, 61]. *Staphylococcus* genera have also been reported widely in freshwater- and marine-farmed salmon [62]. SalmoSim was able to maintain these species in culture throughout the experimental run, and although some diversity was lost, no statistical differences could be detected between the composition of SalmoSim and that of the fish gut communities used to found the different biological replicates via ecological metrics. Notable by their scarcity were mycoplasma OTUs, which occurred at relatively low abundance in both the *in vivo* and *in vitro* systems in this study. *Mycoplasma* OTUs were recovered from most SalmoSim gut compartments at low abundances (see supplementary Table 5), suggesting that these fastidious microbes can survive in the bioreactors. Our group and several others have widely reported *Mycoplasma* species from marine and freshwater stage of wild and farmed Atlantic salmon (higher abundance in farmed salmon), where many proliferate intracellularly in the gut epithelial lining [33, 63–65]. Establishing whether mycoplasma can actively proliferate in SalmoSim would require the use of founding communities rich in these organisms in a future experiment. One means of achieving this could be by using mock communities to better understand the temporal development of the observed microbial compositions from the inocula to the SalmoSim community [66].

We identified that a change in feed resulted in an overall shift in microbial community structure in both real salmon and SalmoSim system, as has been found to be the case in many previous studies [10, 67, 68], but the vast majority of OTUs within both real salmon and SalmoSim were not affected by the feed change. The direction of this shift, and the microbial taxa involved, were not equivalent in SalmoSim and real salmon, although no overall trend was observed at higher taxonomic levels in either system. Importantly, it is also the case that the vast majority of OTUs within both real salmon and SalmoSim were not affected by the switch in feed. Furthermore, it was found that change in feed did not affect VFA production in the SalmoSim system. As such, it is not clear whether any relevant functional shifts occurred in the microbiome of SalmoSim or real salmon as a result of the treatment. Furthermore, we did not identify any phenotypic changes (fish length, weight, gutted weight, carcass yield, gonad, and liver weights) within *in vivo* trial of Atlantic salmon fed on two different feeds. This lack of change is not unexpected, considering the plant-based feed was developed to have similar macronutrient composition to a Fishmeal-based feed. One difference is a slightly higher crude fibre (fermentable substrate) proportion in Fishmeal-free diet, which could explain higher microbial diversity in *in vivo* samples fed on plant-based feed [10].

The use of *in vitro* systems to study and model the microbial communities of monogastric vertebrates is becoming increasingly widespread, with systems simulating *Sus scrofa* (pig) [69], *Gallus gallus* (chicken) [70], *Canis lupus* (dog) [71], and other vertebrate guts. Using *in vitro* gut simulators is also a widely accepted approach to study the human gut microbiome [18, 72, 73]. One of the most established systems is the Simulator of the Human Intestinal Microbial Ecosystem (SHIME) that mimics the entire gastrointestinal tract incorporating the stomach, small intestine, and different colon regions [19]. This system was used to study the effects of many different dietary additives on the human microbiome [74, 75]. The value of *in vitro* simulators in providing genuine insights is limited only by the research question and the corresponding level of sophistication required. The host component of the system, for example, is often poorly modelled, although cell lines, artificial mucosae, and digestion / absorbance systems can be included, which can provide specific physiological and metabolic insights [72, 76]. For instance, the growth medium in vessels representing stomach and small intestine of the SHIME system is enriched with pancreatic enzymes and bile, while a further upgrade of SHIME incorporates the mucosal environment allowing a portion of the microbiota present in the vessels to adhere to a gut mucus layer [18, 76]. Furthermore, more sophisticated *in vitro* gut models, such as TIM-2 (the TNO computer-controlled, dynamic *in vitro* gastroIntestinal Model of the colon), incorporates a dialysis system, which helps to maintain a physiological concentration of metabolites and prevents inhibition of the microbial growth by microbial metabolite accumulates [77, 78]. Finally, microfluid-based *in vitro* systems, such as HuMiX, allow coculturing of the host gut cells and microbial cells under conditions and processes mimicking gastrointestinal host-microbiome interface [79].

Whilst studies have been performed using a simple *in vitro* batch method to study fermentation of various feeds using gut inocula from Nile tilapia (*Oreochromis niloticus*) and European sea bass (*Dicentrarchus labrax*), these systems, to date, lack complexity [80]. In this paper, we identified that in order to design and build an artificial gut simulator, parameters including physiochemical conditions (pH, temperature, dissolved oxygen), feed media composition, and flow rate between different gut compartments need to be tightly controlled. If all these parameters can be determined and maintained, and there is access to crude enzyme extracts from relevant species, adaptation of the SalmoSim model to other fish species (e.g., trout, carp) should be possible alongside suitable validation. As we found, interindividual variability may be an important consideration, and adequate biological replication is necessary to enable reliable interpretation of results, a consideration that can be overlooked by even the most sophisticated systems. Interindividual variation in gut microbial communities is widely observed in human studies that demonstrate more between-person variation than within-person variation with adults having an average unique microbial signature that is largely stable over time [81–85]. This is also true in Atlantic salmon—our previous work clearly shows high levels of interindividual variability in farmed [86] and wild [87] fish, as does the work of all others. It was reported, for example, that a single *Lactobacillales* OTU represented 96% of the microbiome of one fish which compared with a mean of only 3.5% relative abundance in the other fish from the same shoal in an aquaculture setting [88]. Well-established gut microbiome systems, such as SHIME, use inoculums from only one donor [18] or in recent studies 2 donors in which differences were found in microbiota distribution even when using control diets [89]. Furthermore, some artificial gut systems pool biological replicates together to produce a “representative microbiome inoculum”, such as in a recent *in vitro* chicken gut model, and even in these systems, microbiomes still show variability [70]. To our knowledge in this study, we are the first to run a gut microbiome model in biological triplicate and to highlight the importance of accounting for interindividual differences before drawing conclusions. Prior to the current study, only one other attempt was made to study the effect of diet on Atlantic salmon gut microbial composition *in vitro* [90]. In this preliminary study, a simple *in vitro* system was used to assess the impact of different feed formulations on the microbial communities of faecal slurries prepared from live salmon. However, no direct comparison was made with a true *in vivo* trial, nor were the different gut compartments present in salmon modelled in any detail and the predictive value for such simple *in vitro* systems in not immediately clear. Nonetheless, the work provided an important catalyst for the development of more sophisticated systems.

In the future, SalmoSim could be improved even further by introducing the ability to assess the digestibility of various feeds. In the aquaculture industry, determination of the digestibility of nutrients in various feeds provides the indication of their nutritional value and is often considered as the first step in feed quality evaluation [91–94]. While some *in vitro* systems used to assess digestibility in Atlantic salmon use, enzymes sourced from pigs, and bovines, such as porcine pepsin/porcine trypsin, bovine chymotrypsin, and porcine peptidase [95], enzyme extracts from different parts of the digestive system from salmon should be chosen to ensure accurate simulation of the *in vivo* digestion process. A modified SalmoSim could include the addition of enzyme extracts from the stomach and pyloric caecum compartments of real salmon in order to catabolise ingredients within the feed being tested [96], as well as suitable system for continuously removing the small molecular products of digestion [97–99].

## Conclusions

Our results indicate that SalmoSim can not only stably maintain the most abundant microbial communities from real salmon, but also demonstrate similar responses to experimental feed treatments as those seen in real salmon. These results are encouraging, however, the nature of the treatment applied in this study—a switch between two similar feeds that had little effect on the gut microbiota *in vivo—*suggests that further experimentation with SalmoSim would be beneficial. For example, the survival and influence of probiotics within the system or the influence of known prebiotics could also be assessed, as has been previously studied in other *in vitro* gut systems [71]. Gut models such as SalmoSim could provide a powerful tool for aquaculture, where there is considerable interest associated with the development of feed and feed additives [100–102], but where the capacity for *in vivo* trials is limited. The steps of the system development described in this paper can be used as guidelines to develop various other systems representing other fish species. The aim of such systems could be to provide prescreening tool for new feed ingredients and additives with the aim of reducing the cost and scale of *in vivo* testing. In parallel, an *in vitro* gut model for salmon could also be exploited to understand questions of public health importance (e.g., antimicrobial resistance and transfer [70]), as well as the fundamental ecological processes that underpin microbiome dynamics and assembly.

## Supplementary Information

**Additional file 1.** Supplementary methods.

**Additional file 2: Table S1.** 16S rRNA gene-targeted group-specific primers. Table lists primer sets that already published and validated in the literature. All primers were used on mouse faeces samples apart from Alphaproteobacterial specific primers that were used on marine biofilm samples.

**Additional file 3: Table S2.** First round PCR primers used for the first round of NGS library preparation.

**Additional file 4.** Table S3 Second round PCR primers used for the first round of NGS library preparation.

**Additional file 5: Table S4.** OTUs prevalence analysis by sub-setting full dataset into multiple core OTUs. The table summarises the number OTUs within each subset dataset (subset by the % of samples that share OTUs) and percentage of the total number of OTUs within the full dataset (100%). This table also shows the number of reads and the percentage of total reads (100%) within each of the subset datasets. Note: in the 60% subset three samples were lost as they did not retain any under that criteria OTUs: Id-val1-PC1, Id-Val2-MG4 and Id-Val2-PC1.

**Additional file 6: Table S5.** Bacterial group responses to feed change within different gut compartments in real salmon and SalmoSim based on qPCR data. The table summarises the Estimated Marginal Means output for each mixed-effect linear model run with different qPCR measured relative abundance values identifying the difference between real salmon and SalmoSim response to feed change (Fish meal to Fish meal free diet) within different gut compartments (S – stomach, PC – pyloric caeca, and MG – mid gut). P>0.05 values identify no change in the bacterial group, p<0.05 identifies decrease (Est is negative), and p<0.05 identifies increase (Est is positive) in the relative abundance of target group after the feed change. Bold values identify similarities between SalmoSim and real salmon samples. The SalmoSim values used for this test involves stable SalmoSim time points: days 16, 18 and 20 (Fish meal diet – once bacterial communities adapted to the SalmoSim environment), and days 36, 38 and 40 (Fish meal free diet – once bacterial communities adapted to feed change).

**Additional file 7: Figure S1.** Physiochemical conditions measured within different real Atlantic salmon gut compartments. 1A-1C measured physicochemical conditions within real salmon (n=3) gut compartments: pH (1A), temperature (°C, 1B), dissolved oxygen (mg/L, 1C).

**Additional file 8: Figure S2.** Specificity of the primers that target *Lactobacillus* and *Mycoplasma* genus. The results in figure summarise bacterial genus targeted by *Lactobacillus* (Fig. 1 A, B, C) and *Mycoplasma* (Fig. 1 D, E, F) specific primer set. It shows that of all genus captured by *Lactobacillus* primer pair 98% were *Lactobacillus* in fish 1, 78% in fish 2, and 65% in fish 3. While of all genus captured by *Mycoplasma* primer pair 95% were *Mycoplasma* in fish 1, 79% in fish 2, and 56% in fish 3.

**Additional file 9: Figure S3.** Stability within SalmoSim system calculated by using unweighted and weighted UniFrac values for pairwise beta diversity analysis. The figure represents microbial stability within the SalmoSim system (data from all gut compartments combined) as the pairwise beta diversity comparison between different sampling time points (days), calculated by using A unweighted (0%) and B weighted (100%) UniFrac as a distance measure. A small p-value indicates that the two time points are statistically different, and p>0.05 indicates that two time points are not statistically different. The colour key illustrates the p-value: red end of spectrum denoting low p values (distinct compositions between time points) and dark green indicating high p values (similar compositions between timepoints).

**Additional file 10: Figure S4.** Measured value (qPCR, ammonia and protein concentrations) stability within different SalmoSim compartments fed on Fish meal and Fish meal free diets. The figure summarises the Estimated Marginal Means output for each mixed-effect linear model (Model 1) run with different values measured in different SalmoSim compartments (qPCR measurements, ammonia and protein concentrations) identifying the difference between different time points during the first (system fed on Fish meal diet) and last 20 days (system fed on Fish meal free diet) of validation experiment. A small p-value indicates that the two time points are statistically different, and p>0.05 indicates that two time points are not statistically different. The colour key illustrates the p-value: red end of spectrum denoting low p values (low correlation between time points) and dark green indicating high p values (no differences between timepoints).

**Additional file 11: Figure S5.** Stability within SalmoSim system, within different biological replicates and different gut compartments, calculated by using generalised UniFrac values for pairwise beta diversity analysis. The figure represents microbial stability within the SalmoSim system (data separated by different biological replicates and gut compartments) as the pairwise beta diversity comparison between different sampling time points (days), calculated by using generalised (50%) UniFrac as a distance measure. A small p-value indicates that the two time points are statistically different, and p>0.05 indicates that two time points are not statistically different. The colour key illustrates the p-value: red end of spectrum denoting low p values (distinct compositions between time points) and dark green indicating high p values (similar compositions between timepoints).

**Additional file 12: Figure S6.** Stability within SalmoSim system calculated by using different UniFrac values for pairwise beta diversity analysis. The figure represents microbial stability within the SalmoSim system (data from all gut compartments and two different technical replicate runs combined) as the pairwise beta diversity comparison between different sampling time points (days), calculated by using unweighted (0%), generalised (50%) and weighted (100%) UniFrac as a distance measure. A small p-value indicates that the two time points are statistically different, and p>0.05 indicates that two time points are not statistically different. The colour key illustrates the p-value: red end of spectrum denoting low p values (distinct compositions between time points) and dark green indicating high p values (similar compositions between timepoints).

**Additional file 13: Figure S7.** Calculated alpha-diversity metrics within different gut compartments of real salmon and SalmoSim fed on Fish meal and Fish meal free diets. Figure visually represents different alpha diversity outputs within different gut compartments of real salmon in red and SalmoSim in yellow (stable time points: 16, 18 and 20 fed on Fish meal, and 36, 38 and 40 fed on Fish meal free diet) fed on Fish meal and Fish meal free diets. A visually represents effective richness (number of OTUs), B represents effective Shannon diversity. The lines above bar plots represent statistically significant differences after feed change. The stars flag the levels of significance: one star (*) for p-values between 0.05 and 0.01, two stars (**) for p-values between 0.01 and 0.001, and three stars (***) for p-values below 0.001.

**Additional file 14: Figure S8.***In vivo* phenotypic fish performance fed on two different feeds. Figure visually represents different phenotypic performance data of fish (n=32 per feed) fed on two different feed. A Atlantic salmon length in centimetres; B Atlantic salmon length in weight in kilograms; C Atlantic salmon percentage carcass yield; D Atlantic salmon gonad weight in grams; E Atlantic salmon gutted weight in kilograms; F Atlantic salmon liver weight in grams. Blue box plots represent data from salmon (n=32) fed on Fish meal free diet, and red represents Atlantic salmon fed on Fish meal diet (n=32).

**Additional file 15: Figure S9.** VFA production within different SalmoSim compartments fed on different feeds. Figure represents 11 volatile fatty acid production within SalmoSim system fed on Fish meal and Fish meal free diets within different gut compartments. Y axis represents the concentration of specific volatile fatty acid (mM) while the X axis represents each gut compartment (stomach, pyloric caeca, midgut). Red colour denoted Fish meal and blue – Fish meal free diets. The lines above bar plots represent statistically significant differences between different feeds and gut compartments. The stars flag the levels of significance: one star (*) for p-values between 0.05 and 0.01, two stars (**) for p-values between 0.01 and 0.001, and three stars (***) for p-values below 0.001.
