# Supplementary material for: SalmoSim: the development of a three-compartment in vitro simulator of the Atlantic salmon GI tract and associated microbial communities
Source: Microbiome. 2021 Aug 31;9:179. doi: 10.1186/s40168-021-01134-6 (PMC8408954; doi:10.1186/s40168-021-01134-6)
Supplement: Supplementary file 2 — Additional file 1. Supplementary methods. [file 40168_2021_1134_MOESM2_ESM.docx]

**Supplementary methods**

1. **qPCR data analysis**
   1. **Performing qPCR analysis**

The concentration of each DNA sample was measured using a Qubit® fluorometer (Thermo Fisher Scientific, USA), and dilutions were performed using Microbial DNA-Free Water (Qiagen, Valencia, CA, USA). Inoculums from all three real salmon gut compartments were diluted to 0.25 ng/µl. SalmoSim stomach samples were also diluted to 0.25 ng/µl, and pyloric caecum and midgut SalmoSim samples were diluted to 1 ng/µl. After, the qPCR analysis was performed on each DNA sample in duplicates by using SensiFAST™ SYBR® No-ROX Kit (Bioline, UK) and primer sets summarised in Supplementary Table 1 at a final concentration of 1 pM of each primer. Reaction conditions for all PCR reactions were 95°C for three minutes, followed by 40 cycles at 95°C for 5 seconds, 60°C for 10 seconds and 72°C for 20 seconds, followed by a final elongation step of 95°C for 10 minutes.

In order to measure the relative abundance of the target group (target determined by the specificity of the qPCR primer pairs); several steps were undertaken by adapting ∆∆Cq method (Rao et al., 2013). First, the average quantitation cycle (Cq) value of each primer set negative control was found. This was followed by subtraction Cq value generated by using one of the primer pairs in Supplementary Table 1, from corresponding average Cq value of the corresponding negative control (generated with the same primer pair) in order to generate value X. After, the Cq value generated by using the general primer set was subtracted from the average Cq value of the corresponding negative control (generated using general primer set) in order to generate the value Y. Finally, the value X was divided by the value Y in order to find out the relative abundance of the target group with respect to the total number of bacterial 16S copies in the sample. The equations used for all these calculations are summarised below:

$$x=average negative control Cq-Cq of target group$$

$$y=average of negative control Cq-Cq of general bacteria$$

$$relative abundance of target group= x \div y$$

This method was carried out for each sample, quantified using different primer sets targeting various bacterial taxon groups. Several published and validated primer sets in the literature were used (Supplementary Table 1). Primer sets targeting Mycoplasma, and Lactobacillus genus were designed by using DECIPHER software based on the data collected by Heys et al., 2020. These primers target specificity was analysed via amplicon sequencing of the products (See Supplementary Figure 2).

- 1. **Investigating bacterial dynamics within SalmoSim system over time**

In order to investigate the time taken for the measured values (qPCR of different bacterial groups, protein and ammonia concentrations) to stabilise within different gut compartments of the SalmoSim system, the data for all three SalmoSim runs (three biological replicates) was combined and then split-up by each separate SalmoSim compartment (stomach, pyloric caecum and midgut). These three resulting datasets were then further split-up in half: pre- and post-feed changes. The subdivided datasets by different SalmoSim gut compartment and different feed were used to run Model 1 with different values measured during a validation experiment considering run (biological replicates) as a random effect. The normality and heterogeneity of the residuals for of each model were checked by using Shapiro and Bartlett’s tests. If these tests showed that residues were not normally distributed or not heterogeneous, the data were subsequently transformed by using Code 1 and then used to re-run Model 1. Finally, the post-hoc Estimated Marginal Means test (also known as Least-Squares Means) was used with Model 1 and ran with different values in order to investigate in-between individual time point comparison.

Model 1 = lme(value~ Time, random =c(~1|Run), data=**groupX**)

**Model 1 Mixed effect linear model formula to investigate the effect of time taking run as a random effect.** In Model 1 groupX denotes subdivided data sets by different SalmoSim compartments (stomach, pyloric caecum or midgut), and value denotes the qPCR results for one of the different targeted bacterial groups (Actinobacteria, Alphaproteobacteria, Bacteroidetes, Betaproteobacteria, Firmicutes, Gammaproteobacteria, Lactobacillus, Mycoplasma) or protein or ammonia concentrations. This model takes different SalmoSim runs as a random effect (biological replicates).

if(lambda!=0){y=((value)^lambda-1)/lambda}

if(lambda==0){y=log(value)}

**Code 1 Code used to transform the data.** Value in this code denotes the qPCR results for one of the different targeted bacterial groups (Actinobacteria, Alphaproteobacteria, Bacteroidetes, Betaproteobacteria, Firmicutes, Gammaproteobacteria, Lactobacillus, Mycoplasma) or protein or ammonia concentrations. Lambda values in 0.1 increments were tested.

- 1. **Comparing *in vivo* and *in vitro* trials**

In order to investigate whether a change in the feed from FMD to FMF results in similar trends measured between SalmoSim and real salmon samples, a combined data set was produced containing qPCR values measured in real salmon gut compartments (stomach, pyloric caecum and midgut of three fish fed on FMD and three fish fed on FMF) and SalmoSim compartments at the last three time points for both feeds (once bacterial communities were stabilised while feeding SalmoSim both FMF and FMD: days 16, 18, and 20 for FMD and days 36, 38, and 40 for FMF feed). This combined dataset was then separated by different SalmoSim gut compartments (stomach, pyloric caecum, and midgut). The subdivided datasets for each gut compartment for both real salmon (all samples) and SalmoSim (samples from only stable time points) were input into Model 2. In order to investigate how bacterial groups within different types of sample (SalmoSim or real salmon) react to the change in feed the Post-hoc Estimated Marginal Means test (also known as Least-Squares Means) was used in order to have a more detailed look at the effect of the interaction between feed and sample on the abundance of each target taxon.

Model 2 = lm(value~ Feed*samples, data=GroupX)

**Model 2 Linear model formula to investigate the effect of interaction between feed (Fish meal and Fish meal free diets) and sample (real salmon and SalmoSim samples) on the qPCR values measured.** In model 2, value denotes the qPCR results for one of the different targeted bacterial groups (Actinobacteria, Alphaproteobacteria, Bacteroidetes, Betaproteobacteria, Firmicutes, Gammaproteobacteria, Lactobacillus, Mycoplasma). Group X is a subset of dataset separated by different gut compartments (stomach, pyloric caecum and midgut). Feed identifies Fish meal and Fish meal 0 diets, and sample identifies real salmon and stable time point SalmoSim samples (days 16, 18 and 20 SalmoSim fed on Fish meal diet, and days 36, 38, and 40 SalmoSim fed on Fish meal free diet).

1. **Volatile Fatty Acid (VFA) analysis**

The measured VFA values were input to Model 4 including time point (sampling time point) and run (biological replicate of SalmoSim system) as random effects. This was followed by the Post-hoc test Estimated Marginal Means (also known as Least-Squares Means) in order to have a more detailed look at the effect of the interaction between feed and SalmoSim compartment affect the concentration of VFAs.

**Model 3 = lmer(VFA~ Feed*Compartment+(1|Time point)+(1|Run))**

**Model 3 Mixed effect linear model to investigate the significance of different VFA concentrations between SalmoSim fed on Fish meal and Fish meal free diets within different SalmoSim compartments.** In Model 3 VFA denotes the VFA values measured. This model takes run and time as random effects.

1. **Beta-diversity analysis workflow**

#' **Script**: Beta-Diversity analysis for SalmoSim

#' **Author**: Raminta Kazlauskaite (adapted from Rhea script produced by Ilias Lagkouvardos)

#' Calculate beta-diversity for microbial communities

#' based on permutational mulitvariate analysis of variances (PERMANOVA) using multiple distance matrices

#' computed from phylogenetic distances between observed organisms

#'

#' **Input**:

#' 1. Set the path to the directory where the file is stored

#' 2. Write the name of the normalized OTU table without taxonomy information

#' 3. Write the name of the mapping file that includes the samples groups

#' 4. Write the name of the OTU tree

#' 5. Write the name of the variable (sample group) used for comparison

#'

#'

#' **Output**:

#' The script generates three graphical outputs (pdf), one text file and a newick tree

#' 1. A phylogram with colour-coded group clustering

#' 2. MDS and NMDS plots showing information about beta-diversity across all sample groups

#' 3. MDS and NMDS plots of all pairwise comparisons

#' 4. The distance matrix

#' 5. Plot showing the optimal number of clusters

#' 6. Dendogram for all samples in a newick tree file

#'

#' **Concept**:

#' A distance matrix is calculated based on the generalized UniFrac approach

#' (Chen J, et al. Associating microbiome composition with environmental covariates using generalized UniFrac distances. 2012)

#' Samples are clustered based on the distance matrix using the Ward's hierarchical clustering method

#' To determine similarities between samples, a multivariate analysis is applied

#' and sample distribution is illustrated by means of MDS and NMDS (non-metric) plots

#' The Calinski-Harabasz (CH) Index is used to assess the optimal number of clusters the dataset was most robustly partitioned into

##################################################################################

**###### Set parameters in this section manually ######**

##################################################################################

#' Please set the directory of the script as the working folder (e.g D:/studyname/NGS-Data/Rhea/beta-diversity/)

#' Note: the path is denoted by forward slash "/"

setwd("") #<--- CHANGE ACCORDINGLY

#' Please give the file name of the normalized OTU-table without taxonomic classification

input_otu = "" #<--- CHANGE ACCORDINGLY !!!

#' Please give the name of the meta-file that contains individual sample information

input_meta = "" #<--- CHANGE ACCORDINGLY !!!

#' Please give the name of the phylogenetic tree constructed from the OTU sequences

input_tree = "" #<--- CHANGE ACCORDINGLY !!!

#' Please give the column name (in the mapping file) of the categorical variable to be used for comparison (e.g. Genotype)

group_name = "" #<--- CHANGE ACCORDINGLY !!!

##################################################################################

**###### Additional parameters ######**

##################################################################################

#' Turn on sample labeling

#' 0 = Samples are not labeled in the MDS/NMDS plots

#' 1 = All Samples are labed in the MDS/NMDS plots

label_samples = 0

#' Determine which sample lable should appear

#' Write the name of samples (in quotation marks), which should appear in the MDS/NMDS plots, in the vector (c) below

#' If more than one sample should be plotted, please separate their IDs by comma (e.g. c("sample1","sample2"))

label_id =c("")

#' De-Novo Clustering will be perfomed for the number of samples or maximal for the set limit

#' Default Limit is 100

kmers_limit=20

###### NO CHANGES ARE NEEDED BELOW THIS LINE ######

##################################################################################

**###### Main Script ######**

##################################################################################

################### **Load all required libraries** ########################

# Check if required packages are already installed, and install if missing

packages <-c("ade4","GUniFrac","phangorn","cluster","fpc")

# Function to check whether the package is installed

InsPack <- function(pack)

{

if ((pack %in% installed.packages()) == FALSE) {

install.packages(pack,repos ="http://cloud.r-project.org/")

}

}

# Applying the installation on the list of packages

lapply(packages, InsPack)

# Make the libraries

lib <- lapply(packages, require, character.only = TRUE)

# Check if it was possible to install all required libraries

flag <- all(as.logical(lib))

################### **Read all required input files** ####################

# Load the tab-delimited file containing the values to be analyzed (samples names in the first column)

otu_file <- read.table (file = input_otu, check.names = FALSE, header = TRUE, dec = ".", sep = "\t", row.names = 1, comment.char = "")

# Clean table from empty lines

otu_file <- otu_file[!apply(is.na(otu_file) | otu_file =="",1,all),]

# Load the mapping file containing individual sample information (sample names in the first column)

meta_file <- read.table (file = input_meta, check.names = FALSE, header = TRUE, dec = ".", sep = "\t", row.names = 1, comment.char = "")

# Clean table from empty lines

meta_file <- data.frame(meta_file[!apply(is.na(meta_file) | meta_file=="",1,all),])

# Load the phylogenetic tree calculated from the OTU sequences

tree_file <- read.tree(input_tree)

# Create the directory where all output files are saved (is named after the target group name set above for comparisons)

dir.create(group_name)

#################### **Calculate beta-diversity** ###################

# OTU-table and mapping file should have the same order and number of sample names

# Order the OTU-table by sample names (ascending)

otu_file <- otu_file[,order(names(otu_file))]

# Transpose OTU-table and convert format to a data frame

otu_file <- data.frame(t(otu_file))

# Order the mapping file by sample names (ascending)

meta_file <- data.frame(meta_file[order(row.names(meta_file)),])

# Save the position of the target group name in the mapping file

meta_file_pos <- which(colnames(meta_file) == group_name)

# Select metadata group based on the pre-set group name

all_groups <- as.factor(meta_file[,meta_file_pos])

# Root the OTU tree at midpoint

rooted_tree <- midpoint(tree_file)

# Calculate the UniFrac distance matrix for comparing microbial communities: 0.0 – Unweighted UniFrac, 0.5 Balanced UniFrac and 1.0 Weighted UniFrac.

unifracs <- GUniFrac(otu_file, rooted_tree, alpha = c(0.0,0.5,1.0))$unifracs

# Weight on abundant lineages so the distance is not dominated by highly abundant lineages with 0.5 having the best power

unifract_dist <- unifracs[, , "d_0.5"]

################ **Generate tree** #######################

# Save the UniFrac output as distance object

all_dist_matrix <- as.dist(unifract_dist)

# Apply a hierarchical cluster analysis on the distance matrix based on the Ward's method

all_fit <- hclust(all_dist_matrix, method = "ward.D2")

# Generates a tree from the hierarchically generated object

tree <- as.phylo(all_fit)

my_tree_file_name <- paste(group_name,"/phylogram.pdf",sep="")

plot_color<-rainbow(length(levels(all_groups)))[all_groups]

# Save the generated phylogram in a pdf file

pdf(my_tree_file_name)

# The tree is visualized as a Phylogram color-coded by the selected group name

plot(tree, type = "phylogram",use.edge.length = TRUE, tip.color = (plot_color), label.offset = 0.01)

print.phylo(tree)

axisPhylo()

tiplabels(pch = 16, col = plot_color)

dev.off()

################# **Build NMDS plot**  ########################

# Generated figures are saved in a pdf file

file_name <- paste(group_name,"beta-diversity.pdf",sep="_")

pdf(paste(group_name,"/",file_name,sep=""))

# Calculate the significance of variance to compare multivariate sample means (including two or more dependent variables)

# Omit cases where there isn't data for the sample (NA)

all_groups_comp <- all_groups[!is.na(all_groups)]

unifract_dist_comp <- unifract_dist[!is.na(all_groups), !is.na(all_groups)]

adonis<-adonis(as.dist(unifract_dist_comp) ~ all_groups_comp)

all_groups_comp<-factor(all_groups_comp,levels(all_groups_comp)[unique(all_groups_comp)])

# Calculate and display the MDS plot (Multidimensional Scaling plot)

s.class(

cmdscale(unifract_dist_comp, k = 2), col = unique(plot_color), cpoint =

2, fac = all_groups_comp, sub = paste("MDS plot of Microbial Profiles\n(p-value ",adonis[[1]][6][[1]][1],")",sep="")

)

if (label_samples==1) {

lab_samples <- row.names(cmdscale(unifract_dist_comp, k = 2))

ifelse (label_id != "",lab_samples <- replace(lab_samples, !(lab_samples %in% label_id), ""), lab_samples)

text(cmdscale(unifract_dist_comp, k = 2),labels=lab_samples,cex=0.7,adj=c(-.1,-.8))

}

# Calculate and display the NMDS plot (Non-metric Multidimensional Scaling plot)

meta <- metaMDS(unifract_dist_comp,k = 2)

s.class(

meta$points, col = unique(plot_color), cpoint = 2, fac = all_groups_comp,

sub = paste("metaNMDS plot of Microbial Profiles\n(p-value ",adonis[[1]][6][[1]][1],")",sep="")

)

if (label_samples==1){

lab_samples <- row.names(meta$points)

ifelse (label_id != "",lab_samples <- replace(lab_samples, !(lab_samples %in% label_id), ""), lab_samples)

text(meta$points,labels=lab_samples,cex=0.7,adj=c(-.1,-.8))

}

#close the pdf file

dev.off()

############### **NMDS for pairwise analysis** ###################

# This plot is only generated if there are more than two groups included in the comparison

# Calculate the pairwise significance of variance for group pairs

# Get all groups contained in the mapping file

unique_groups <- levels(all_groups_comp)

if (dim(table(unique_groups)) > 2) {

# Initialise vector and lists

pVal = NULL

pairedMatrixList <- list(NULL)

pair_1_list <- NULL

pair_2_list <- NULL

for (i in 1:length(combn(unique_groups,2)[1,])) {

# Combine all possible pairs of groups

pair_1 <- combn(unique_groups,2)[1,i]

pair_2 <- combn(unique_groups,2)[2,i]

# Save pairs information in a vector

pair_1_list[i] <- pair_1

pair_2_list[i] <- pair_2

# Generate a subset of all samples within the mapping file related to one of the two groups

inc_groups <-

rownames(subset(meta_file, meta_file[,meta_file_pos] == pair_1

|

meta_file[,meta_file_pos] == pair_2))

# Convert UniFrac distance matrix to data frame

paired_dist <- as.data.frame(unifract_dist_comp)

# Save all row names of the mapping file

row_names <- rownames(paired_dist)

# Add row names to the distance matrix

paired_dist <- cbind(row_names,paired_dist)

# Generate distance matrix with samples of the compared groups (column-wise)

paired_dist <- paired_dist[sapply(paired_dist[,1], function(x) all(x %in% inc_groups)),]

# Remove first column with unnecessary group information

paired_dist[,1] <- NULL

paired_dist <- rbind(row_names,paired_dist)

# Generate distance matrix with samples of the compared group (row-wise)

paired_dist <- paired_dist[,sapply(paired_dist[1,], function(x) all(x %in% inc_groups))]

# Remove first row with unnecessary group information

paired_dist <- paired_dist[-1,]

# Convert generated distance matrix to data type matrix (needed by multivariate analysis)

paired_matrix <- as.matrix(paired_dist)

class(paired_matrix) <- "numeric"

# Save paired matrix in list

pairedMatrixList[[i]] <- paired_matrix

# Applies multivariate analysis to a pair out of the selected groups

adonis <- adonis(paired_matrix ~ all_groups_comp[all_groups_comp == pair_1 |

all_groups_comp == pair_2])

# List p-values

pVal[i] <- adonis[[1]][6][[1]][1]

}

# Adjust p-values for multiple testing according to Benjamini-Hochberg method

pVal_BH <- p.adjust(pVal,method="BH", n=length(pVal))

# Generated NMDS plots are stored in one pdf file called "pairwise-beta-diversity-nMDS.pdf"

file_name <- paste(group_name,"pairwise-beta-diversity-NMDS.pdf",sep="_")

pdf(paste(group_name,"/",file_name,sep=""))

for(i in 1:length(combn(unique_groups,2)[1,])){

meta <- metaMDS(pairedMatrixList[[i]], k = 2)

s.class(

meta$points,

col = rainbow(length(levels(all_groups_comp))), cpoint = 2,

fac = as.factor(all_groups_comp[all_groups_comp == pair_1_list[i] |

all_groups_comp == pair_2_list[i]]),

sub = paste("NMDS plot of Microbial Profiles\n ",pair_1_list[i]," - ",pair_2_list[i], "\n(p-value ",pVal[i],","," corr. p-value ", pVal_BH[i],")",sep="")

)

}

dev.off()

# Generated MDS plots are stored in one pdf file called "pairwise-beta-diversity-MDS.pdf"

file_name <- paste(group_name,"pairwise-beta-diversity-MDS.pdf",sep="_")

pdf(paste(group_name,"/",file_name,sep=""))

for(i in 1:length(combn(unique_groups,2)[1,])){

# Calculate and display the MDS plot (Multidimensional Scaling plot)

s.class(

cmdscale(pairedMatrixList[[i]], k = 2), col = rainbow(length(levels(all_groups_comp))), cpoint =

2, fac = as.factor(all_groups_comp[all_groups_comp == pair_1_list[i] |

all_groups_comp == pair_2_list[i]]), sub = paste("MDS plot of Microbial Profiles\n ",pair_1_list[i]," - ",pair_2_list[i], "\n(p-value ",pVal[i],","," corr. p-value ", pVal_BH[i],")",sep="")

)

}

dev.off()

}

#################################################################################

###### **Write Output Files** ######

#################################################################################

# Write the distance matrix table in a file

file_name <- paste(group_name,"distance-matrix-gunif.tab",sep="_")

write.table( unifract_dist_comp, paste(group_name,"/",file_name,sep=""), sep = "\t", col.names = NA, quote = FALSE)

write.table( unifract_dist_comp, "distance-matrix-gunif.tab", sep = "\t", col.names = NA, quote = FALSE)

write.tree(tree,"samples-Tree.nwk",tree.names = FALSE)

# Graphical output files are generated in the main part of the script

if(!flag) { stop("

It was not possible to install all required R libraries properly.

Please check the installation of all required libraries manually.\n

Required libaries:ade4, GUniFrac, phangorn")

}

#################################################################################

###### **End of Script** ######

#################################################################################
