# Supplementary figures and images for: SalmoSim: the development of a three-compartment in vitro simulator of the Atlantic salmon GI tract and associated microbial communities
Source: Microbiome. 2021 Aug 31;9:179. doi: 10.1186/s40168-021-01134-6 (PMC8408954; doi:10.1186/s40168-021-01134-6)

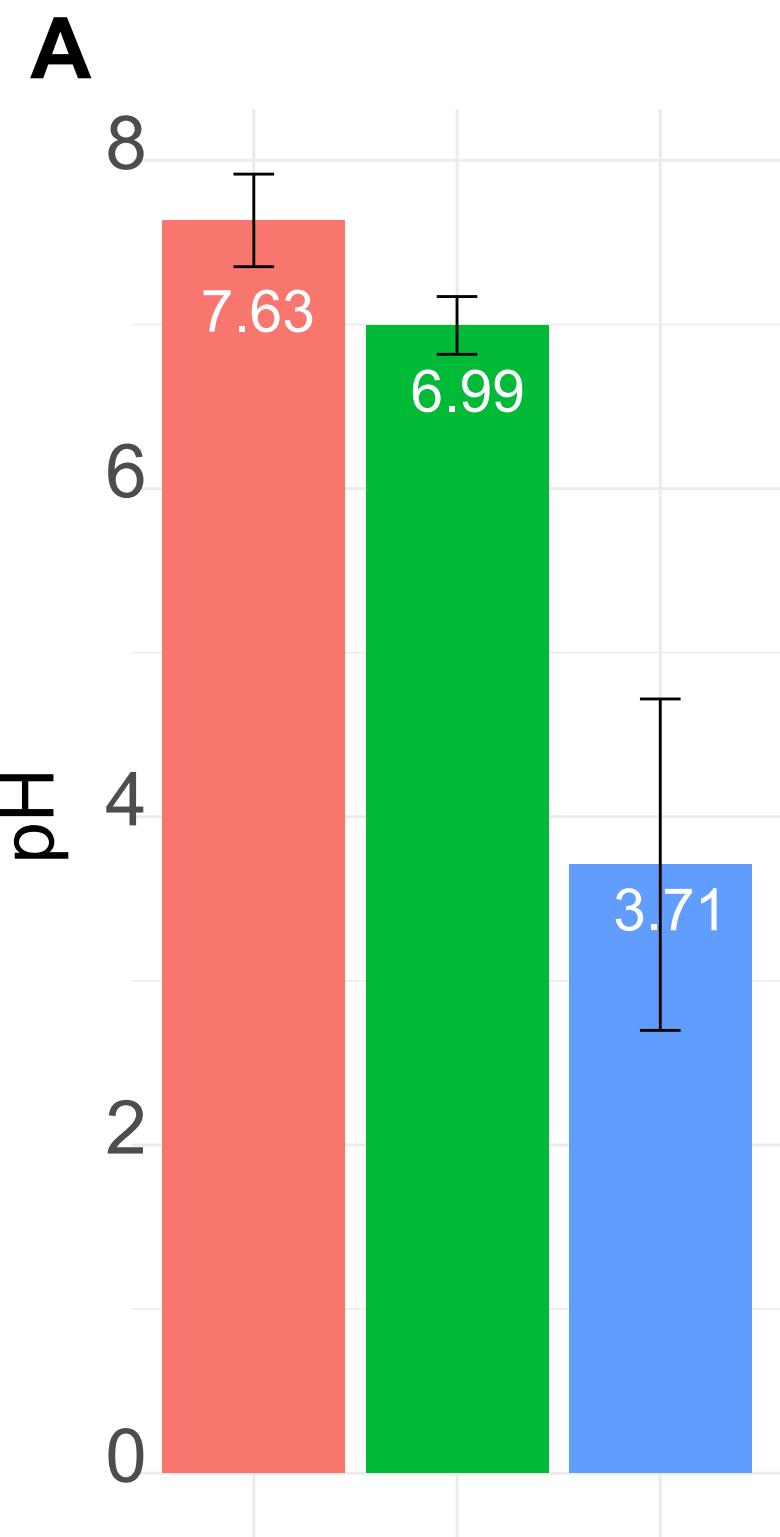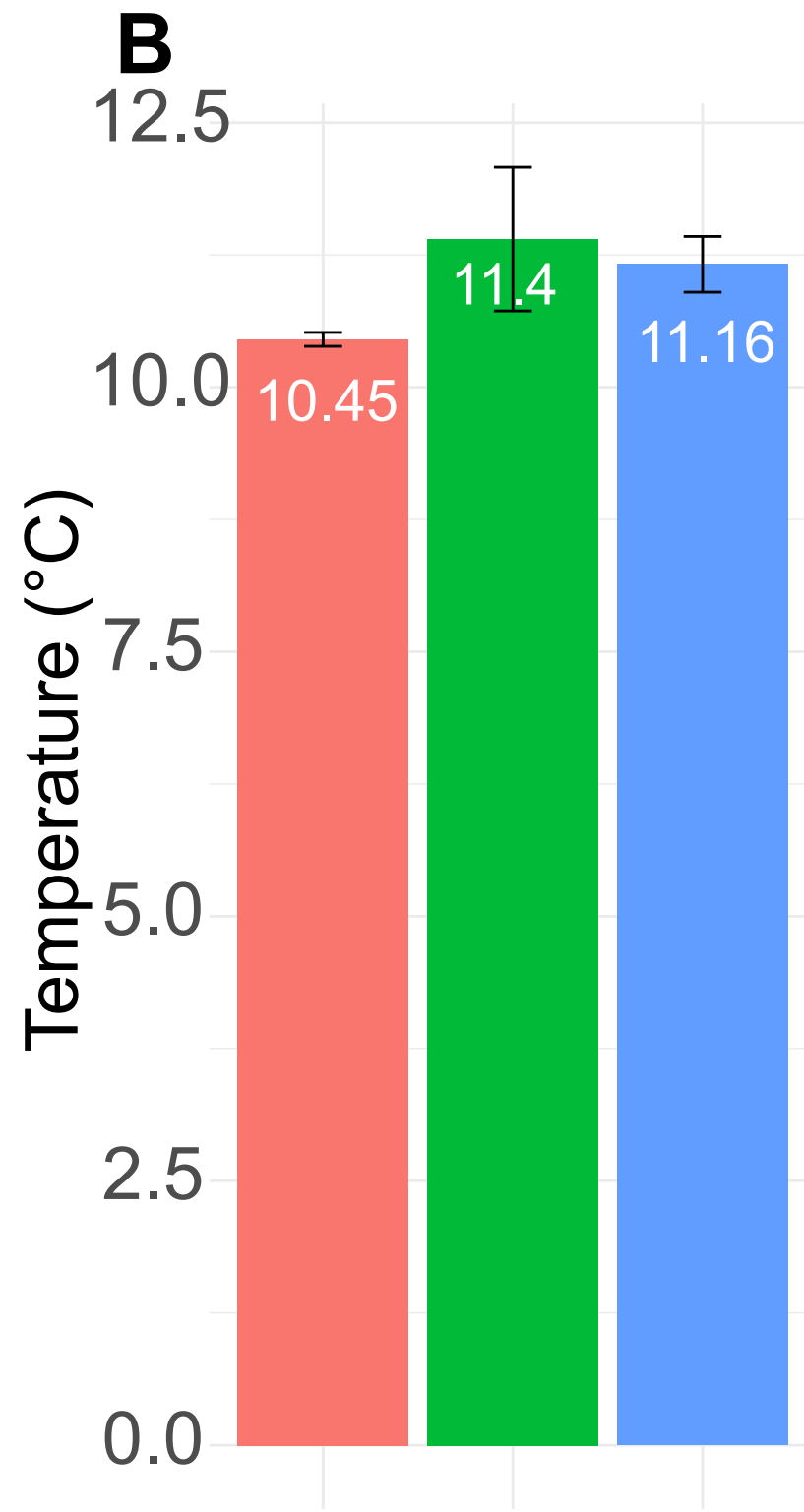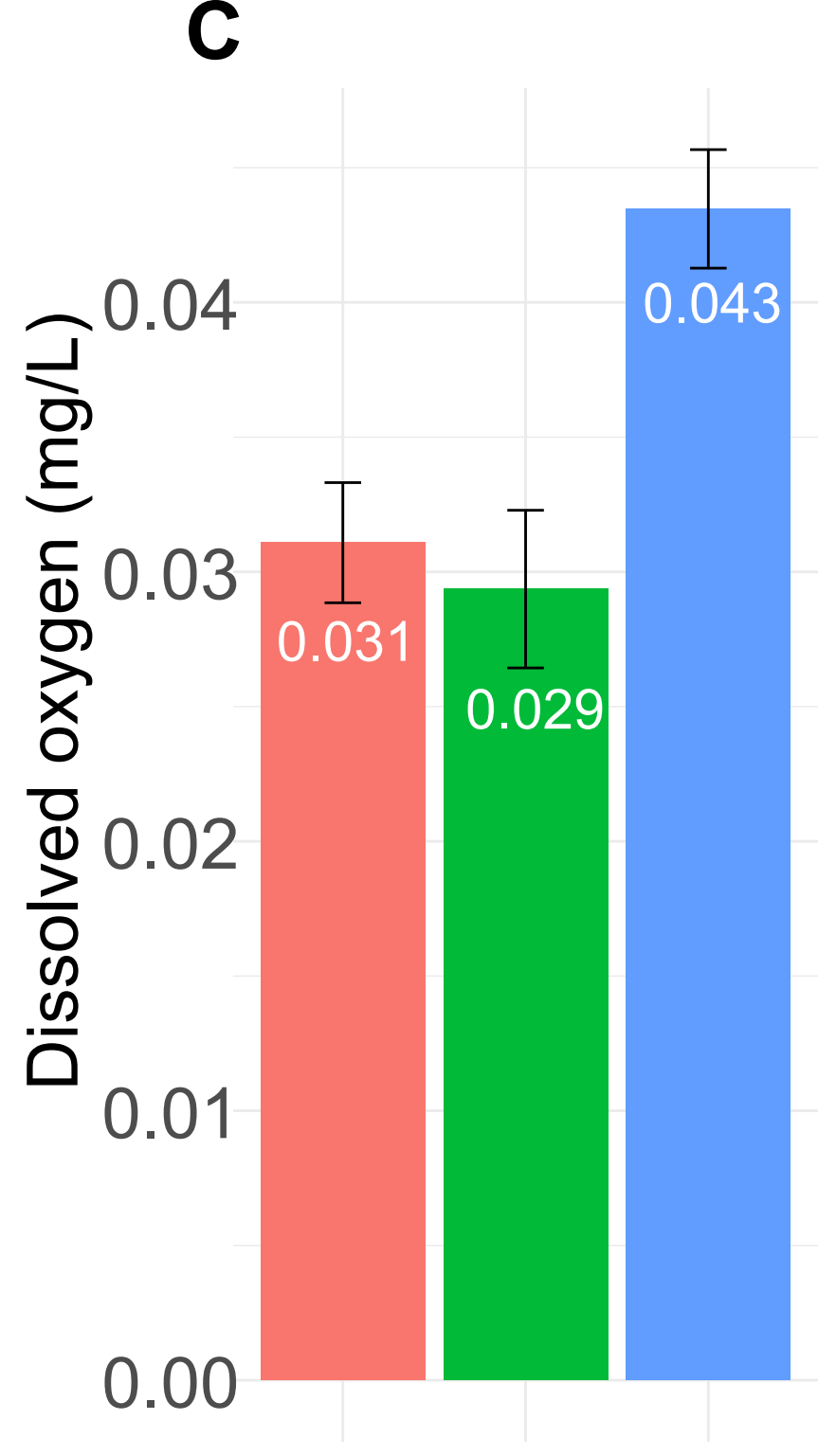

Compartment    Mid gut    Pyloric Caeca    Stomach

Supplement: Supplementary file 8 — Additional file 7: Figure S1. Physiochemical conditions measured within different real Atlantic salmon gut compartments. 1A-1C measured physicochemical conditions within real salmon (n=3) gut compartments: pH (1A), temperature (°C, 1B), dissolved oxygen (mg/L, 1C). [file 40168_2021_1134_MOESM8_ESM.pdf]

**A**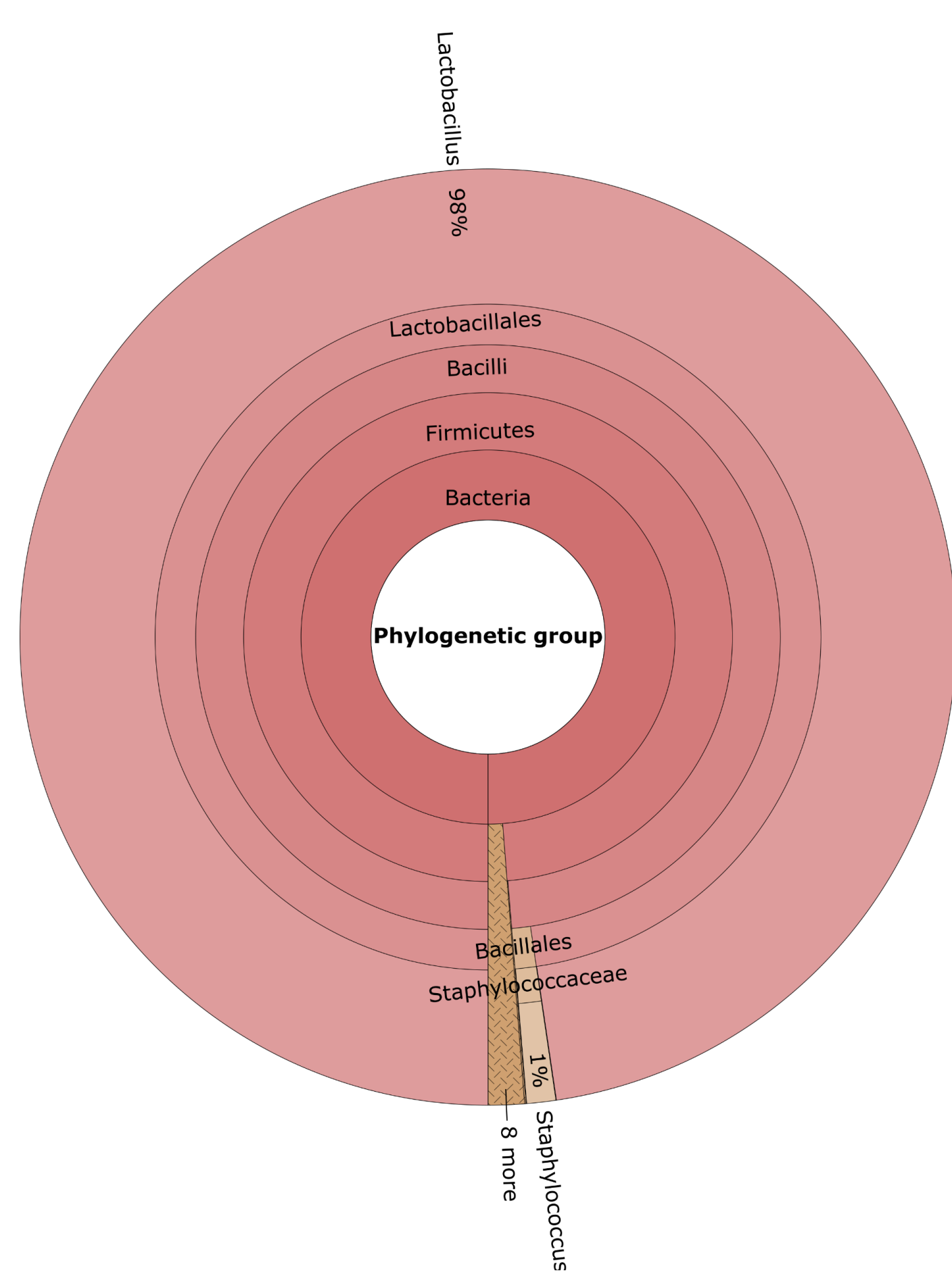**Fish 1****B**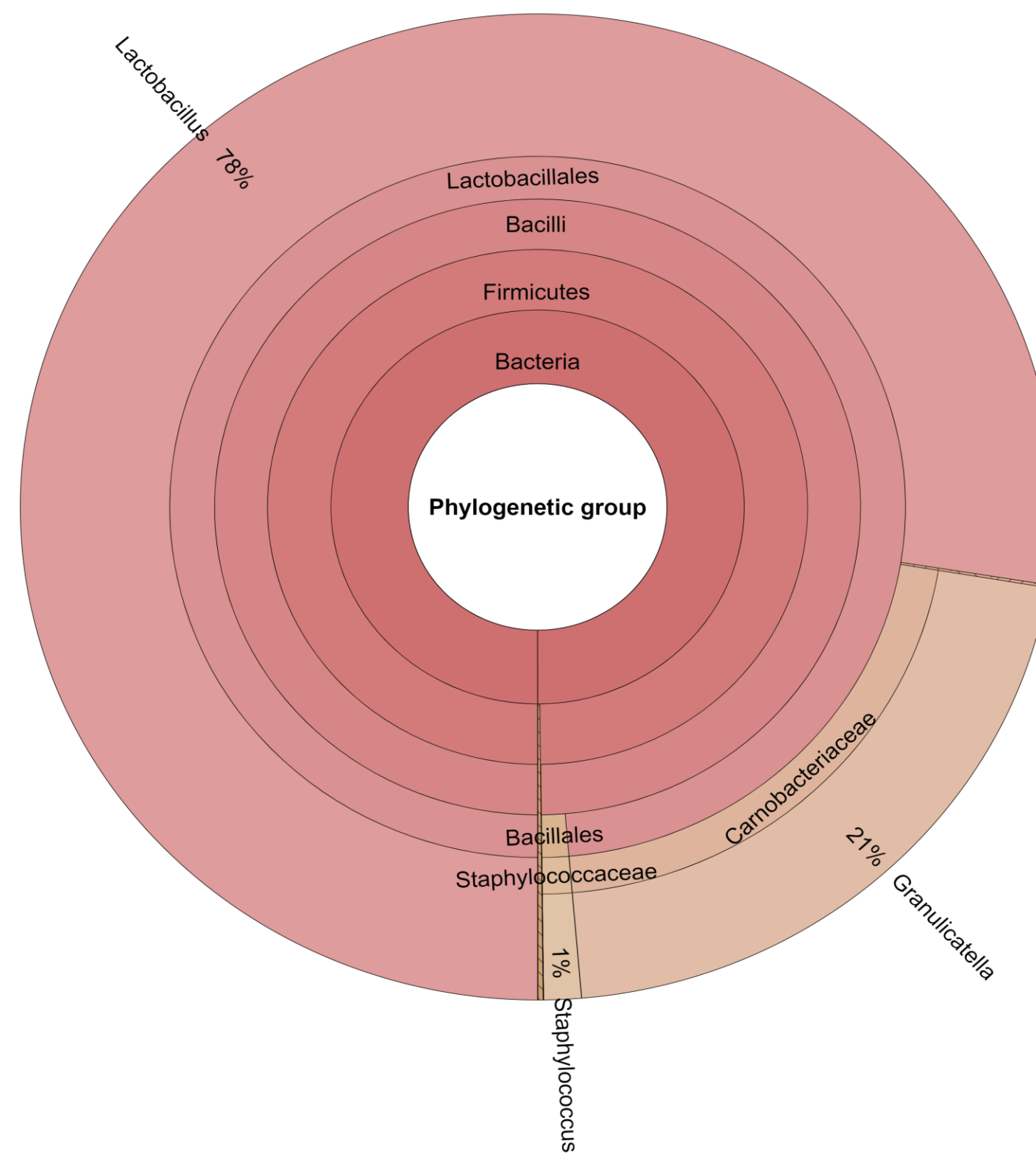**Fish 2****C**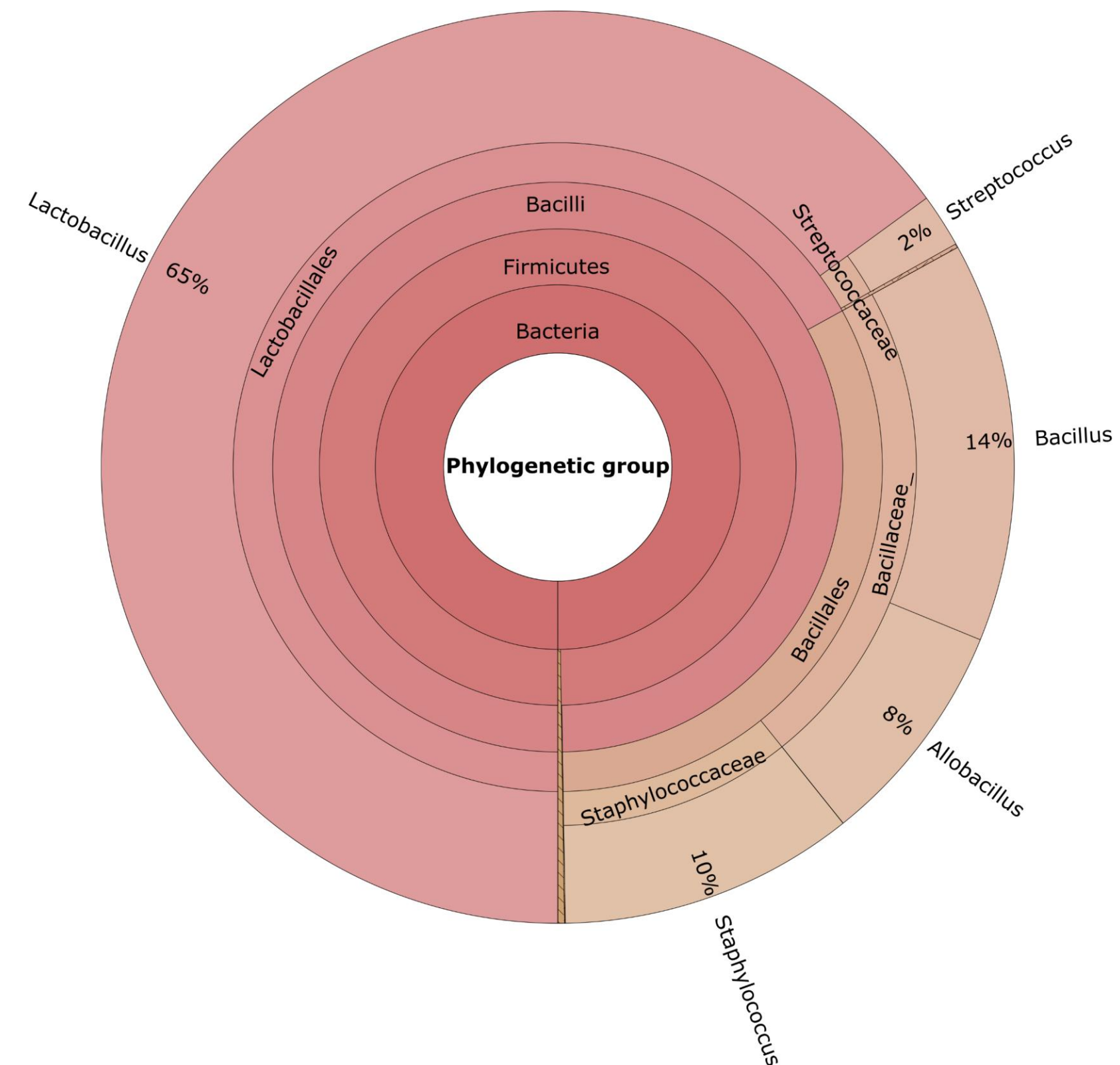**Fish 3****D**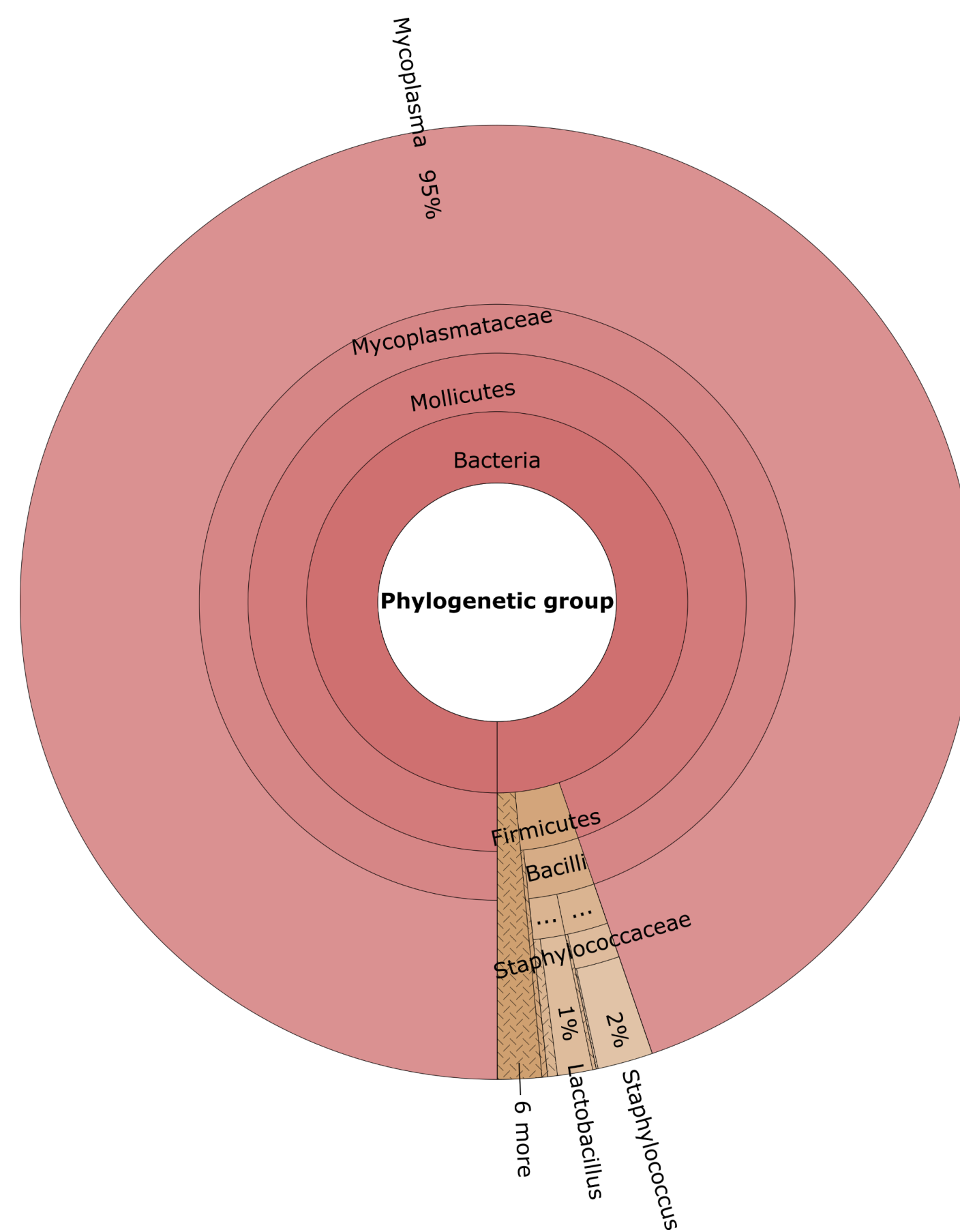**Fish 1****E**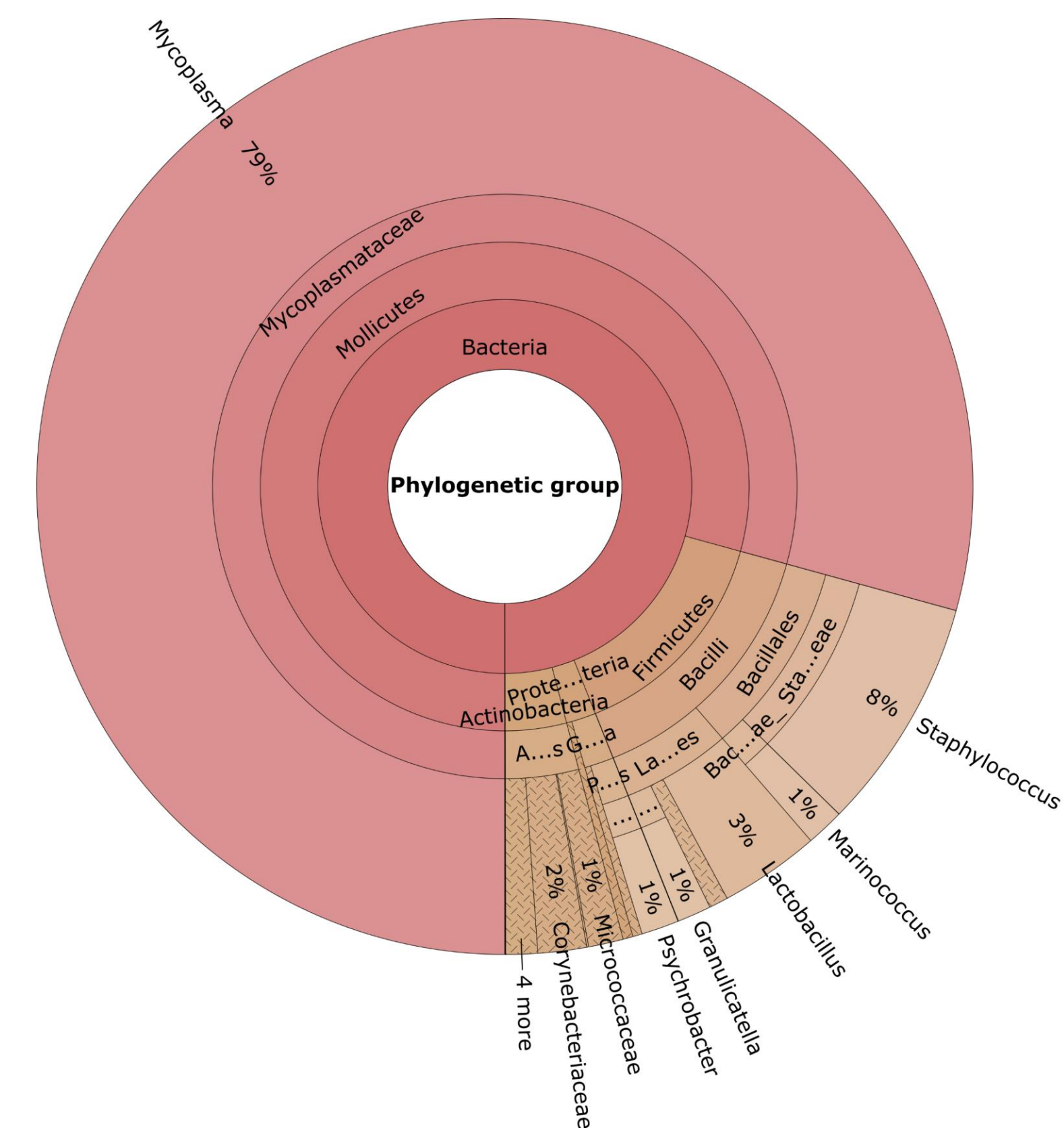**Fish 2****F**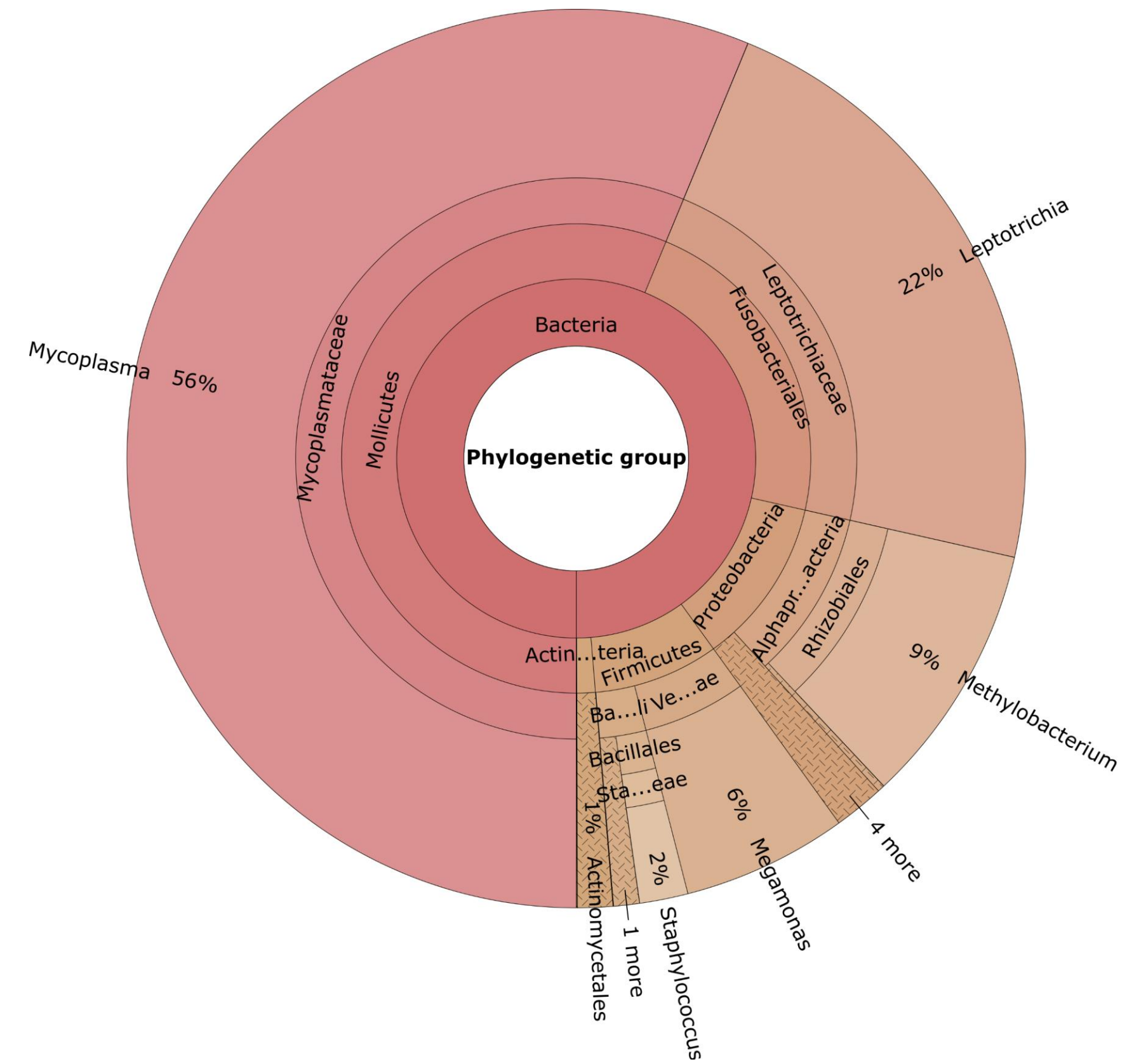**Fish 3**

Supplement: Supplementary file 9 — Additional file 8: Figure S2. Specificity of the primers that target Lactobacillus and Mycoplasma genus. The results in figure summarise bacterial genus targeted by Lactobacillus (Fig. 1 A, B, C) and Mycoplasma (Fig. 1 D, E, F) specific primer set. It shows that of all genus captured by Lactobacillus primer pair 98% were Lactobacillus in fish 1, 78% in fish 2, and 65% in fish 3. While of all genus captured by Mycoplasma primer pair 95% were Mycoplasma in fish 1, 79% in fish 2, and 56% in fish 3. [file 40168_2021_1134_MOESM9_ESM.pdf]

Stomach

Fish meal

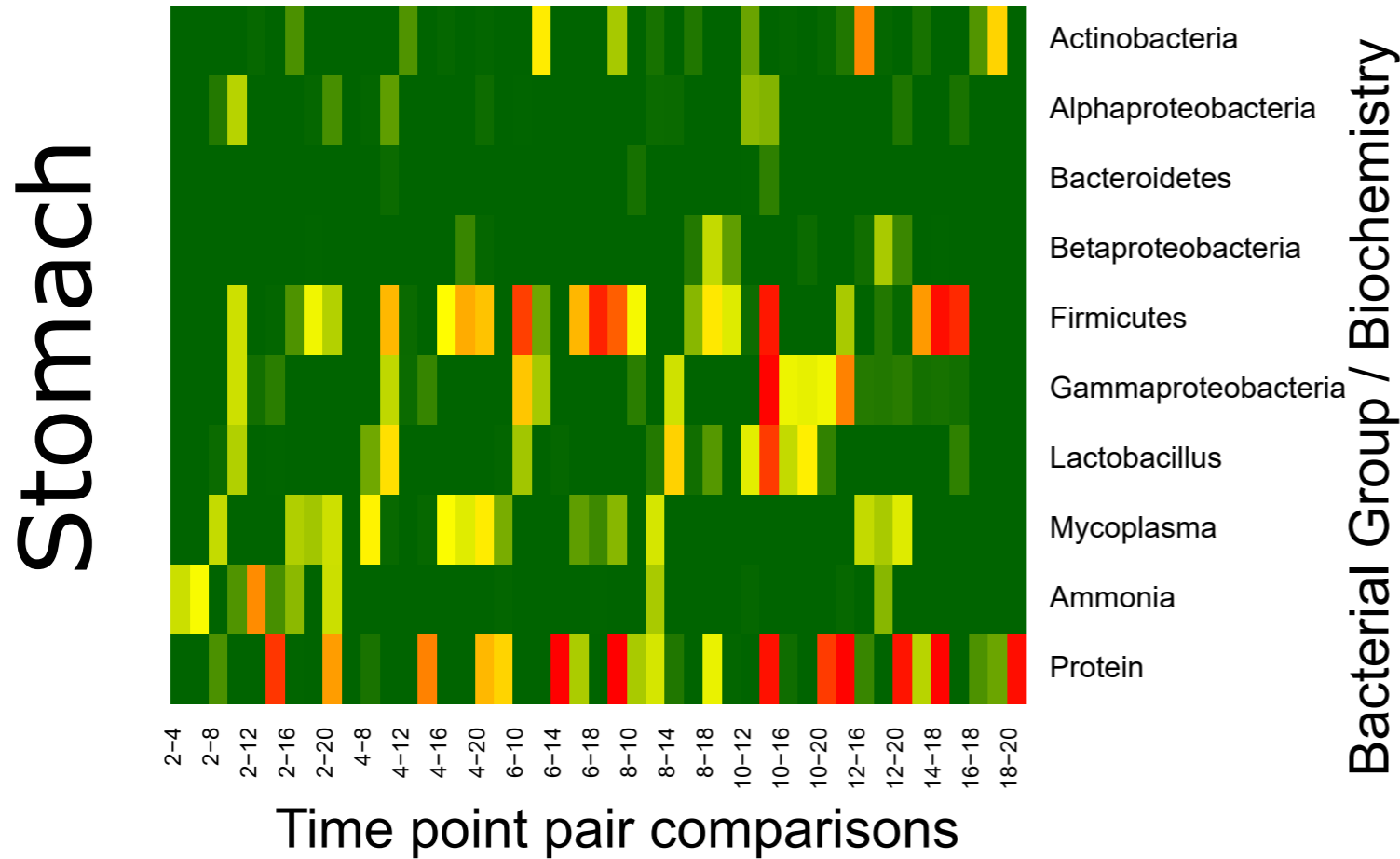

Fish meal free

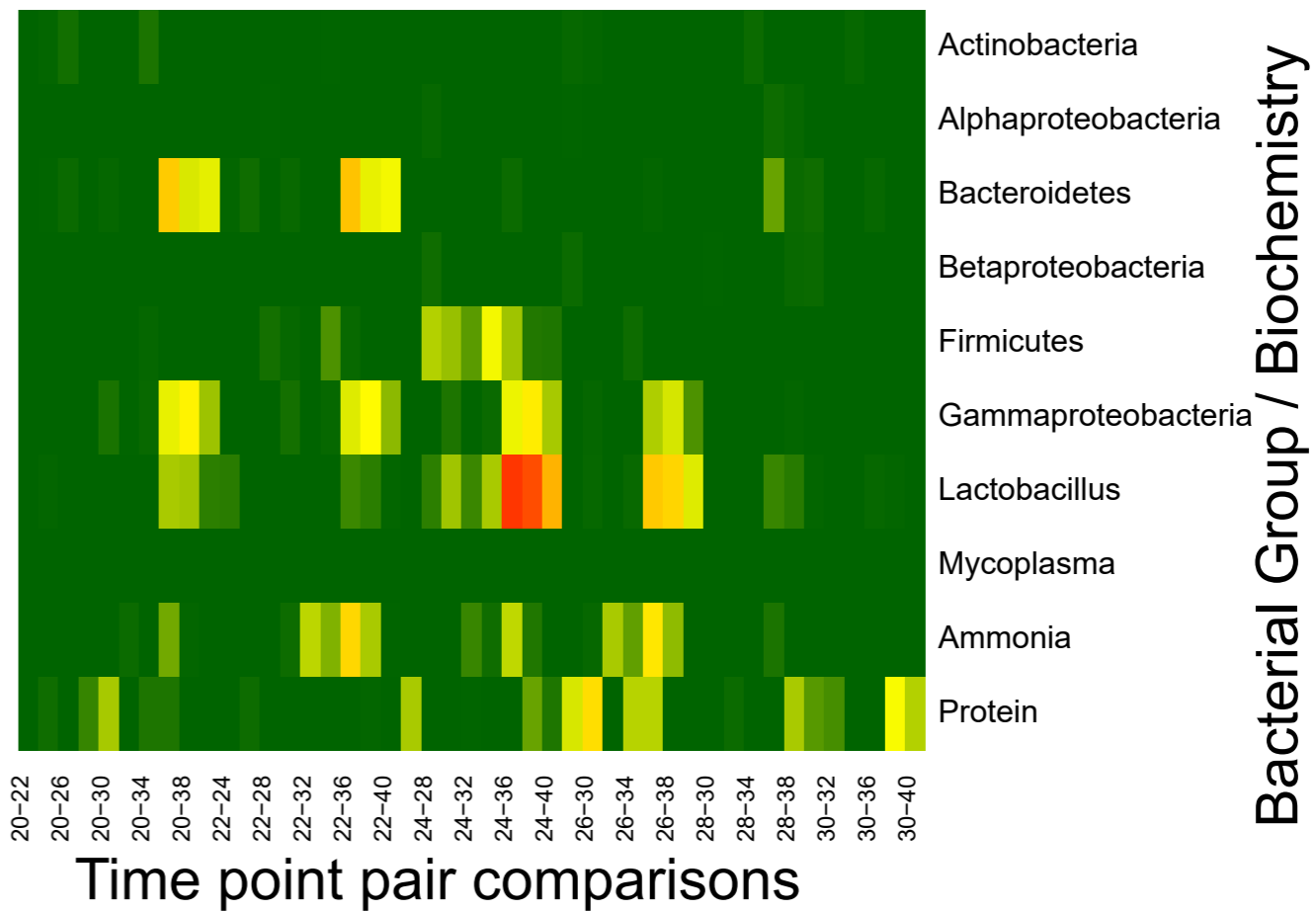

Pyloric caeca

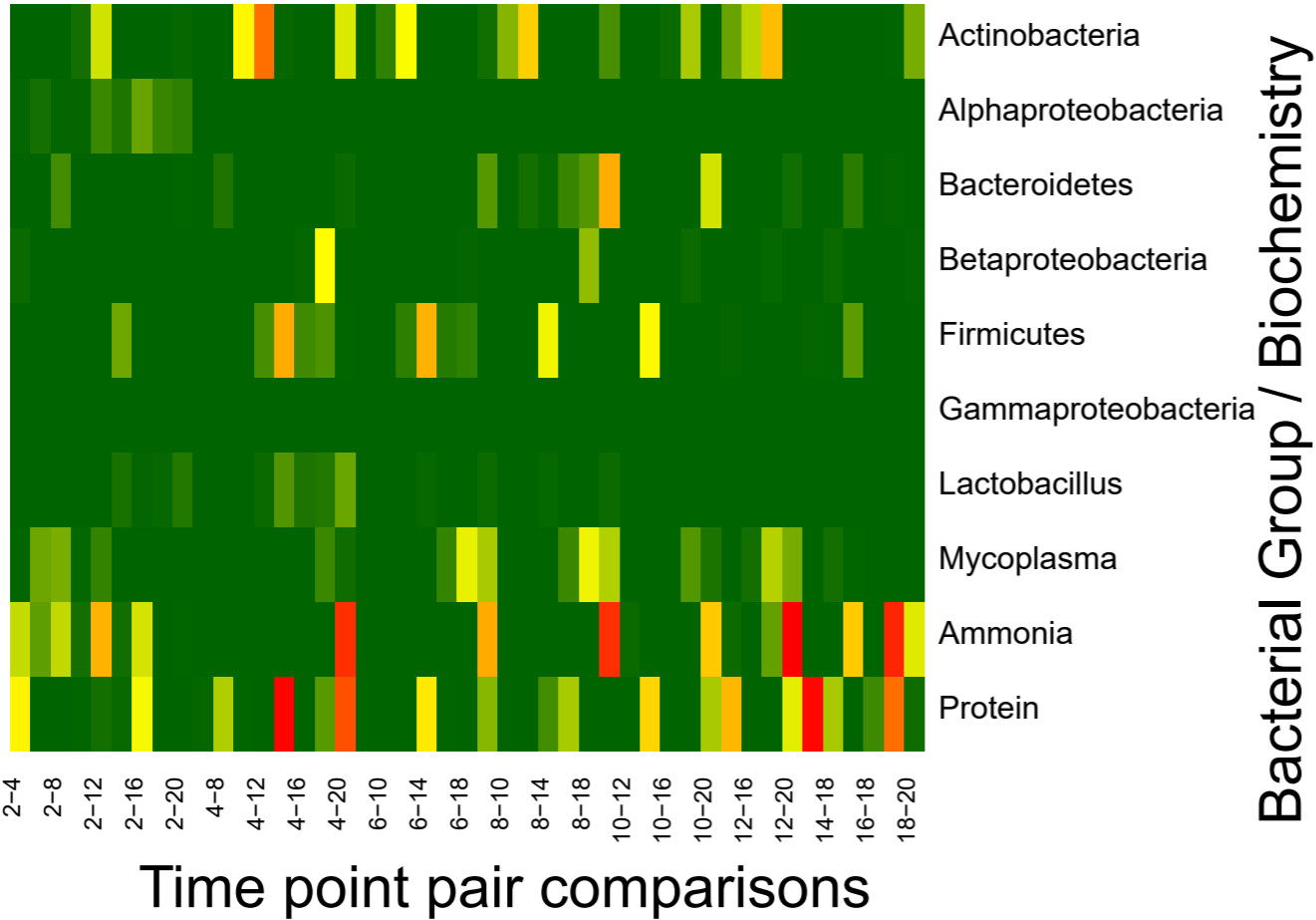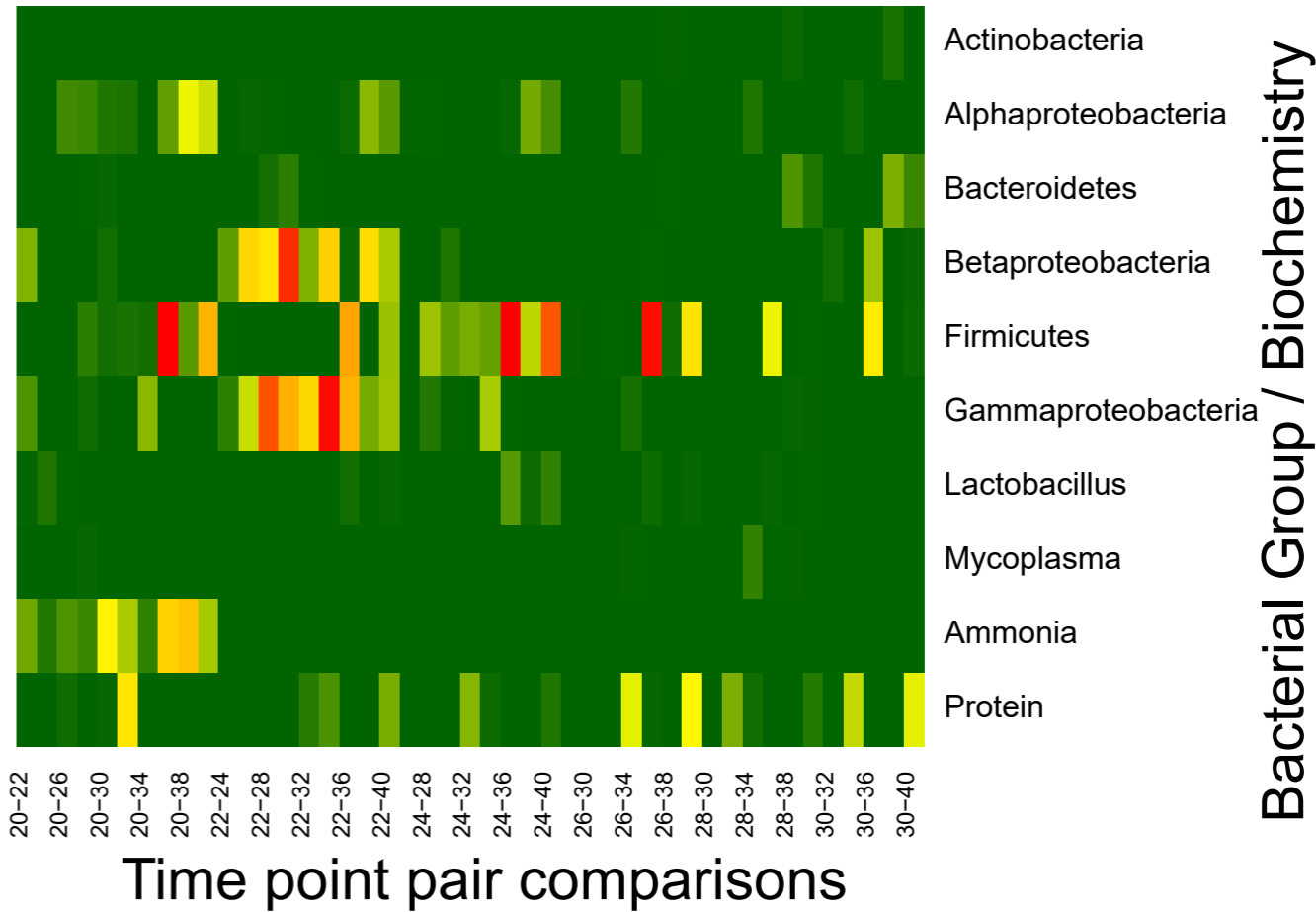

Mid gut

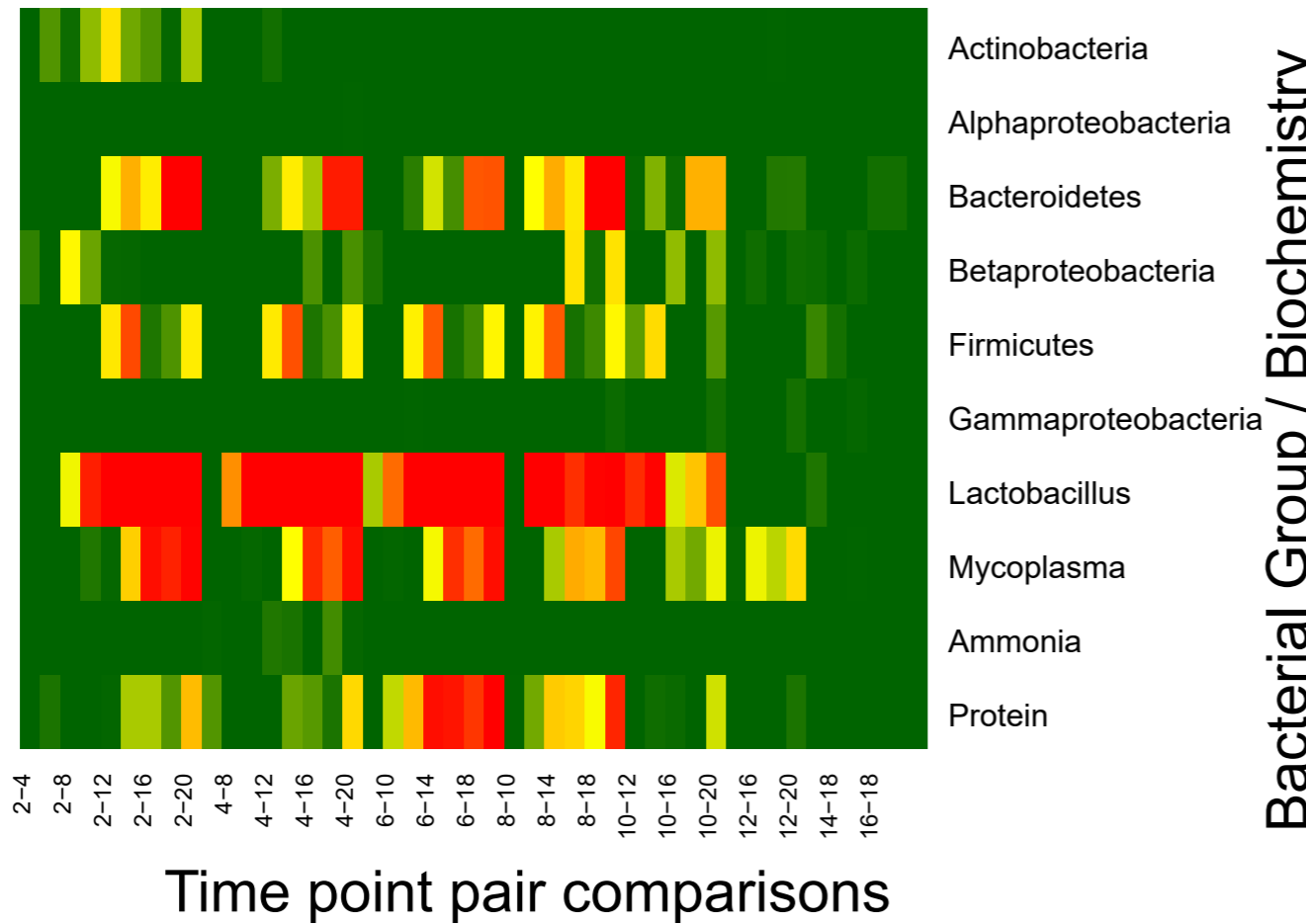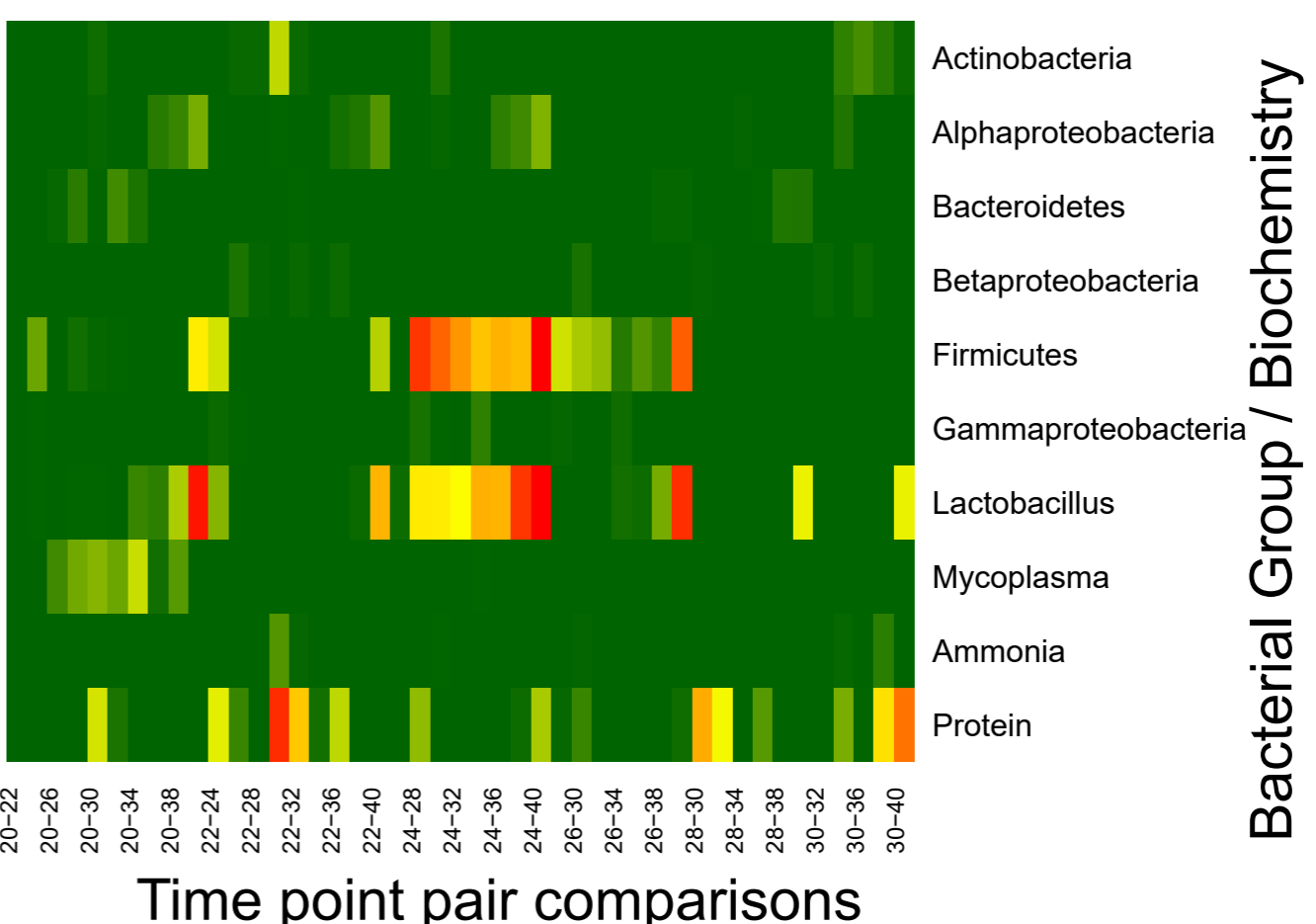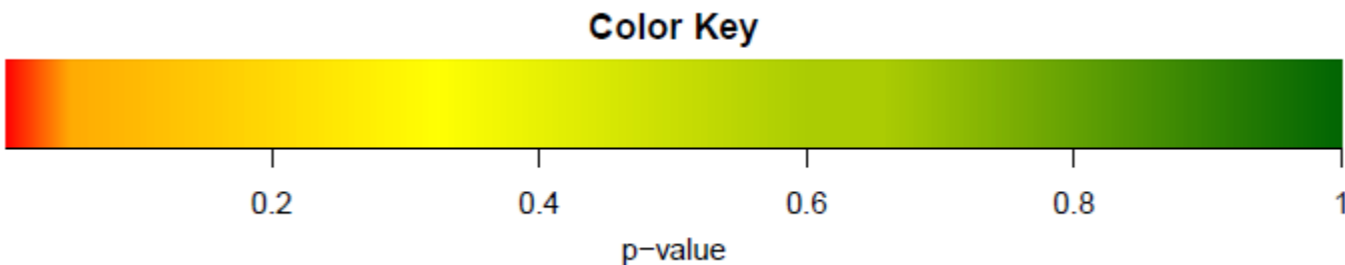

Supplement: Supplementary file 11 — Additional file 10: Figure S4. Measured value (qPCR, ammonia and protein concentrations) stability within different SalmoSim compartments fed on Fish meal and Fish meal free diets. The figure summarises the Estimated Marginal Means output for each mixed-effect linear model (Model 1) run with different values measured in different SalmoSim compartments (qPCR measurements, ammonia and protein concentrations) identifying the difference between different time points during the first (system fed on Fish meal diet) and last 20 days (system fed on Fish meal free diet) of validation experiment. A small p-value indicates that the two time points are statistically different, and p>0.05 indicates that two time points are not statistically different. The colour key illustrates the p-value: red end of spectrum denoting low p values (low correlation between time points) and dark green indicating high p values (no differences between timepoints). [file 40168_2021_1134_MOESM11_ESM.pdf]

Stomach

Fish 7

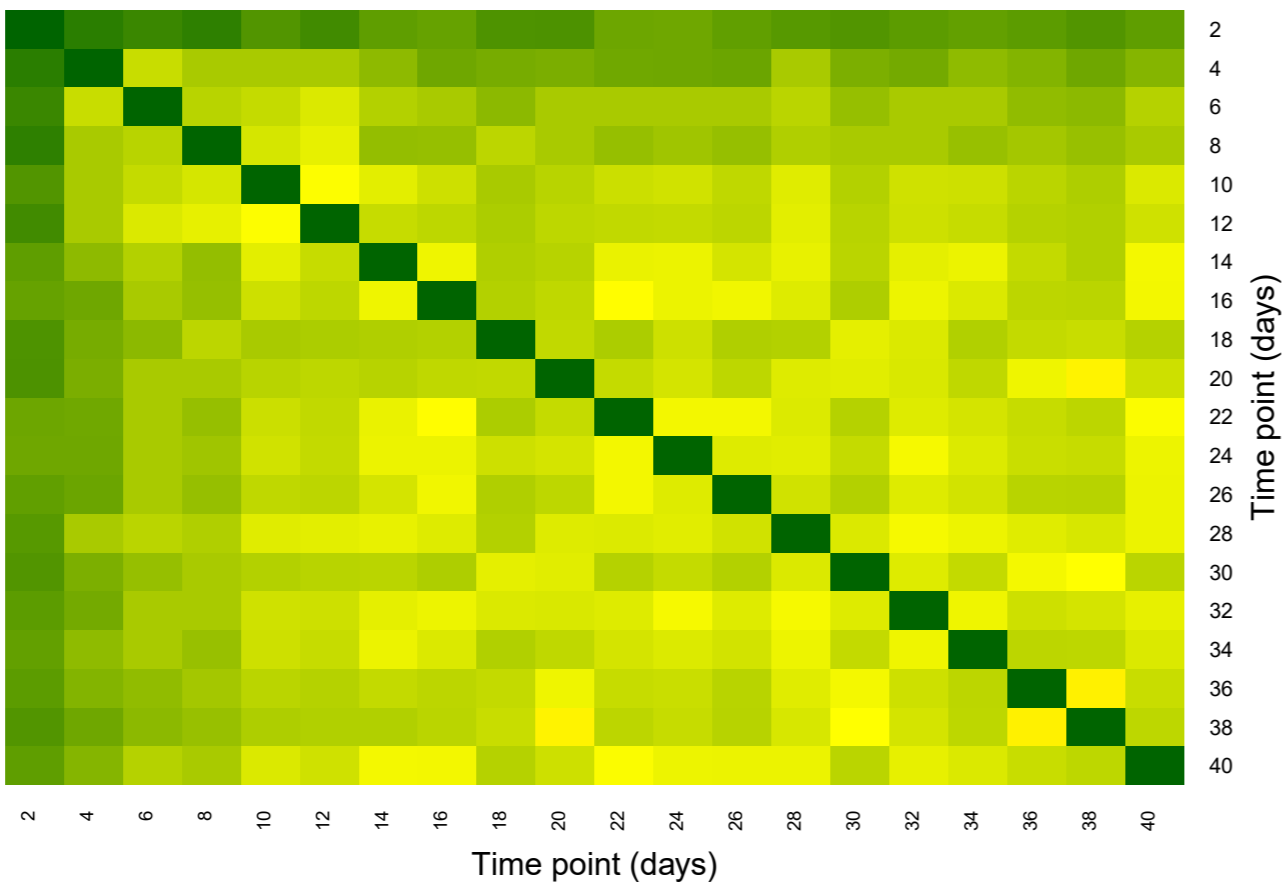

Fish 8

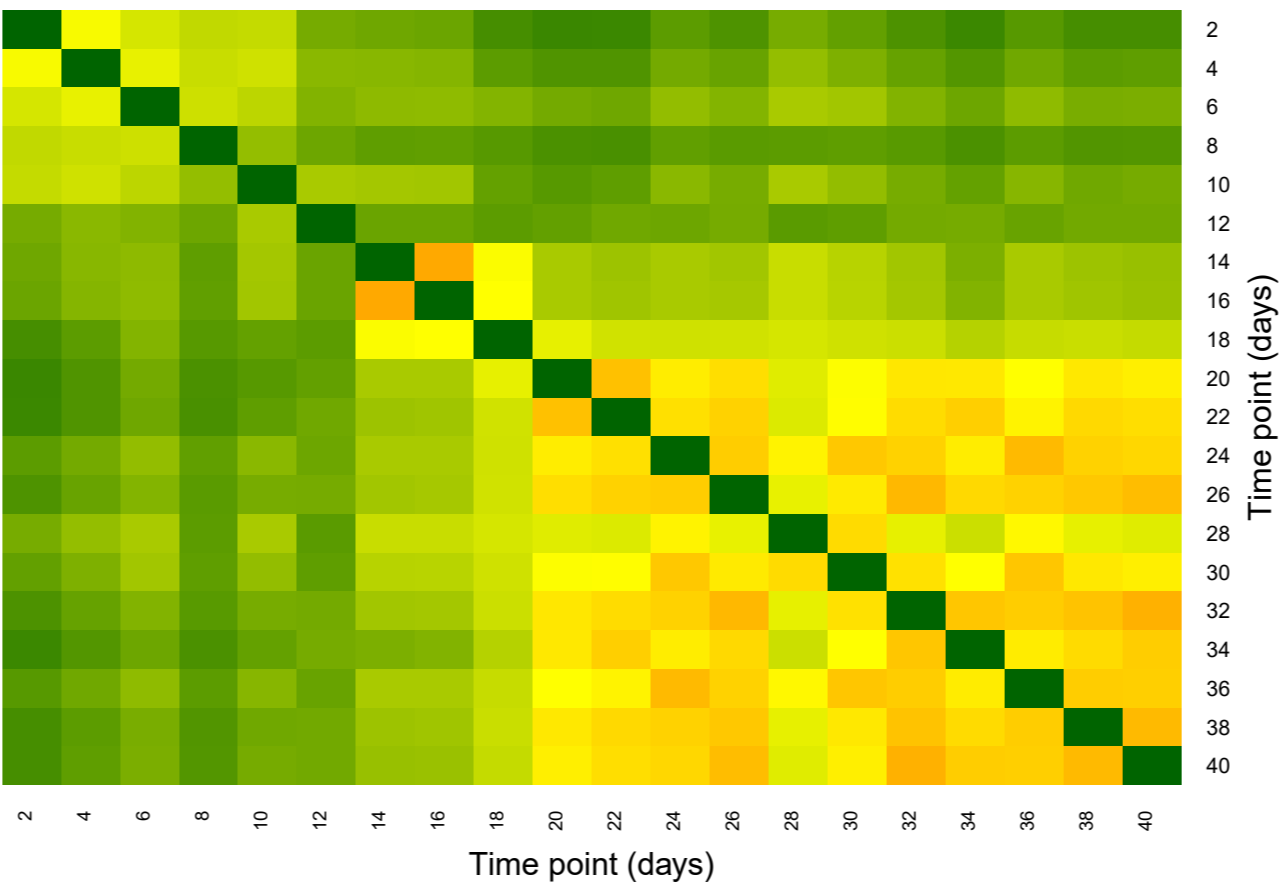

Fish 9

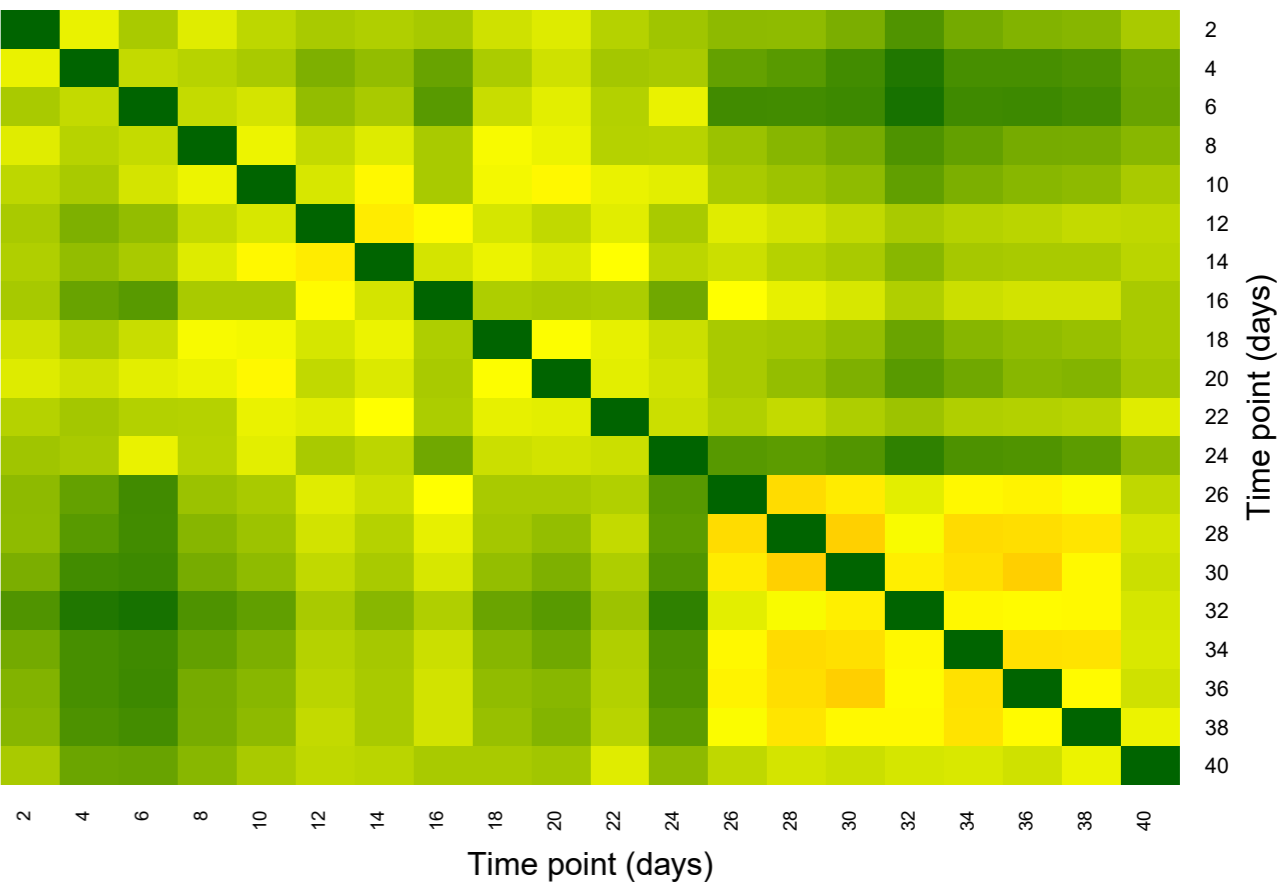

Pyloric  
caecum

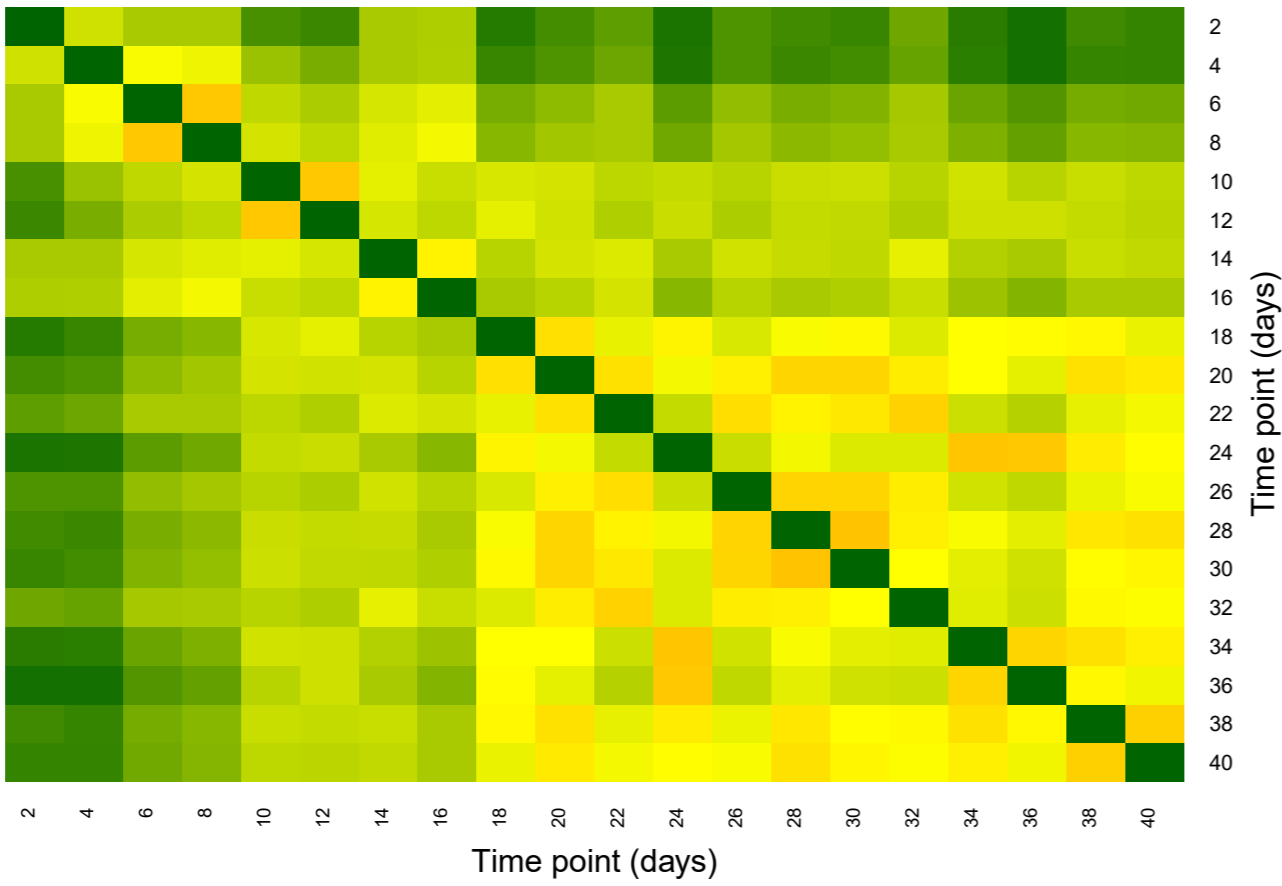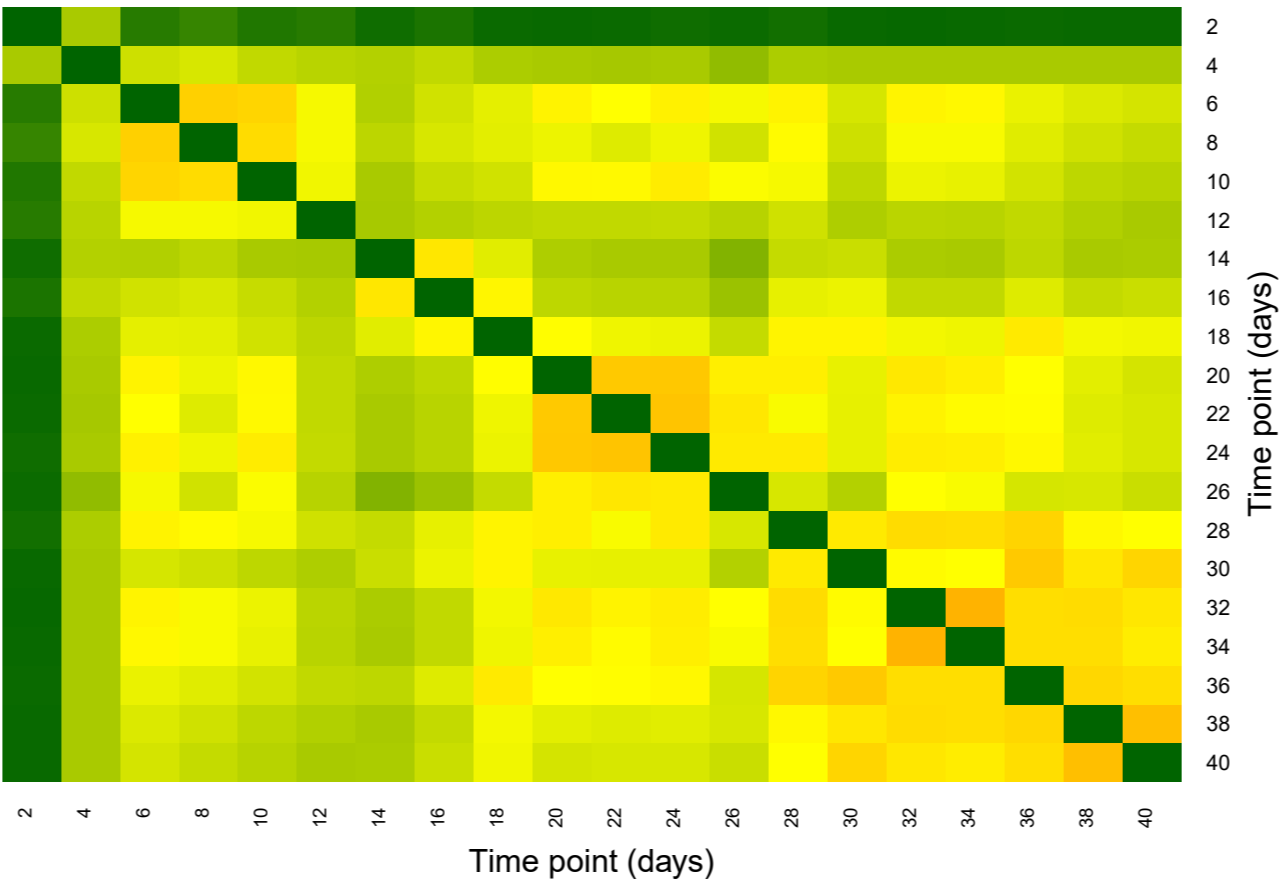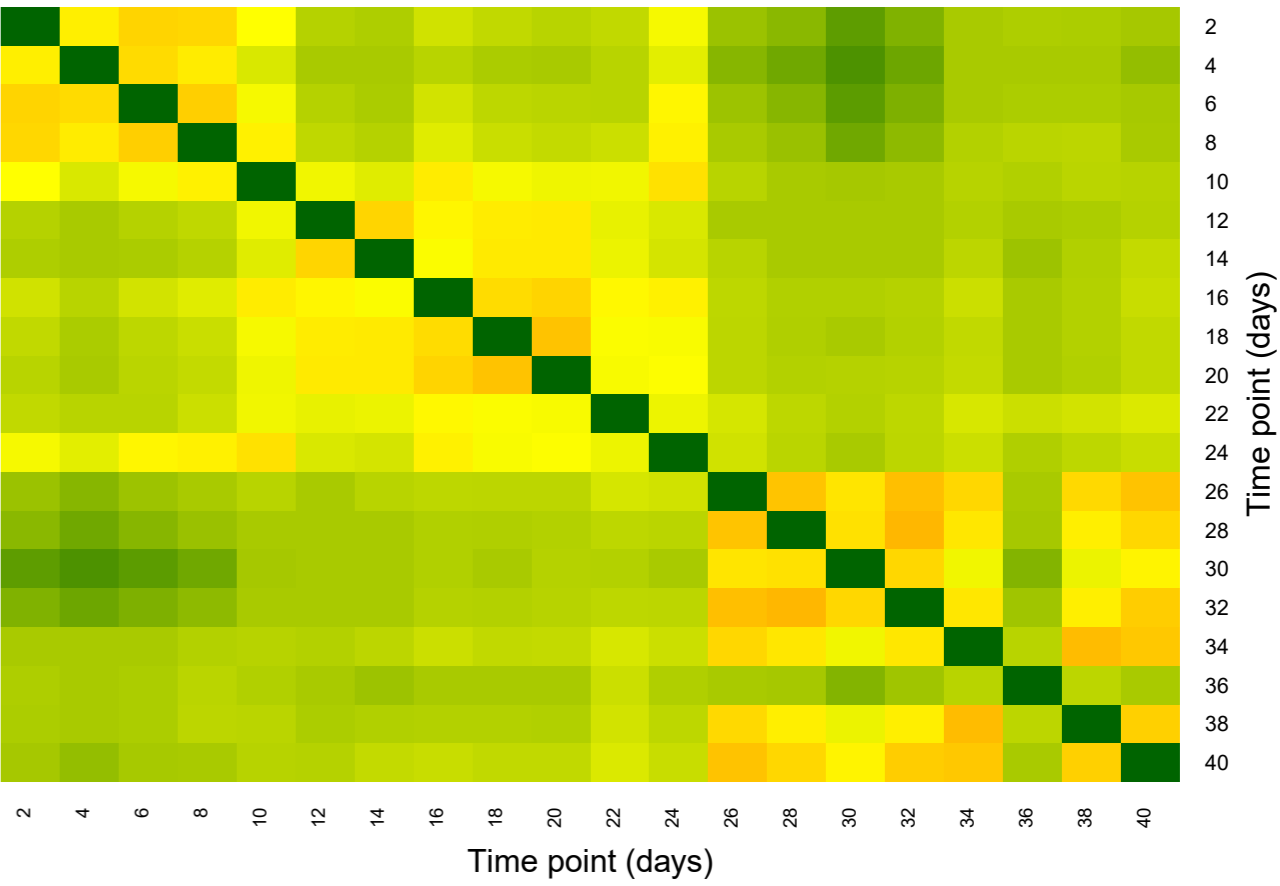

Mid gut

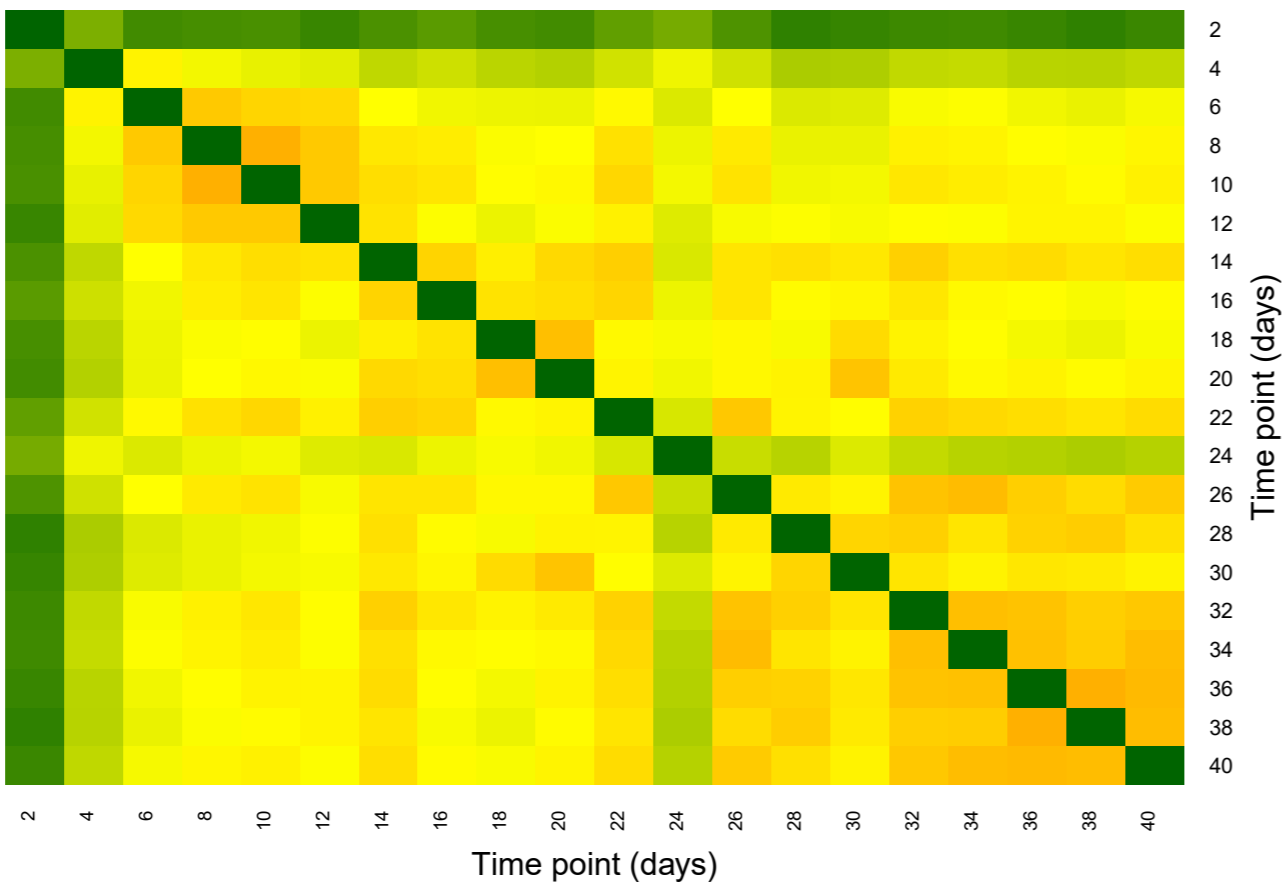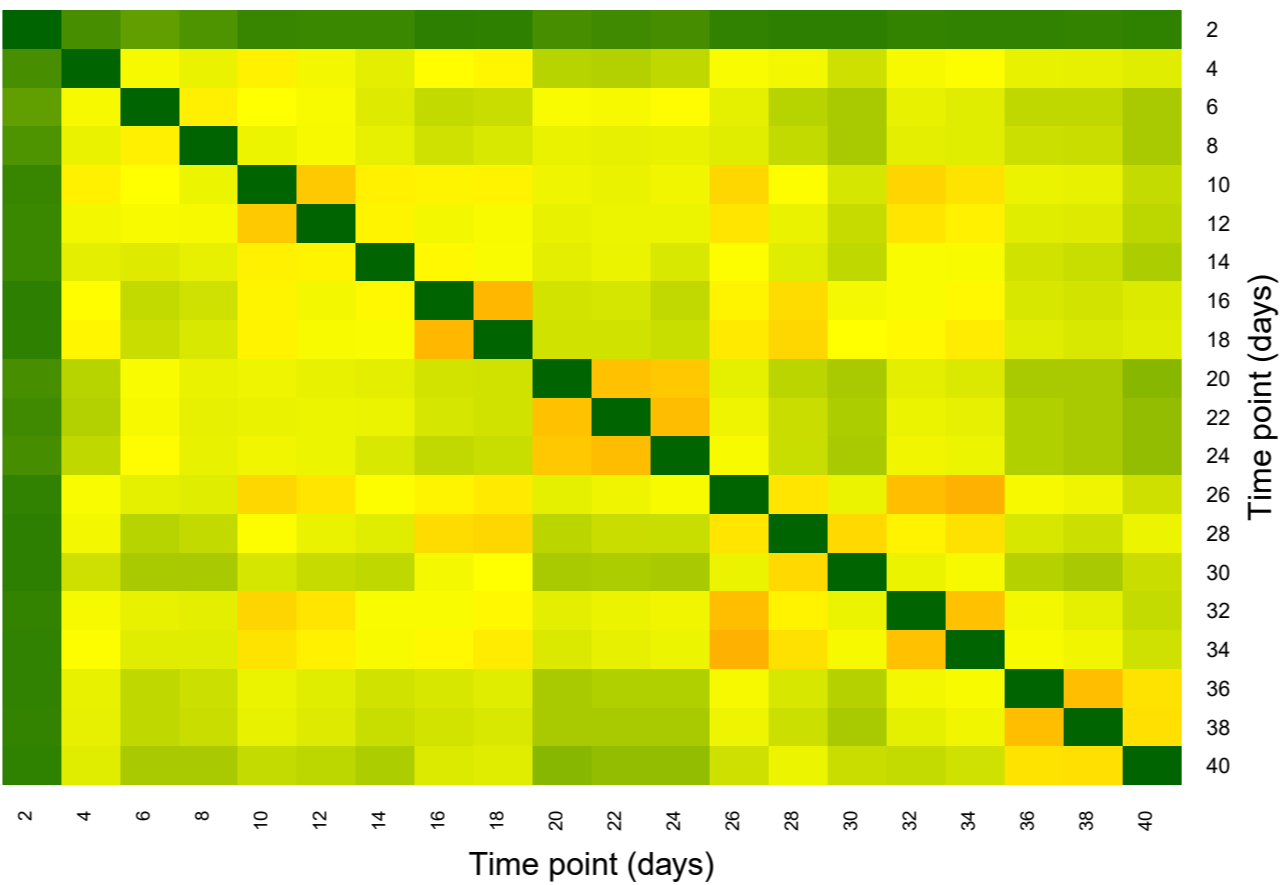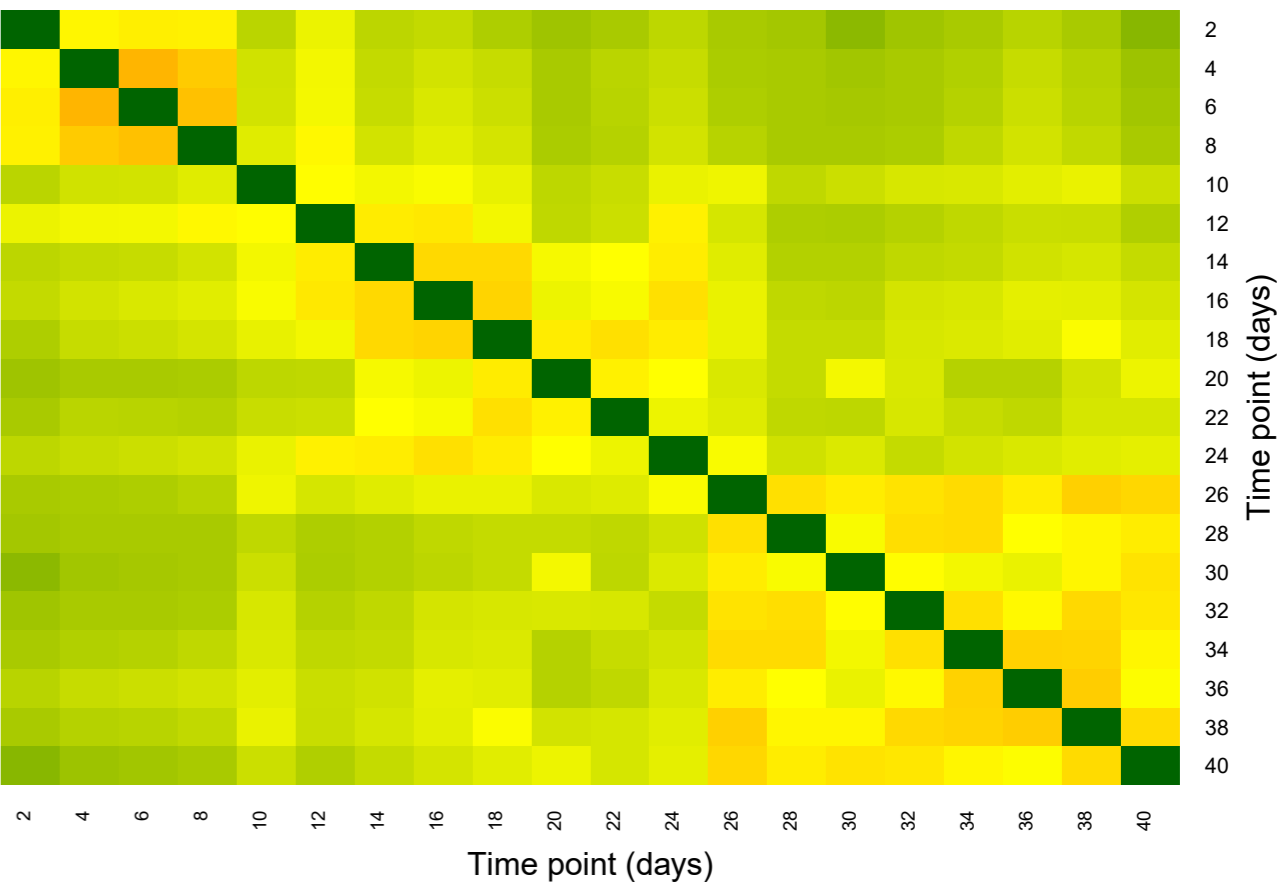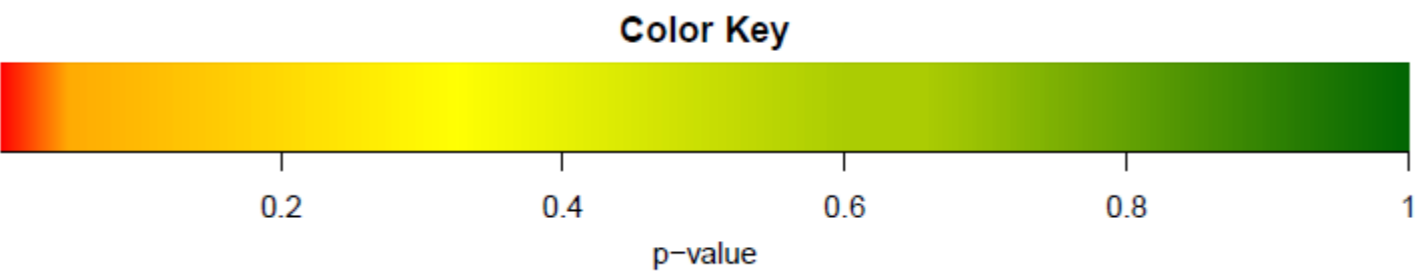

Supplement: Supplementary file 12 — Additional file 11: Figure S5. Stability within SalmoSim system, within different biological replicates and different gut compartments, calculated by using generalised UniFrac values for pairwise beta diversity analysis. The figure represents microbial stability within the SalmoSim system (data separated by different biological replicates and gut compartments) as the pairwise beta diversity comparison between different sampling time points (days), calculated by using generalised (50%) UniFrac as a distance measure. A small p-value indicates that the two time points are statistically different, and p>0.05 indicates that two time points are not statistically different. The colour key illustrates the p-value: red end of spectrum denoting low p values (distinct compositions between time points) and dark green indicating high p values (similar compositions between timepoints). [file 40168_2021_1134_MOESM12_ESM.pdf]

**A**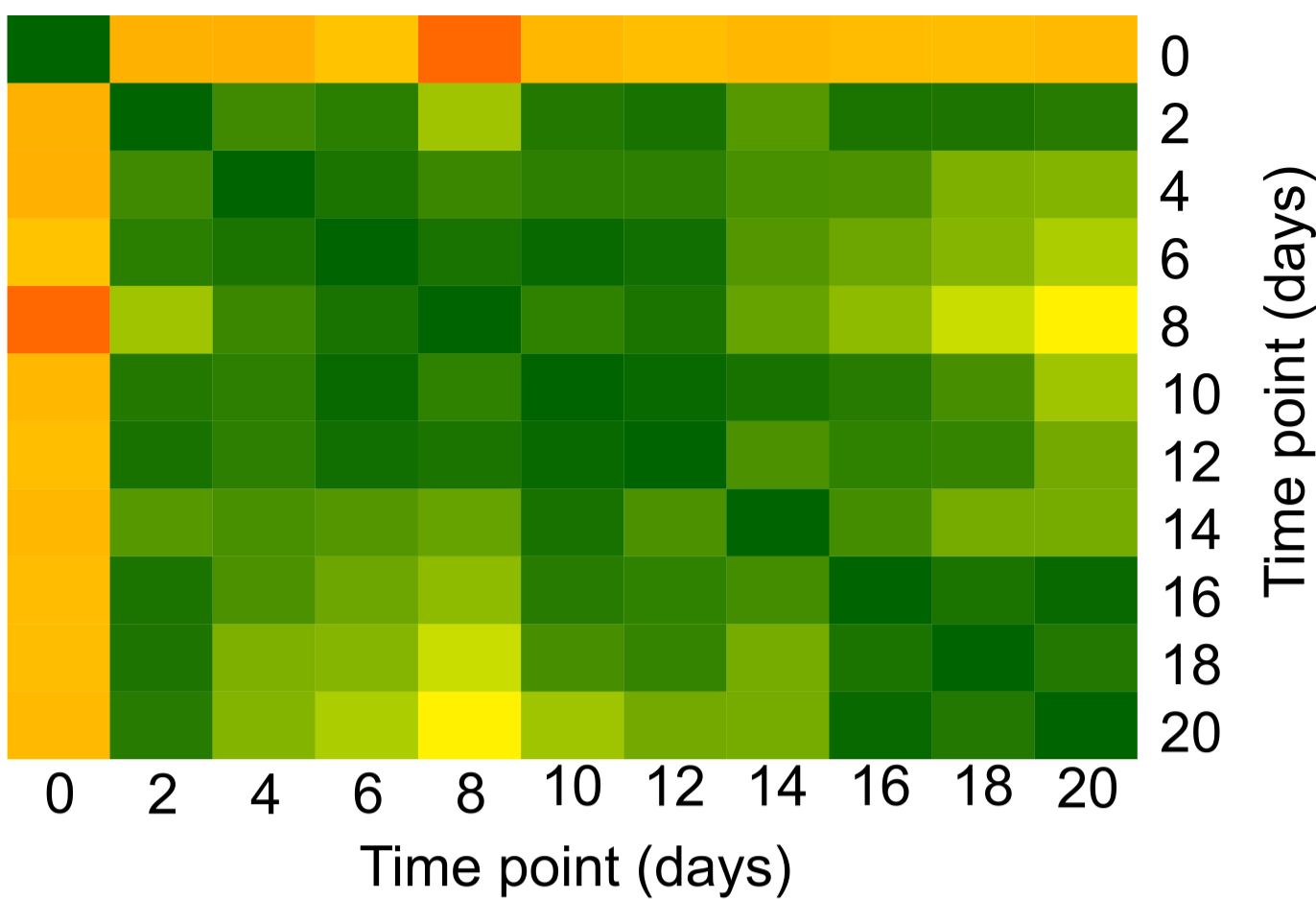**B**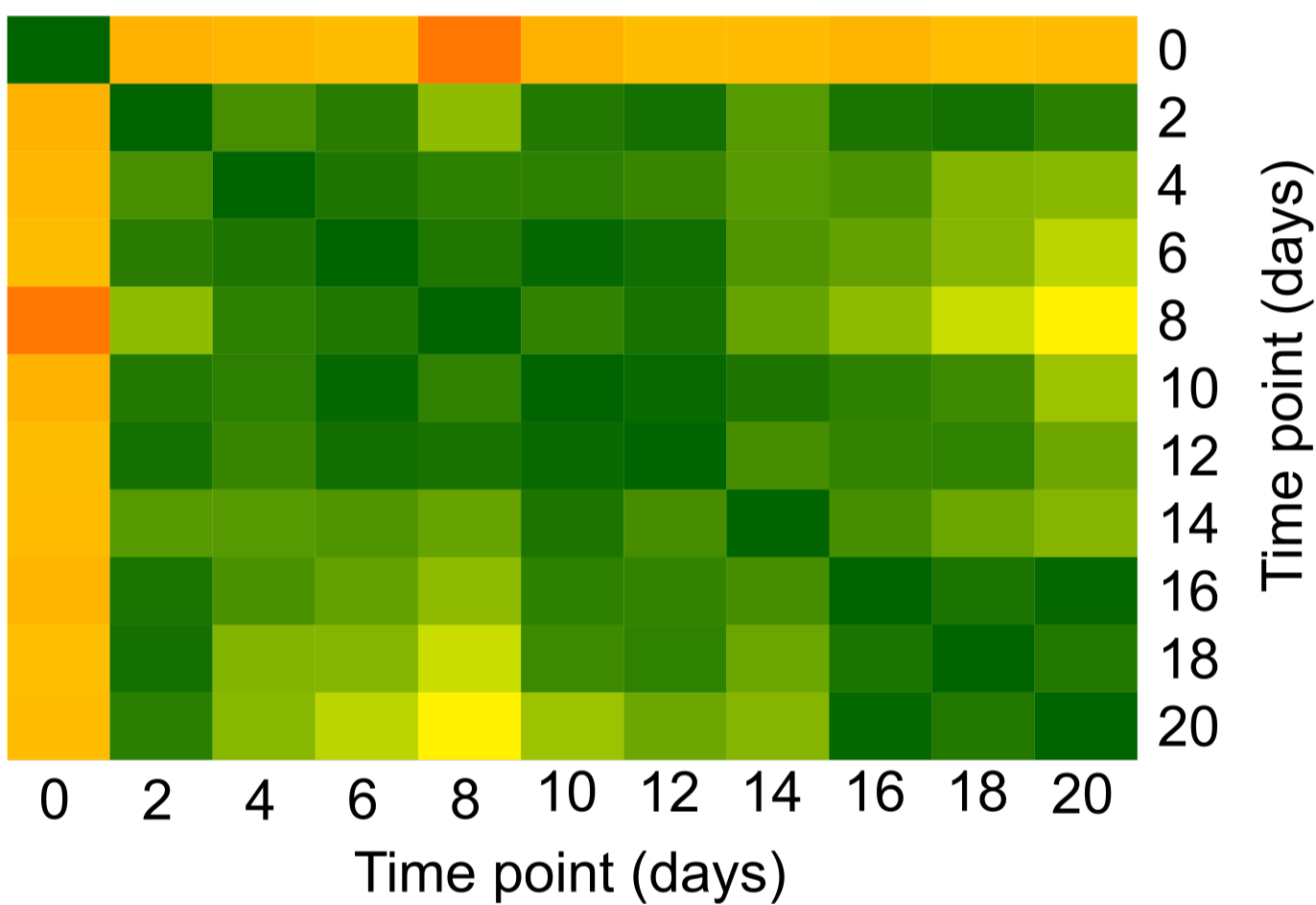**C**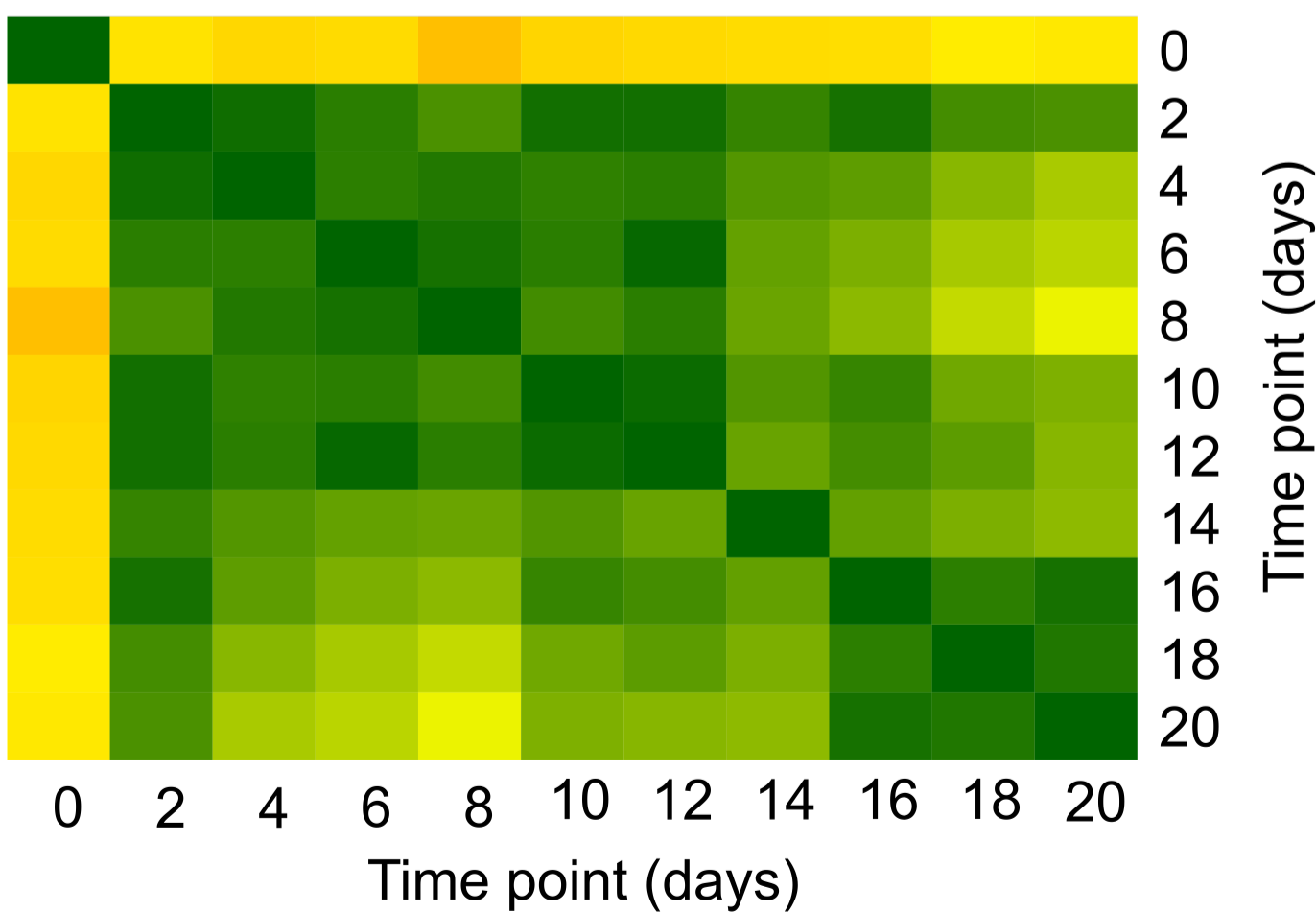**Color Key**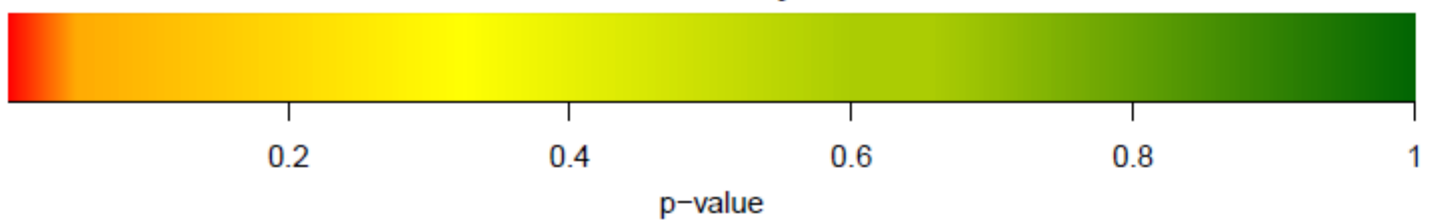

Supplement: Supplementary file 13 — Additional file 12: Figure S6. Stability within SalmoSim system calculated by using different UniFrac values for pairwise beta diversity analysis. The figure represents microbial stability within the SalmoSim system (data from all gut compartments and two different technical replicate runs combined) as the pairwise beta diversity comparison between different sampling time points (days), calculated by using unweighted (0%), generalised (50%) and weighted (100%) UniFrac as a distance measure. A small p-value indicates that the two time points are statistically different, and p>0.05 indicates that two time points are not statistically different. The colour key illustrates the p-value: red end of spectrum denoting low p values (distinct compositions between time points) and dark green indicating high p values (similar compositions between timepoints). [file 40168_2021_1134_MOESM13_ESM.pdf]

**A**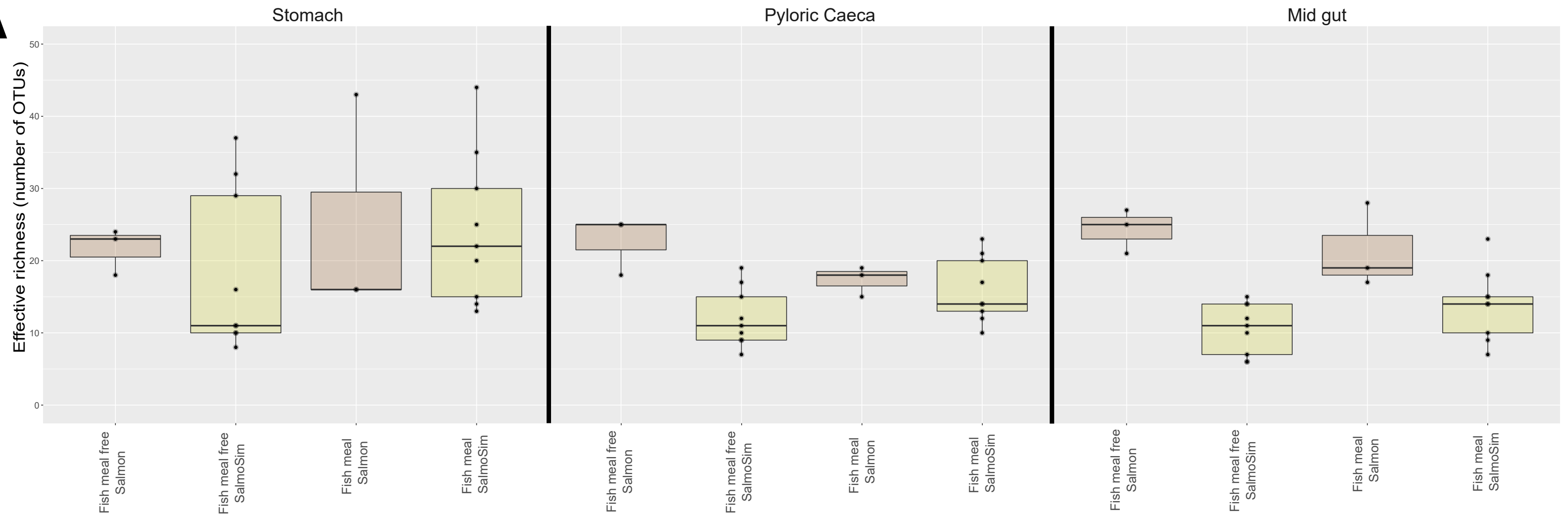**B**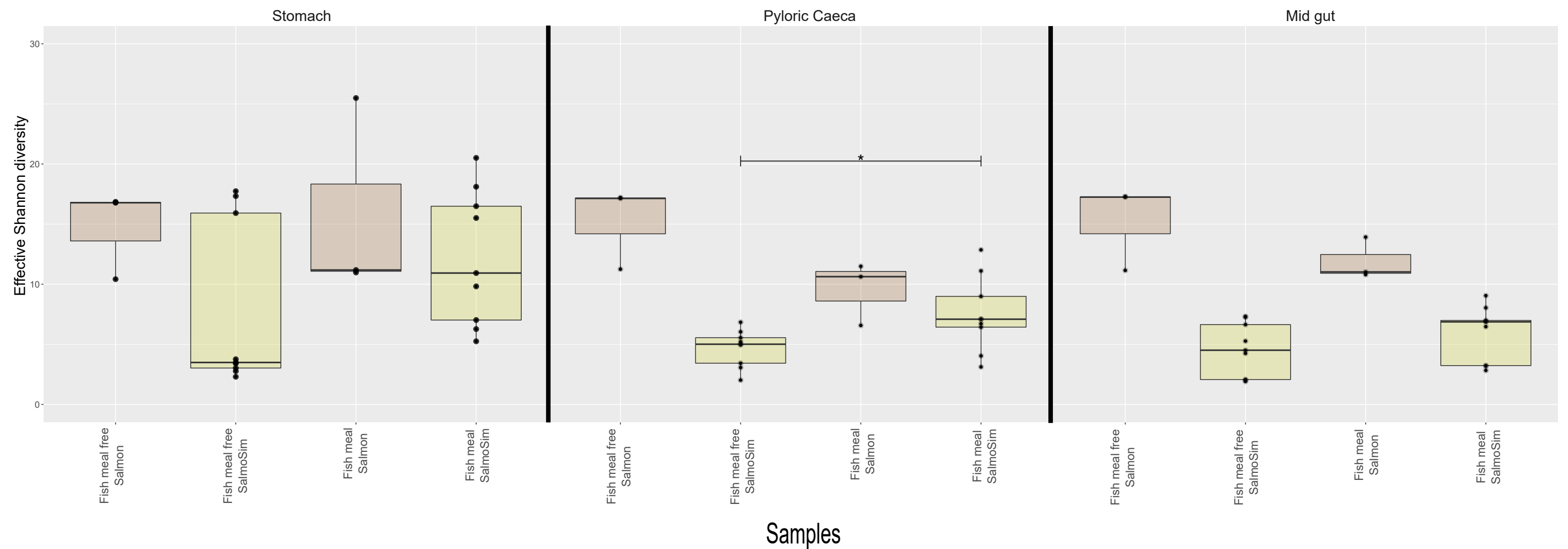

Supplement: Supplementary file 14 — Additional file 13: Figure S7. Calculated alpha-diversity metrics within different gut compartments of real salmon and SalmoSim fed on Fish meal and Fish meal free diets. Figure visually represents different alpha diversity outputs within different gut compartments of real salmon in red and SalmoSim in yellow (stable time points: 16, 18 and 20 fed on Fish meal, and 36, 38 and 40 fed on Fish meal free diet) fed on Fish meal and Fish meal free diets. A visually represents effective richness (number of OTUs), B represents effective Shannon diversity. The lines above bar plots represent statistically significant differences after feed change. The stars flag the levels of significance: one star (*) for p-values between 0.05 and 0.01, two stars (**) for p-values between 0.01 and 0.001, and three stars (***) for p-values below 0.001. [file 40168_2021_1134_MOESM14_ESM.pdf]

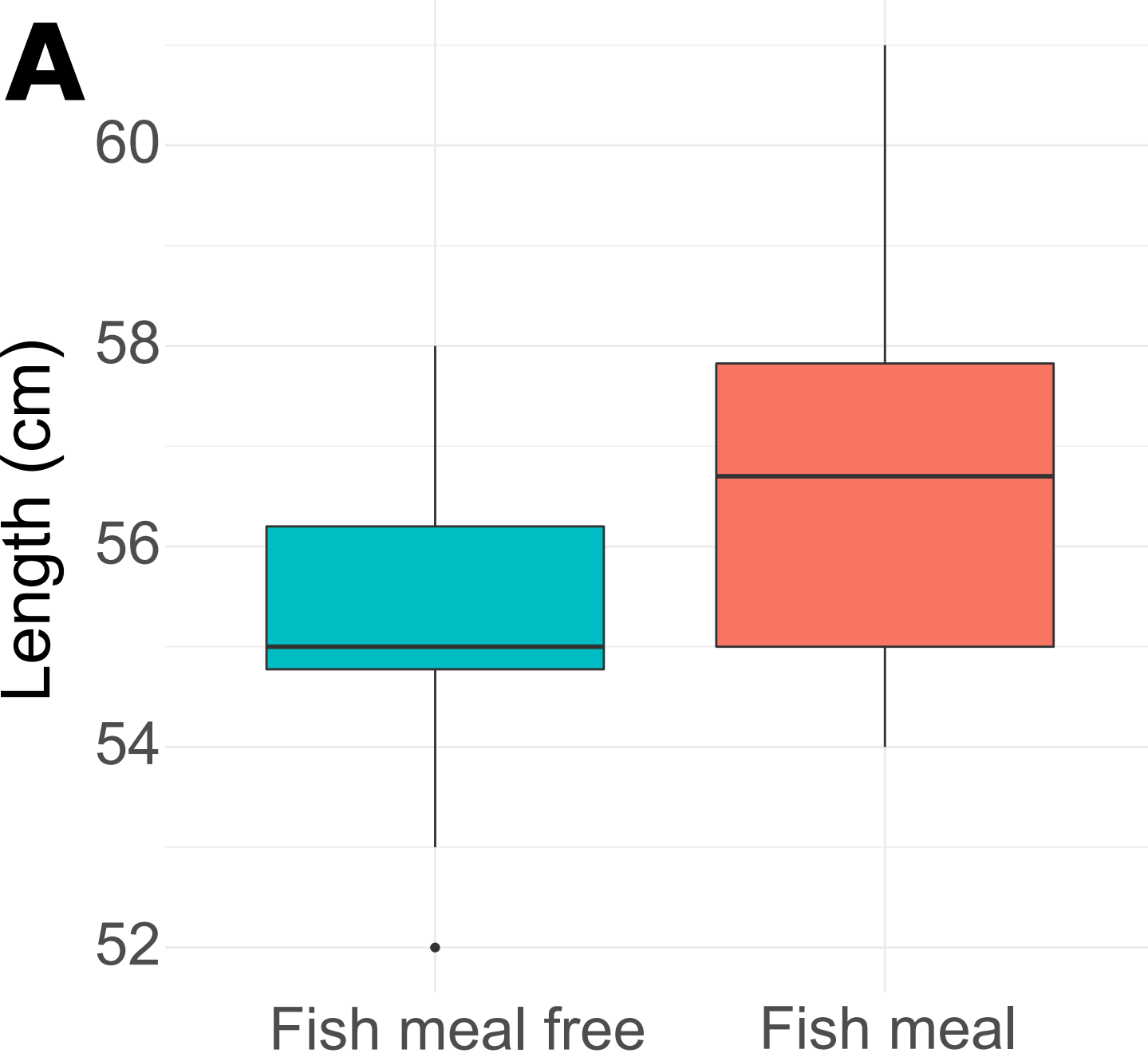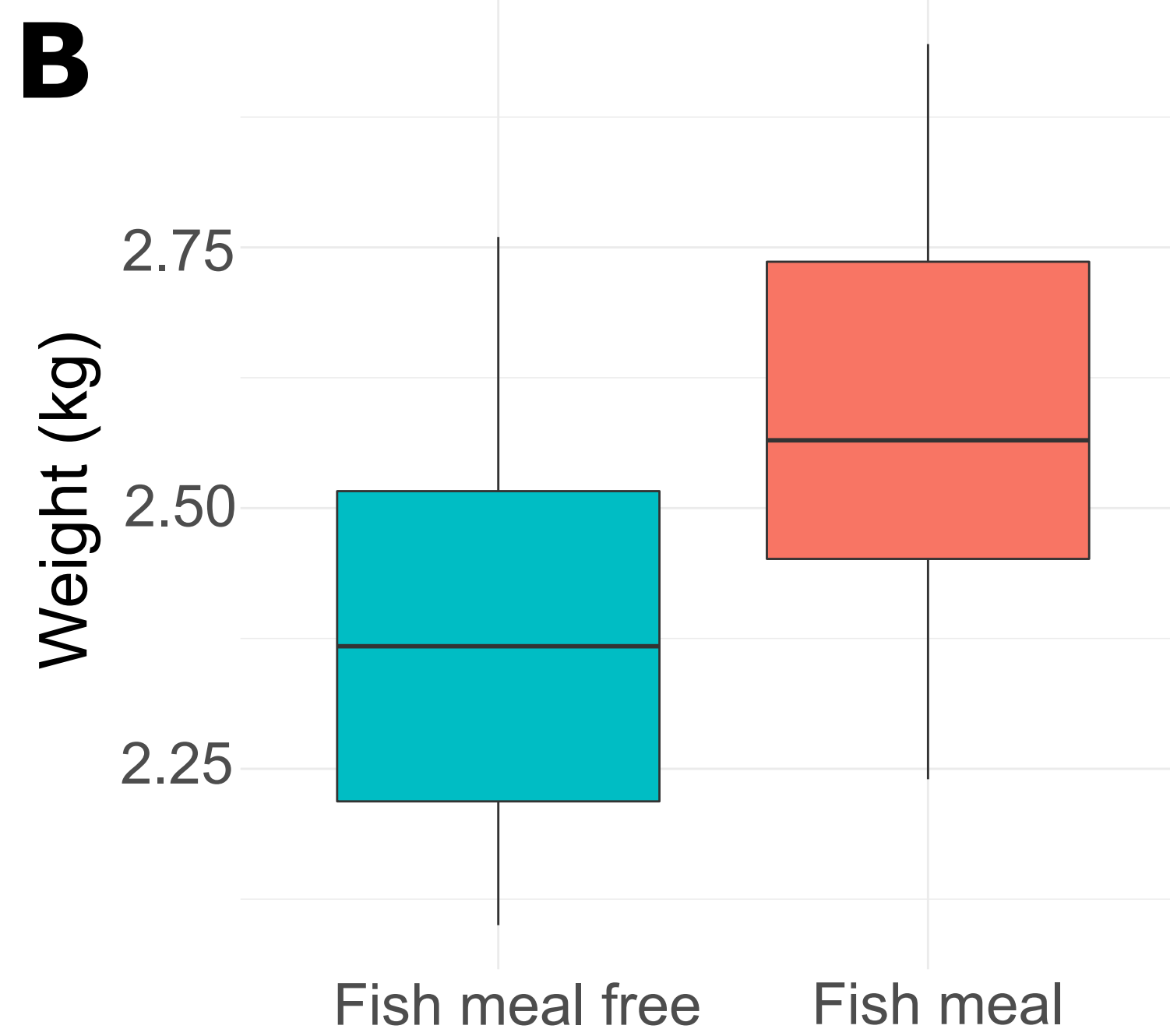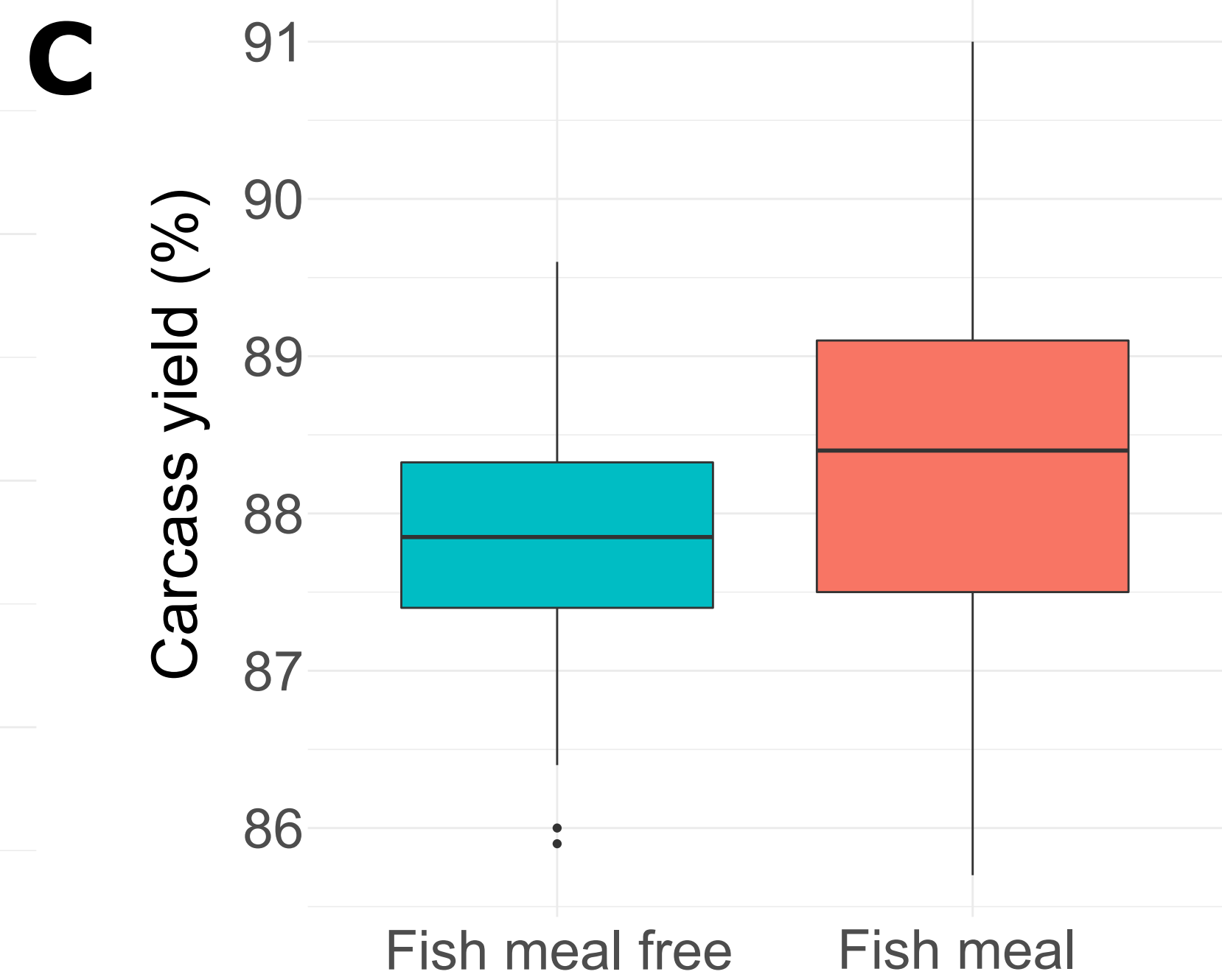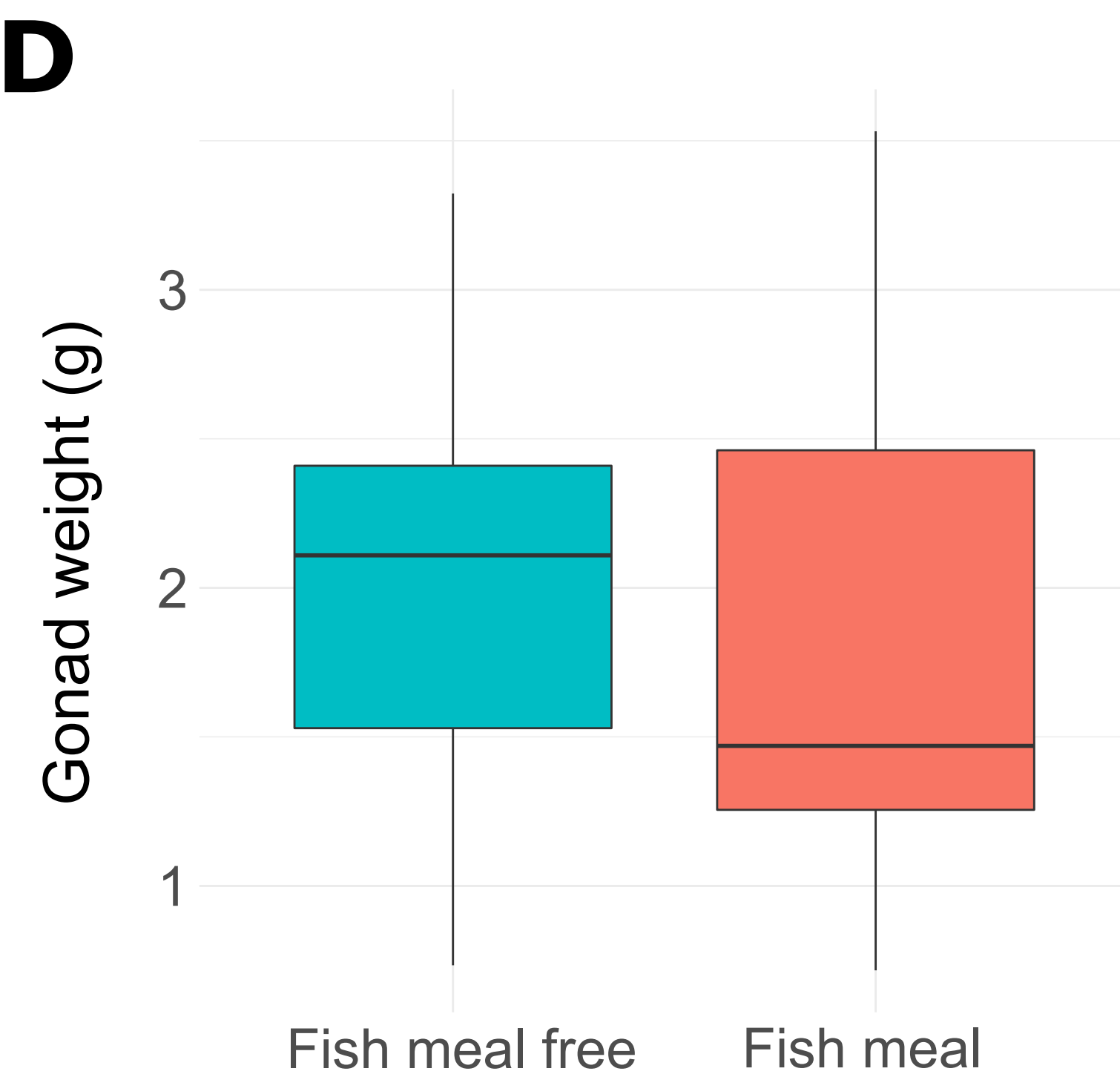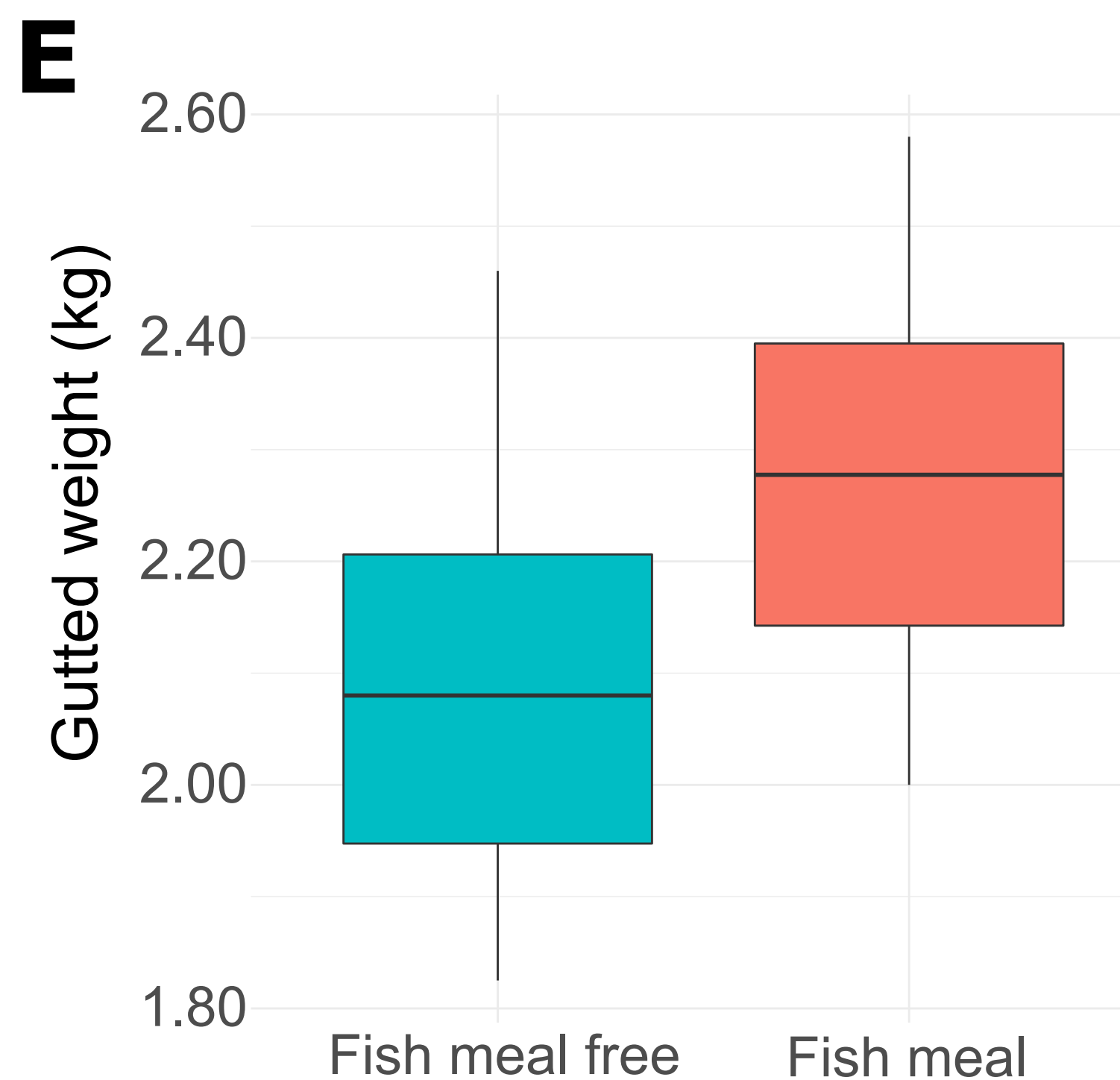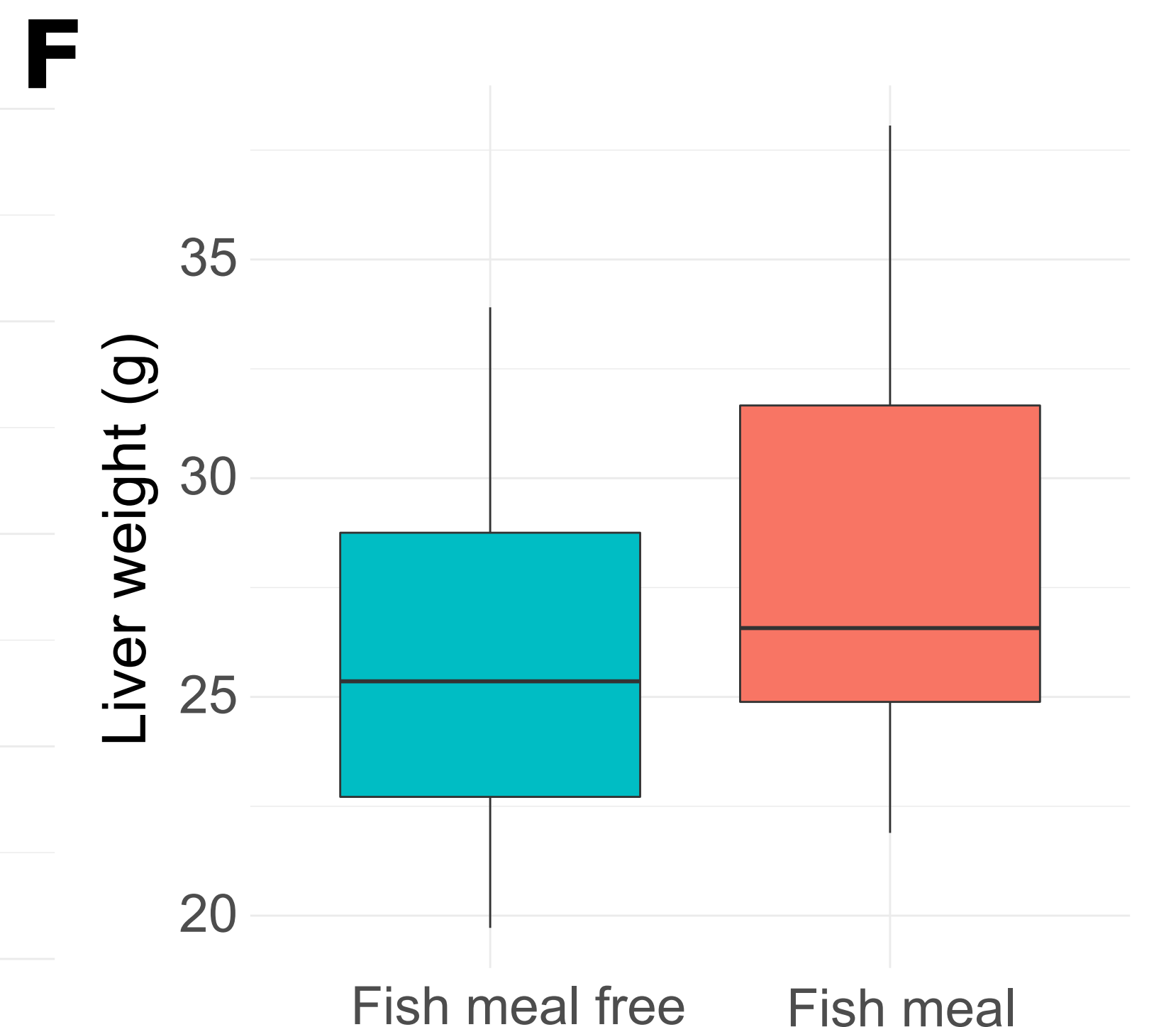

Supplement: Supplementary file 15 — Additional file 14: Figure S8.In vivo phenotypic fish performance fed on two different feeds. Figure visually represents different phenotypic performance data of fish (n=32 per feed) fed on two different feed. A Atlantic salmon length in centimetres; B Atlantic salmon length in weight in kilograms; C Atlantic salmon percentage carcass yield; D Atlantic salmon gonad weight in grams; E Atlantic salmon gutted weight in kilograms; F Atlantic salmon liver weight in grams. Blue box plots represent data from salmon (n=32) fed on Fish meal free diet, and red represents Atlantic salmon fed on Fish meal diet (n=32). [file 40168_2021_1134_MOESM15_ESM.pdf]
