# Supplementary material for: SalmoSim: the development of a three-compartment in vitro simulator of the Atlantic salmon GI tract and associated microbial communities
Source: Microbiome. 2021 Aug 31;9:179. doi: 10.1186/s40168-021-01134-6 (PMC8408954; doi:10.1186/s40168-021-01134-6)
Supplement: Supplementary file 10 — Additional file 9: Figure S3. Stability within SalmoSim system calculated by using unweighted and weighted UniFrac values for pairwise beta diversity analysis. The figure represents microbial stability within the SalmoSim system (data from all gut compartments combined) as the pairwise beta diversity comparison between different sampling time points (days), calculated by using A unweighted (0%) and B weighted (100%) UniFrac as a distance measure. A small p-value indicates that the two time points are statistically different, and p>0.05 indicates that two time points are not statistically different. The colour key illustrates the p-value: red end of spectrum denoting low p values (distinct compositions between time points) and dark green indicating high p values (similar compositions between timepoints). [file 40168_2021_1134_MOESM10_ESM.pdf]

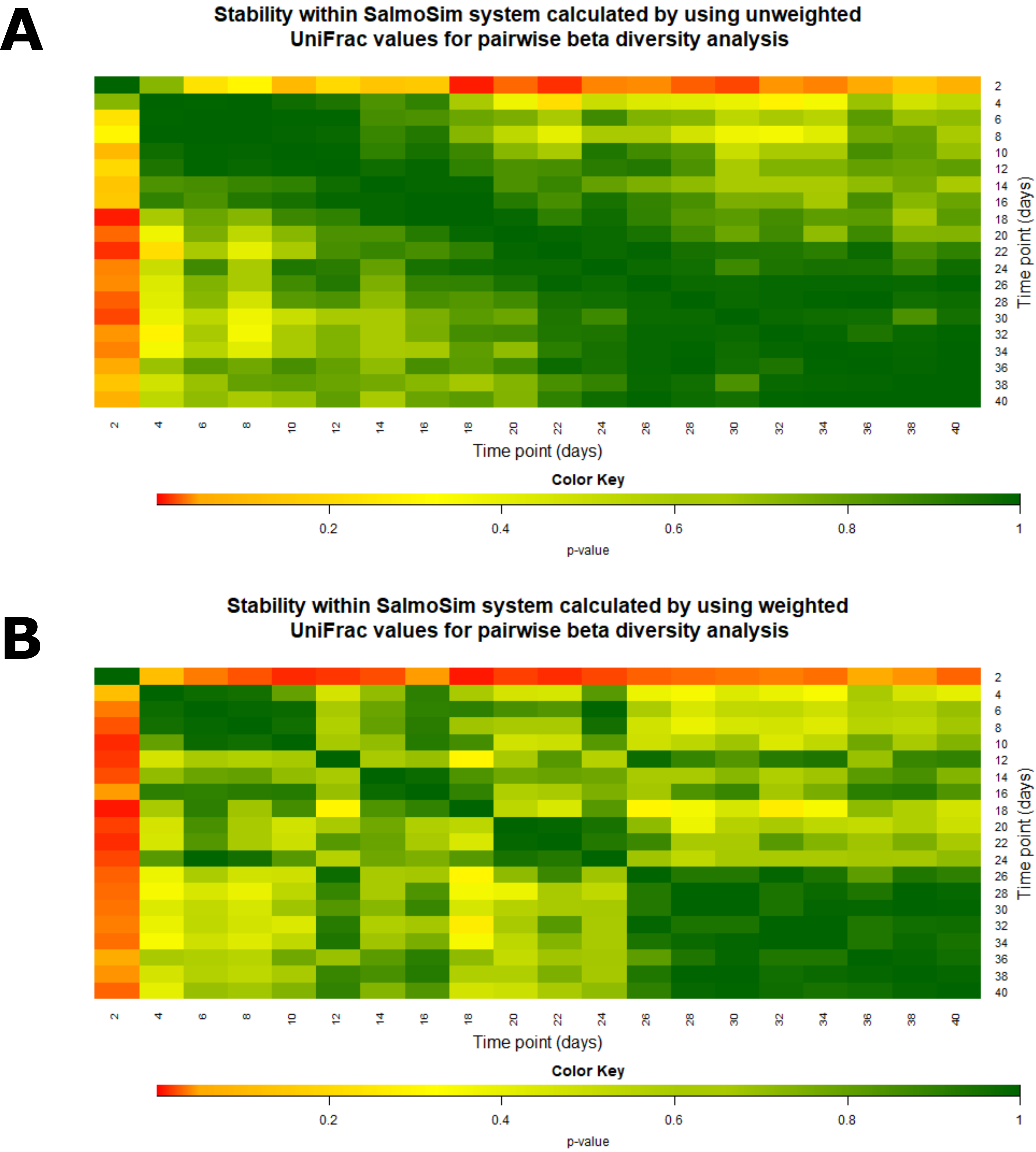
A
Stability within SalmoSim system calculated by using unweighted UniFrac values for pairwise beta diversity analysis
Time point (days)
Time point (days)
Color Key
0.2
0.4
0.6
0.8
1
p-value
B
Stability within SalmoSim system calculated by using weighted UniFrac values for pairwise beta diversity analysis
Time point (days)
Time point (days)
Color Key
0.2
0.4
0.6
0.8
1
p-value
