# Supplementary material for: SalmoSim: the development of a three-compartment in vitro simulator of the Atlantic salmon GI tract and associated microbial communities
Source: Microbiome. 2021 Aug 31;9:179. doi: 10.1186/s40168-021-01134-6 (PMC8408954; doi:10.1186/s40168-021-01134-6)
Supplement: Supplementary file 16 — Additional file 15: Figure S9. VFA production within different SalmoSim compartments fed on different feeds. Figure represents 11 volatile fatty acid production within SalmoSim system fed on Fish meal and Fish meal free diets within different gut compartments. Y axis represents the concentration of specific volatile fatty acid (mM) while the X axis represents each gut compartment (stomach, pyloric caeca, midgut). Red colour denoted Fish meal and blue – Fish meal free diets. The lines above bar plots represent statistically significant differences between different feeds and gut compartments. The stars flag the levels of significance: one star (*) for p-values between 0.05 and 0.01, two stars (**) for p-values between 0.01 and 0.001, and three stars (***) for p-values below 0.001. [file 40168_2021_1134_MOESM16_ESM.pdf]

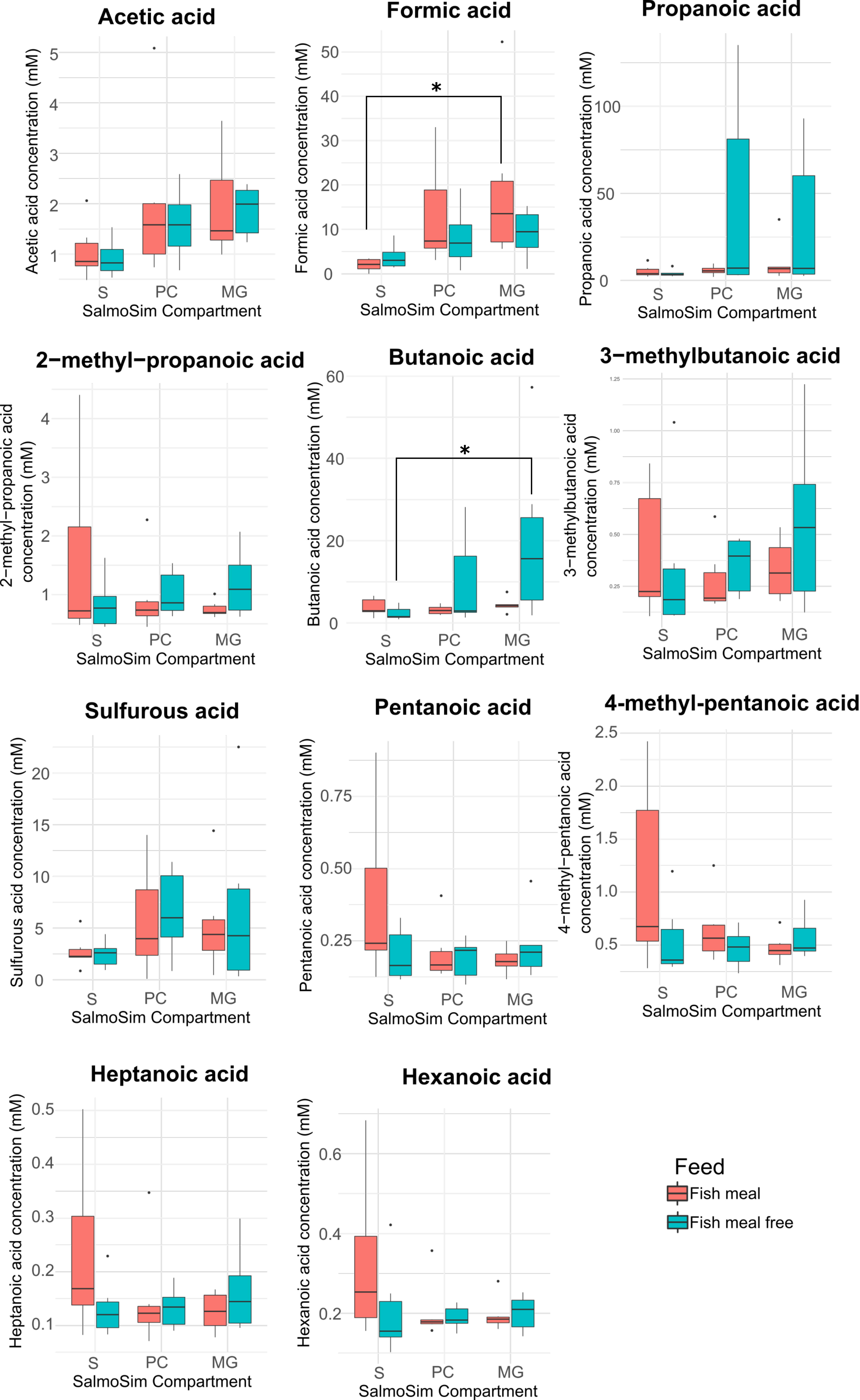

Acetic acid
Acetic acid concentration (mM)
1
2
3
4
5
S
PC
MG
SalmoSim Compartment
Formic acid
Formic acid concentration (mM)
0
10
20
30
40
50
*
S
PC
MG
SalmoSim Compartment
Propanoic acid
Propanoic acid concentration (mM)
0
50
100
S
PC
MG
SalmoSim Compartment
2−methyl−propanoic acid
2−methyl−propanoic acid concentration (mM)
1
2
3
4
S
PC
MG
SalmoSim Compartment
Butanoic acid
Butanoic acid concentration (mM)
0
20
40
60
*
S
PC
MG
SalmoSim Compartment
3−methylbutanoic acid
3−methylbutanoic acid concentration (mM)
0.25
0.50
0.75
1.00
1.25
S
PC
MG
SalmoSim Compartment
Sulfurous acid
Sulfurous acid concentration (mM)
0
5
10
15
20
S
PC
MG
SalmoSim Compartment
Pentanoic acid
Pentanoic acid concentration (mM)
0.25
0.50
0.75
S
PC
MG
SalmoSim Compartment
4-methyl-pentanoic acid
4−methyl−pentanoic acid concentration (mM)
0.5
1.0
1.5
2.0
2.5
S
PC
MG
SalmoSim Compartment
Heptanoic acid
Heptanoic acid concentration (mM)
0.1
0.2
0.3
0.4
0.5
S
PC
MG
SalmoSim Compartment
Hexanoic acid
Hexanoic acid concentration (mM)
0.2
0.4
0.6
S
PC
MG
SalmoSim Compartment
Feed
Fish meal
Fish meal free
